# Dietary Amino Acids and Risk of Stroke Subtypes: Results from 3 Large Prospective Cohort Studies

**DOI:** 10.1016/j.tjnut.2025.03.026

**Published:** 2025-03-22

**Authors:** Tammy YN Tong, Yanping Li, Kathryn M Rexrode, Walter C Willett, Qi Sun, JoAnn E Manson, Valter D Longo, Timothy J Key, Frank B Hu

**Affiliations:** 1 Cancer Epidemiology Unit, Nuffield Department of Population Health, University of Oxford, Oxford, United Kingdom; 2 Department of Nutrition, Harvard T.H. Chan School of Public Health, Boston, MA, United States; 3 Division of Women’s Health, Department of Medicine, Brigham and Women’s Hospital and Harvard Medical School, Boston, MA, United States; 4 Channing Division of Network Medicine, Department of Medicine, Brigham and Women's Hospital and Harvard Medical School, Boston, MA, United States; 5 Department of Epidemiology, Harvard T.H. Chan School of Public Health, Boston, MA, United States; 6 Division of Preventive Medicine Brigham and Women's Hospital and Harvard Medical School, Boston, MA, United States; 7 Longevity Institute, Davis School of Gerontology and Department of Biological Sciences, University of Southern California, Los Angeles, CA, United States; 8 IFOM ETS, AIRC Institute of Molecular Oncology, Milan, Italy

**Keywords:** amino acids, stroke, protein, nutritional epidemiology, prospective cohort

## Abstract

**Background:**

Differences in dietary protein have been associated with stroke risk, with possible heterogeneity in associations by stroke type or food sources of protein.

**Objectives:**

We examined the associations of individual dietary amino acids, as the constituents of dietary protein, with risks of ischemic, hemorrhagic, and total stroke.

**Methods:**

We analyzed data from 73,830 females in the Nurses’ Health Study (1984–2012), 92,333 females in the Nurses’ Health Study II (1991–2013), and 43,268 males in the Health Professionals Follow-Up Study (1986–2016). Dietary intakes of 22 (20 standard and 2 nonstandard) amino acids were assessed using validated food frequency questionnaires, administered typically every 4 y. Multivariable-adjusted Cox regression models were used to estimate hazard ratios (HRs) and 95% confidence intervals (CIs) of ischemic, hemorrhagic, and total stroke in relation to the energy-adjusted intakes of individual amino acids.

**Results:**

During a mean follow-up of 23.7 y, 3058 ischemic, 872 hemorrhagic, and 5997 total stroke cases were documented. After correction for multiple testing, lower risks of ischemic stroke were observed with higher intakes of glutamine (HR per 1 standard deviation higher: 0.94, 95% CI: 0.90, 0.98, *P* = 0.004) and proline (0.94, 0.90, 0.98, *P* = 0.005). The associations remained directionally consistent across sensitivity analyses but attenuated upon mutual adjustment. All other amino acids, including branched-chain amino acids, were not significantly associated with ischemic stroke. For hemorrhagic stroke, no significant associations were observed for any of the amino acids. For total stroke, inverse associations were also observed for both glutamine (0.94, 0.91–0.97, *P* < 0.001) and proline (0.96, 0.93–0.99, *P* = 0.004). In terms of dietary sources, glutamine was most strongly correlated with plant protein and whole grains, whereas proline was most strongly correlated with dairy protein and dairy products.

**Conclusions:**

Higher intakes of glutamine and proline were associated with lower risks of ischemic and total stroke.

## Introduction

Differences in dietary protein intake have been associated with stroke risk, but existing evidence has been inconsistent, possibly owing to differences in protein sources and stroke type [1]. The various food sources of protein are made up of different compositions of dietary amino acids [2]. For example, although meat and meat products are rich in all essential amino acids, grain products are comparatively lower in essential amino acids, but richer in certain other amino acids such as cysteine, proline, and glutamic acid, and dairy products are also rich in proline [2]. Therefore, the varying amino acid compositions of different protein sources may be relevant for the differences in stroke risk observed. Additionally, the 2 main pathologic stroke types, ischemic and hemorrhagic stroke, have been linked with some key risk factors in opposite directions [3,4]. For example, higher circulating LDL cholesterol and higher BMI have both been associated with higher ischemic, but lower hemorrhagic stroke risk [3,4]. Likewise, dietary risk factors for the 2 stroke types have also been demonstrated to be different [5]. For example, higher intakes of plant protein or plant-sourced foods have been associated with a lower risk of ischemic stroke [5,6], whereas higher intakes of animal protein or animal-sourced foods have been associated with a lower risk of hemorrhagic stroke in some studies [[6], [7], [8]]. Hence, differential associations of dietary amino acids by stroke types may be expected, and additional research should focus on examining stroke types separately. This is especially considering that specific amino acids also have a range of roles in cellular function in addition to their contribution to protein synthesis, such as the role of glutamine as a major energy source for proliferating cells [9].

Nonetheless, evidence on dietary amino acids and stroke risk is limited. Previous analyses in the European Prospective Investigation into Cancer and Nutrition (EPIC) study have found that higher intake of proline was associated with a lower risk of ischemic stroke [10], whereas a study from the Swedish Mammography Cohort reported an inverse association between dietary cysteine and risk of total stroke [11]. Both the EPIC Study [10] and the Takayama Study in Japan [12] also reported an inverse association of glutamic acid with risk of ischemic stroke and total stroke mortality, respectively. However, no other studies exist on the topic, and the existing studies have not comprehensively examined all amino acids with both stroke types. To address this gap, we assessed the associations of all amino acids with ischemic, hemorrhagic, and total stroke in 3 large prospective cohorts of females and males in the United States.

## Methods

### Study population and data collection

The study population included 3 prospective cohorts in the United States: the Nurses’ Health Study (NHS), the Nurses’ Health Study II (NHSII), and the Health Professionals Follow-Up Study (HPFS). The NHS recruited 121,701 female nurses aged 30–55 y in 1976, the NHSII recruited 116,429 female nurses aged 25–42 y in 1989, whereas the HPFS recruited 51,529 male health professionals aged 40–75 y in 1986. At recruitment, all participants completed a self-administered questionnaire that asked about their sociodemographics, lifestyle, medical history, and other health-related factors. Similar follow-up questionnaires which asked for updated information were mailed to participants every 2 y subsequently, with a response rate of ≈90%. Diet was assessed using validated semiquantitative food frequency questionnaires (FFQs) in each of the 3 cohorts; further details of the dietary assessment are described in the following section. The study protocol was approved by the institutional review boards of Brigham and Women’s Hospital and the Harvard T.H. Chan School of Public Health. Return of the self-administered questionnaires was considered to imply informed consent.

For these analyses, we excluded participants with a history of cardiovascular diseases or cancer at the earliest availability of comprehensive dietary data (that is, the baseline of these analyses, 1984 for NHS, 1991 for NHSII, 1986 in HPFS, further details below), or those who died before baseline. We also excluded participants who did not complete the baseline FFQs, those who had missing dietary data or implausible energy intakes (<600 or >3500 kcal/d for females in NHS and NHSII and <800 or >4200 kcal/d for males in HPFS), as well as those who only completed the baseline survey or had missing age. After exclusions, the current analyses included 73,830 females from NHS, 92,333 females from NHSII, and 43,268 males from HPFS. Further details of the inclusion and exclusion criteria are shown in Supplemental Figure 1.

### Assessment of dietary amino acids

Diet was assessed using validated semiquantitative FFQs, which asked participants about their consumption frequency of specific foods of a standard portion size over the past year, with possible responses ranging from never or <1 serving per month to 6 or more servings per day. In NHS, diet was first assessed in 1980 using a 61-item FFQ, which was subsequently expanded to include 116 items in 1984 and 131 items from 1991 onwards [13]. The same expanded questionnaires were sent to NHS participants in 1984 and 1986, to NHS II participants in 1991 when diet was first assessed, to HPFS participants in 1986, and every 4 y subsequently in each of the 3 cohorts. The dietary questionnaire used in NHS in 1980 did not allow a comprehensive assessment of all dietary amino acids, and therefore 1984 was considered the study baseline in NHS.

On the basis of the participants’ responses on the FFQs, estimates of nutrient intakes including total energy, macronutrients, and intakes of most dietary amino acids were derived according to the USDA database and Harvard University food composition databases, which are periodically updated. The amino acids available in the USDA nutrient database included all branched-chain amino acids (isoleucine, leucine, and valine) and other essential amino acids (histidine, lysine, methionine, phenylalanine, threonine, and tryptophan), 9 of the nonessential amino acids (alanine, arginine, aspartic acid, cystine, glutamic acid, glycine, proline, serine, and tyrosine), and 2 nonstandard amino acids (hydroxyproline, taurine). Estimates of asparagine and glutamine were not available from the USDA database, as these 2 amino acids were affected by deamination reactions during the acid hydrolysis process used to compile the USDA database, which resulted in their conversion to and therefore overestimation of aspartic acid and glutamic acid in this database [14]. As a result, estimates for these 4 amino acids (asparagine, aspartic acid, glutamine, and glutamic acid) were derived using a gene sequencing method (Swiss Institute of Bioinformatics) instead; the method and associated estimates have been validated against USDA and modified biochemical method [14]. In the USDA database, cystine, an oxidized dimeric form of cysteine, is available instead of cysteine itself, but estimates are similar to cysteine derived using the gene sequencing method and therefore the values of cystine derived from the USDA database are used in these analyses.

For our primary analyses, dietary intakes of the amino acids were adjusted for total energy intake using the residual method and presented as adjusted grams of intake per day. Secondarily, we also included analyses of unadjusted grams of intake of amino acids per day, to test the hypothesis that absolute intakes of dietary amino acids would be relevant for risk. Cumulatively averaged intakes of amino acids from all available dietary questionnaires were used, to better reflect long-term diet and reduce measurement error. As participants may have altered their diet after diagnosis of a major illness, the dietary variables were not updated when participants reported a diagnosis of coronary artery disease (myocardial infarction, angina, or coronary artery surgery), cancer, or diabetes. In addition, data on hydroxyproline and taurine were not available in 2010 in NHS, 2011 in NHSII, and 2010 and 2014 in HPFS, and hence intakes of these 2 amino acids were not updated at these time points.

### Assessment of stroke outcomes

The outcomes of interest were ischemic stroke (thrombotic, embolic, or unspecified nonhemorrhagic stroke), hemorrhagic stroke (intraparenchymal and subarachnoid stroke), and total stroke (ischemic, hemorrhagic, and unknown stroke type), as defined according to criteria in the National Survey of Stroke [15]. Incident nonfatal stroke cases were identified based on self-reported physician diagnoses of stroke events on the biennial questionnaires. Participants who reported an incident stroke were asked to provide additional details by letter or interview and for permission to obtain medical records to confirm their diagnosis of stroke by study physicians. The nonfatal stroke cases with confirmation that the self-report was accurate by letter or interview, but which lacked adequate medical documentation, were considered participant-corroborated or “probable” cases. Fatal events were identified by reports from family, by the United States Postal Service, or by search of the National Death Index, and 98% of the deaths in each cohort were ascertained [16]. Confirmation of fatal stroke cases relied on death certificates, medical records during hospitalization, or autopsy records. Both confirmed and probable stroke cases were included in all analyses.

### Assessment of covariates

Information on the participants’ sociodemographic characteristics (ethnicity, marital status), lifestyle (smoking, physical activity), anthropometry (body weight, height at recruitment), medical history (history or hypertension, hypercholesterolemia and diabetes), and use of medications and supplements (aspirin and multivitamins) was collected using the biennial questionnaires, and updated information was used in the analyses where appropriate. In females (NHS and NHSII), questions were also asked about their menopausal status, and the use of postmenopausal hormone therapy and oral contraceptives (the latter for NHSII only). BMI was calculated as the weight in kilograms divided by height in meters squared (kg/m^2^). Physical activity was quantified based on the metabolic equivalent of task values of different activity types. The validity of the body weight and physical activity measures has been described in detail previously [17,18]. Diet-related covariates, including total energy intake, alcohol consumption, macronutrients, major food groups, and the Alternate Healthy Eating Index (AHEI) score [19] were derived based on responses from the FFQs.

### Statistical analyses

Cohort characteristics and dietary intakes of amino acids at baseline were summarized as means (SD) or numbers (%). Spearman correlation coefficients were estimated between the individual dietary amino acids, and of dietary amino acids with macronutrients and major food groups. Person-years of follow-up were calculated from the return date of the baseline FFQ (1984 in NHS, 1991 in NHSII, and 1986 in HPFS) until the date of stroke diagnosis, death, or the end of follow-up (June 2012 in NHS, June 2013 in NHSII, and January 2016 in HPFS, based on the last complete cycle of medical record coding for stroke subtypes), whichever came first. Cox regressions were used to estimate hazard ratios (HRs) and 95% confidence intervals (CIs) for the associations of dietary amino acids with stroke outcomes, modeled as both fifths and per SD differences of energy-adjusted intakes.

In the minimally adjusted model, the analyses were stratified by age (in years) and follow-up intervals (every 2 y). The first multivariable-adjusted model was additionally adjusted for ethnicity (White, non-White), marital status (married, widowed, divorced/separated, and unknown), smoking status [never, past, current: 1–14, 15–24, ≥25 cigarettes/day, unknown (either unknown status or unknown cigarette number)], alcohol intake (never drinkers, 0.1–4.9, 5.0–9.9, 10.0–14.9, 15.0+ g/d), physical activity (<3, 3–9, 9–18, 18–27, 27–42, ≥42 metabolic equivalents/week, unknown), combined menopausal status and postmenopausal hormone use (premenopausal, postmenopausal: never, past, current users, NHS and NHSII only), oral contraception use (never, past, current user, NHSII only), multivitamin use (no, yes), aspirin use (no, yes), BMI (<23, 23–24.9, 25–29.9, 30–34.9, and ≥35 kg/m^2^, missing), baseline history of hypertension (no, yes), baseline hypercholesteremia (no, yes), baseline diabetes (no, yes), and energy intake (fifths of intake), and was considered the main model in these analyses. The second multivariable model additionally adjusted for the AHEI (fifths of score), to evaluate the potential influence of overall diet quality. For each association, the *P*-trend was estimated by fitting the median values of each fifth of intake as a pseudocontinuous variable in the Cox regression when reporting the results by fifths of intake, whereas the *P* value was estimated by fitting continuous values of intake in the Cox regression when reporting the results by per SD differences. The analyses were conducted separately in each cohort, and results from NHS, NHSII, and HPFS were pooled using a fixed-effect meta-analysis across all cohort-specific analyses.

As secondary analyses, we repeated the analyses using nonenergy-adjusted intakes of dietary amino acids. To evaluate potential reverse causation, we conducted a latency analysis with a lag period of 4 y, that is, estimates of dietary amino acid intake from the 1986 questionnaire were used for evaluating stroke outcomes occurring between 1990 and 1994, and so on. To assess potential heterogeneity of the associations by subgroups of the known major confounders between diet and stroke risk and to assess residual confounding, we also conducted stratified analyses by age (<55 y, ≥55 y), BMI (normal weight <25 kg/m^2^, overweight ≥25 kg/m^2^), smoking status (never smokers, ever smokers), alcohol consumption (never or occasional drinkers <5 g alcohol/d, regular drinkers ≥5 g alcohol/d), physical activity (below and above median), multivitamin use (no, yes) and history of hypertension, high blood cholesterol or diabetes (no disease history, history of 1 or more conditions). The *P* value for interaction between dietary amino acids and the stratifying variable was examined using the Wald test with 1 degree of freedom, using a multivariable-adjusted Cox model with an interaction term of the amino acid and stratifying variable of interest. To investigate whether any observed significant associations for the amino acids were independent of other amino acids, macronutrients, and the major food sources for these amino acids, we included additional models mutually adjusting for each of the other dietary amino acids or dietary factors, 1 at a time.

All tests for statistical significance were 2-sided, and conventional *P* values are reported throughout. To account for multiple testing while allowing for the high correlations between dietary amino acids [20,21], we conducted a principal component analysis of the exposure variables and determined that the first 7 principal components explained over 99% of the variation in the exposure data. On the basis of this, the effective number of independent tests was determined to be 7, and the statistical significance level corrected for multiple testing was defined as 0.05/7 = 0.0071. All statistical analyses were performed using SAS for UNIX version 9.4 (SAS Institute), and the forest plots were generated using the “Jasper makes plots” package version 2-266 [22] in R version 4.2.1.

## Results

### Baseline and dietary characteristics

The baseline characteristics of study participants in NHS, NHSII, and HPFS are shown in Table 1, whereas baseline intakes of dietary amino acids are shown in Supplemental Table 1, as mean (SD) of both energy-adjusted and unadjusted intake, which were similar. In all 3 cohorts, the highest intakes were observed for glutamic acid (mean energy-adjusted intake in NHS: 6.8, NHSII: 7.8, HPFS: 8.3 g/d) and glutamine (NHS: 6.4, NHSII: 7.8, HPFS: 8.0 g/d), whereas the lowest intakes were observed for hydroxyproline (NHS: 0.3, NHSII: 0.3, HPFS: 0.4 g/day) and taurine (NHS: 0.1, NHSII: 0.2, HPFS: 0.2 g/d). As post hoc analyses, we also presented baseline characteristics in the 3 cohorts by fifths of amino acids significantly associated with stroke risk in the main analyses (Supplemental Tables 2–4).

**TABLE 1:** Baseline characteristics of participants in the Nurses’ Health Study (NHS), Nurses’ Health Study II (NHSII), and Health Professionals Follow-Up Study (HPFS).

Baseline characteristics^1^	NHS	NHSII	HPFS
Baseline year	1984	1991	1986
Number of participants	73,830	92,333	43,268
Age at baseline (y)	50.2 (7.2)	36.1 (4.7)	53.2 (9.5)
White ethnicity (%)	97.8	96.4	95.0
Currently married (%)	82.2	89.7	92.9
BMI (kg/m^2^)	25.0 (4.7)	24.6 (5.3)	25.5 (3.3)
Physical activity (MET-h/wk)	14.1 (21.1)	20.8 (27.1)	20.8 (24.9)
Smoking status (%)			
Never smoker	44.0	65.6	46.1
Past smoker	31.7	22.2	42.5
Current smoker	24.1	12.1	9.1
Alcohol consumption (g/d)	6.9 (11.2)	3.1 (6.1)	11.4 (15.4)
Current postmenopausal hormone use (%)	13.7	2.6	–
Current oral contraceptive use (%)	–	15.4	–
Aspirin use (%)	71.1	11.2	26.7
Multivitamin use (%)	36.9	43.9	41.8
Baseline diabetes (%)	2.8	1.0	2.5
Baseline hypertension (%)	21.0	6.3	19.9
Baseline high blood cholesterol (%)	7.9	14.5	10.5
Energy intake (kcal/d)	1744.9 (530.7)	1789.6 (547.6)	1994.7 (621.1)
Total protein^2^ (g/d)	71.5 (13.0)	86.5 (15.4)	92.2 (16.5)
Animal protein^2^ (g/d)	52.4 (13.9)	64.1 (16.6)	67.5 (17.7)
Plant protein (g/d)^2^	19.0 (4.3)	22.5 (5.0)	24.7 (6.2)
Dairy protein^2^ (g/d)	14.2 (7.5)	18.6 (9.1)	15.8 (9.1)
Saturated fat^2^ (g/d)	22.1 (4.6)	22.4 (4.9)	24.7 (6.1)
Monounsaturated fat^2^ (g/d)	22.5 (4.3)	24.0 (4.9)	27.4 (6)
Polyunsaturated fat^2^ (g/d)	11.8 (3.1)	11.3 (2.7)	13.2 (3.5)
Total carbohydrates^2^ (g/d)	185.1 (31.4)	224.7 (33.8)	234.1 (42.3)
Total fiber^2^ (g/d)	16.4 (4.8)	18.3 (5.5)	20.9 (7.0)

Abbreviation: MET, metabolic equivalent of task.

1 Values are means (SD) for continuous variables and percentages for categorical variables.

2 Adjusted for total energy using the residual method.

Spearman correlation coefficients between energy-adjusted grams of dietary amino acids based on dietary data collected at the mid-point of follow-up are shown in Supplemental Figure 2, whereas Spearman correlation coefficients of dietary amino acids with macronutrients and major food groups are shown in Supplemental Figure 3. Generally, most amino acids exhibited very high correlations (*ρ* ≈ 0.9) with the other amino acids, with the exception of glutamine, hydroxyproline, and taurine which generally showed moderate to high correlations (*ρ* ≈ 0.5–0.7). In terms of nutrients and food groups, animal protein and animal-sourced foods, particularly total meat, red meat, and poultry, showed moderate to high correlations with many amino acids, whereas dairy protein was particularly highly correlated with proline (*ρ* = 0.73). Plant protein and plant-sourced foods including grains and fruits were generally less correlated with most amino acids compared with animal protein, with the exception of glutamine which was modestly more highly correlated with plant protein (*ρ* = 0.53) than animal protein (*ρ* = 0.43).

### Associations of dietary amino acids with stroke risk

**FIGURE 1:**
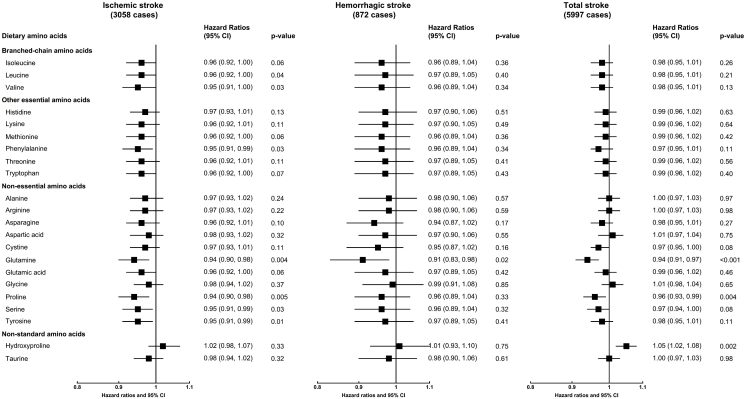
Hazard ratios [95% confidence intervals (CIs)] for ischemic, hemorrhagic and total stroke per SD higher intake of dietary amino acids. Intake of dietary amino acids was expressed as energy-adjusted grams of intake per day using the residuals method. Results from NHS, NHSII, and HPFS were pooled using a fixed-effect meta-analysis (4,964,284 person-years in total). Analyses in each study were stratified by age (in years) and follow-up intervals, and adjusted for ethnicity (White, non-White), marital status (married, widowed, divorced/separated, and unknown), smoking status (never, past, current: 1–14, 15–24, ≥25 cigarettes/d, unknown status or cigarette number), alcohol intake (never drinkers, 0.1–4.9, 5.0–9.9, 10.0–14.9, 15.0+ g/d), physical activity (<3, 3–9, 9–18, 18–27, 27–42, ≥42 metabolic equivalents/week, unknown), menopausal status and postmenopausal hormone use (premenopausal, postmenopausal: never, past, current users, NHS and NHSII only), oral contraception use (never, past, current user, NHSII only), multivitamin use (no, yes), aspirin use (no, yes), BMI (<23, 23–24.9, 25–29.9, 30–34.9, ≥35 kg/m^2^, missing), baseline history of hypertension (no, yes), baseline hypercholesteremia (no, yes), baseline diabetes (no, yes), and energy intake (fifths of intake). *P* values were estimated by fitting the continuous intakes of dietary amino acids in the Cox regression. HPFS, Health Professionals Follow-Up Study; NHS, Nurses’ Health Study; NHSII, Nurses’ Health Study II.

During a mean follow-up of 23.7 y (4,964,284 person-years), 3058 ischemic, 872 hemorrhagic, and 5997 total stroke cases were documented across the 3 cohorts. After multivariable adjustment and correction for multiple testing, higher intakes of glutamine (HR per 1 SD higher: 0.94, 95% CI: 0.90, 0.98, *P* =0.004) and proline (0.94; 0.90, 0.98, *P* = 0.005) were each associated with lower risk of ischemic stroke (Figure 1). The results were consistent when examining fifths of intake (top compared with bottom fifth for glutamine 0.86; 0.77, 0.97, *P*-trend = 0.007, for proline 0.85; 0.76, 0.96, *P*-trend = 0.002) (Supplemental Table 5). No other statistically significant associations were observed for ischemic stroke, and none of the amino acids was statistically significantly associated with hemorrhagic stroke, though a nonstatistically significant but inverse association was also observed for glutamine (0.91, 0.83, 0.98, *P* = 0.02) (Figure 1, Supplemental Table 6). For total stroke, inverse associations were observed for per SD higher of both glutamine (0.94, 0.91–0.97 *P* <0.001) and proline (proline: 0.96, 0.93–0.99, *P* = 0.004), whereas higher risk was observed with higher intakes of hydroxyproline (1.05, 1.02–1.08, *P* = 0.002) (Figure 1); results were also consistent when comparing top to bottom fifths of intake (glutamine, 0.87; 0.80, 9.94, *P*-trend < 0.001; proline, 0.90, 0.83–0.98, *P*-trend = 0.004; hydroxyproline, 1.15; 1.06, 1.24, *P*-trend < 0.001) (Supplemental Table 7). The results were directionally consistent and similar in magnitude across different adjustment models (Supplemental Tables 8–10) but did not meet the threshold of significance for multiple testing for the association for glutamine (*P* = 0.01) and proline (*P* = 0.009) with ischemic stroke (Supplemental Table 8) or proline (*P* = 0.02) or hydroxyproline (*P* = 0.01) with total stroke (Supplemental Table 10) after adjusting for overall diet quality (AHEI). Results were also similar when we modeled the unadjusted grams of amino acids (Supplemental Tables 11–13), and directionally consistent in the 4-y lag analyses, albeit with wider confidence intervals and not always statistically significant likely reflecting the lower number of cases in these analyses (Supplemental Table 14).

Results from analyses mutually adjusting for each of the other amino acids are shown for the significant amino acids from the main analyses, in Table 2 and Supplemental Table 15. For ischemic stroke (Table 2), the associations for both glutamine and proline attenuated upon mutual adjustment, as well as upon adjustment for several other amino acids (including valine, phenylalanine, serine, and tyrosine). In analyses for total stroke (Supplemental Table 15), the association for proline attenuated with adjustment for glutamine, but the inverse association of glutamine and positive association of hydroxyproline both remained significant upon adjustment for all amino acids individually. In models further adjusting for macronutrients and food sources, the association of glutamine with both ischemic (Supplemental Table 16) and total stroke (Supplemental Table 17) remained upon adjusting for different food sources but partly attenuated upon adjustment for dairy protein in ischemic stroke. The association of proline with both ischemic and total stroke attenuated and became marginally significant upon adjustment for dairy foods, whereas the association of hydroxyproline with total stroke attenuated with adjustment for total meat or red meat, but all other associations remained.

**TABLE 2:** Hazard ratios [95% confidence intervals (CIs)] for ischemic stroke (3058 cases) by per SD difference of energy-adjusted grams of dietary amino acids significantly associated with risk, further adjusting for other dietary amino acids.

Further adjusted for	*ρ*^1^	Glutamine	*P* value^3^	*ρ*^1^	Proline	*P* value^3^
		HR (95% CI)^2^			HR (95% CI)^2^	
Multivariable		0.94 (0.90, 0.98)	0.004		0.94 (0.90, 0.98)	0.005
Further adjusted for						
Isoleucine	0.62	0.94 (0.89, 0.99)	0.03	0.86	0.91 (0.84, 0.99)	0.02
Leucine	0.64	0.94 (0.89, 1.00)	0.04	0.90	0.91 (0.83, 0.99)	0.03
Valine	0.63	0.95 (0.90, 1.00)	0.05	0.90	0.92 (0.84, 1.00)	0.06
Histidine	0.57	0.94 (0.89, 0.99)	0.02	0.79	0.91 (0.86, 0.98)	0.009
Lysine	0.53	0.94 (0.89, 0.99)	0.02	0.80	0.92 (0.86, 0.98)	0.01
Methionine	0.56	0.94 (0.89, 0.99)	0.03	0.80	0.92 (0.86, 0.99)	0.03
Phenylalanine	0.69	0.95 (0.89, 1.00)	0.06	0.88	0.92 (0.85, 1.01)	0.07
Threonine	0.57	0.94 (0.89, 0.99)	0.02	0.79	0.91 (0.85, 0.98)	0.01
Tryptophan	0.66	0.94 (0.89, 0.99)	0.03	0.82	0.91 (0.84, 0.98)	0.02
Alanine	0.53	0.94 (0.89, 0.98)	0.008	0.71	0.92 (0.87, 0.98)	0.007
Arginine	0.52	0.94 (0.89, 0.98)	0.009	0.66	0.93 (0.88, 0.98)	0.009
Asparagine	0.61	0.94 (0.89, 0.99)	0.02	0.81	0.92 (0.86, 0.98)	0.01
Aspartic acid	0.47	0.94 (0.89, 0.98)	0.006	0.66	0.93 (0.88, 0.98)	0.005
Cystine	0.69	0.93 (0.88, 0.99)	0.02	0.79	0.92 (0.87, 0.98)	0.02
Glutamine	–	–	–	0.76	0.97 (0.91, 1.03)	0.29
Glutamic acid	0.52	0.94 (0.90, 0.99)	0.02	0.82	0.93 (0.87, 0.99)	0.03
Glycine	0.50	0.93 (0.89, 0.98)	0.006	0.62	0.93 (0.88, 0.98)	0.006
Proline	0.76	0.96 (0.90, 1.03)	0.24	–	–	–
Serine	0.68	0.95 (0.89, 1.00)	0.06	0.91	0.91 (0.82, 1.00)	0.05
Tyrosine	0.63	0.95 (0.90, 1.01)	0.08	0.91	0.93 (0.84, 1.03)	0.15
Hydroxyproline	0.15	0.94 (0.90, 0.98)	0.003	0.31	0.93 (0.89, 0.97)	0.001
Taurine	0.34	0.94 (0.89, 0.98)	0.007	0.45	0.94 (0.90, 0.98)	0.009

Abbreviations: HPFS, Health Professionals Follow-Up Study; NHS, Nurses’ Health Study; NHSII, Nurses’ Health Study II.

1 Spearman’s rho for correlation between glutamine or proline and each of the other amino acids, based on dietary data collected at mid-point of follow-up (1998 for NHS, 2003 for NHSII, 1998 for HPFS).

2 Analyses were stratified by age (in years) and follow-up intervals, and adjusted for ethnicity (White, non-White), marital status (married, widowed, divorced/separated, unknown), smoking status (never, past, current: 1–14, 15–24, ≥25 cigarettes/d, unknown status or cigarette number), alcohol intake (never drinkers, 0.1–4.9, 5.0–9.9, 10.0– 14.9, 15.0+ g/d), physical activity (<3, 3–9, 9–18, 18–27, 27–42, ≥42 metabolic equivalents/week, unknown), menopausal status and postmenopausal hormone use (premenopausal, postmenopausal: never, past, current users, NHS, and NHSII only), oral contraception use (never, past, current user, NHSII only), multivitamin use (no, yes), aspirin use (no, yes), BMI (<23, 23–24.9, 25–29.9, 30–34.9, ≥35 kg/m^2^, missing), baseline history of hypertension (no, yes), baseline hypercholesteremia (no, yes), baseline diabetes (no, yes), and energy intake (fifths of intake). Results from NHS, NHSII, and HPFS were pooled using a fixed-effect meta-analysis.

3 *P* values estimated by fitting the continuous intakes of dietary amino acids in the Cox regression.

In stratified analyses, the associations for both glutamine and proline remained directionally consistent across strata of age, BMI, smoking status, alcohol consumption, physical activity, multivitamin use, and history of hypertension, high blood cholesterol or diabetes, for both ischemic (Table 3) and total stroke (Supplemental Table 18). For hydroxyproline and total stroke (Supplemental Table 18), a significant positive association was only observed among ever smokers, but not never smokers in stratified analyses (never smokers: 1.00, 0.96–1.05; ever smokers: 1.08, 1.04–1.13; *P*-interaction = 0.07).

**TABLE 3:** Hazard ratios (HR) [95% confidence intervals (CIs)] for ischemic stroke by per SD difference of energy-adjusted grams of dietary amino acids significantly associated with risk, stratified by key covariates.

	HR (95% CI)^1^	*P* value^2^	HR (95% CI)^1^	*P* value^2^	*P*-interaction^3^
Age	Age below 55 y		Age 55 y or older		
Cases/person-years	275/2,394,569		2783/2,569,715		
Glutamine	0.90 (0.79, 1.03)	0.14	0.94 (0.90, 0.99)	0.01	0.57
Proline	0.90 (0.79, 1.04)	0.15	0.95 (0.90, 0.99)	0.01	0.42
BMI	Normal weight (<25kg/m^2^)		Overweight (≥25 kg/m^2^)		
Cases/person-years	1229/2,358,393		1826/2,581,743		
Glutamine	0.94 (0.88, 1.01)	0.08	0.94 (0.89, 1.00)	0.03	0.35
Proline	0.93 (0.87, 0.99)	0.03	0.95 (0.90, 1.01)	0.08	0.17
Smoking status	Never smokers		Ever smokers		
Cases/person-years	1269/2,650,509		1742/2,253,886		
Glutamine	0.92 (0.86, 0.99)	0.02	0.94 (0.89, 1.00)	0.05	0.68
Proline	0.91 (0.85, 0.97)	0.005	0.96 (0.91, 1.01)	0.15	0.36
Alcohol consumption	Never/occasional drinkers (<5g/d)		Regular drinkers (≥5g/d)		
Cases/person-years	1746/3,283,161		1312/1,681,124		
Glutamine	0.94 (0.89, 1.00)	0.03	0.95 (0.88, 1.02)	0.13	0.98
Proline	0.94 (0.90, 1.00)	0.04	0.95 (0.88, 1.02)	0.13	0.80
Physical activity	Below median		Above median		
Cases/person-years	1526/2,275,870		1532/2,688,411		
Glutamine	0.95 (0.90, 1.01)	0.11	0.93 (0.87, 0.99)	0.02	0.55
Proline	0.95 (0.90, 1.01)	0.10	0.93 (0.88, 0.99)	0.02	0.59
Multivitamin use	No		Yes		
Cases/person-years	1277/2,280,340		1781/2,683,941		
Glutamine	0.90 (0.84, 0.96)	0.002	0.97 (0.92, 1.03)	0.335013	0.77
Proline	0.91 (0.85, 0.97)	0.003	0.97 (0.92, 1.03)	0.293879	0.71
History of hypertension, high cholesterol or diabetes	No baseline disease history		Baseline disease history		
Cases/person-years	1737/3,824,596		1321/1,139,688		
Glutamine	0.98 (0.92, 1.04)	0.52	0.90 (0.85, 0.96)	0.001	0.57
Proline	0.97 (0.91, 1.02)	0.24	0.92 (0.87, 0.98)	0.009	0.48

Abbreviations: HPFS, Health Professionals Follow-Up Study; NHS, Nurses’ Health Study; NHSII, Nurses’ Health Study II.

1 Analyses were conducted in the particular subset only, with the model stratified by age (in years) and follow-up intervals, and adjusted for ethnicity (White, non-White), marital status (married, widowed, divorced/separated, unknown), smoking status (never, past, current: 1–14, 15–24, ≥25 cigarettes/d, unknown status or cigarette number), alcohol intake (never drinkers, 0.1–4.9, 5.0–9.9, 10.0–14.9, 15.0+ g/d), physical activity (<3, 3–9, 9–18, 18–27, 27–42, ≥42 metabolic equivalents/week, unknown), menopausal status and postmenopausal hormone use (premenopausal, postmenopausal: never, past, current users, NHS, and NHSII only), oral contraception use (never, past, current user, NHSII only), multivitamin use (no, yes), aspirin use (no, yes), BMI (<23, 23–24.9, 25–29.9, 30–34.9, ≥35 kg/m^2^, missing), baseline history of hypertension (no, yes), baseline hypercholesteremia (no, yes), baseline diabetes (no, yes), and energy intake (fifths of intake). Results from NHS, NHSII, and HPFS were pooled using a fixed-effect meta-analysis.

2 *P* values estimated by fitting the continuous intakes of dietary amino acids in the Cox regression.-interaction estimated by fitting an interaction term of the stratifying variable with the exposure of interest in the Cox regression.

## Discussion

In these 3 large prospective cohort studies, higher intakes of glutamine and proline were associated with lower risks of ischemic and total stroke, whereas higher risk of hydroxyproline was associated with higher risk of total stroke. The associations for glutamine and proline, but less so for hydroxyproline, appeared consistent across sensitivity analyses, but may not be mutually independent of other amino acids or independent of their major food sources.

There is limited prior evidence on the associations of dietary amino acids and stroke types. The previous largest study was from the EPIC cohort, which included 4295 cases of ischemic stroke and 1375 cases of hemorrhagic stroke after a median follow-up of 13 y [10]. Consistent with the current study, an inverse association of proline with ischemic stroke (0.91, 0.86–0.95 per SD higher in g/d) was also observed in EPIC. Estimates for glutamine were not available in EPIC, but an inverse association was observed for glutamic acid (0.93, 0.88–0.98 per SD higher in g/d), which in the EPIC dataset is likely a composite of glutamic acid and glutamine, based on the use of the USDA database whereby glutamic acid is overestimated due to conversion from glutamine [14]. Likewise, the Takayama Study in Japan also reported an inverse association of glutamic acid intake with total stroke mortality (667 deaths) among females after 16 y of follow-up, but the study did not report on glutamine or proline [12]. Meanwhile, a study from the Swedish Mammography Cohort with 1751 cases of total stroke after 10 y of follow-up reported an inverse association between dietary cysteine and total stroke risk [11], but no associations were observed for cystine, the dimeric form of cysteine, in either the current study or the EPIC study. The Swedish study also did not have data on glutamine, but an inverse association was observed for glutamic acid but not proline before adjustment for cysteine. None of the other studies reported a significant association of hydroxyproline with stroke risk; given that the association of hydroxyproline and total stroke was only significant among ever smokers in the current analyses, this finding may be due to residual confounding, particularly when considering the very low intake of hydroxyproline in these populations.

The possible mechanisms that could link glutamine and proline with stroke risk are uncertain. The International Study of Macro-and Micro-Nutrients (INTERMAP) reported inverse associations of both dietary glutamic acid (glutamine was not available) and to a lesser degree dietary proline, with blood pressure [23]. The potential blood pressure lowering effect of glutamine is also supported by a cross-sectional investigation of circulating glutamine and blood pressure in the Framingham Heart Study and the Malmö Diet and Cancer Study, as well as in mice after intraperitoneal injection of glutamine [24]. However, analyses in the EPIC Study found little correlation of the amino acids with systolic or diastolic blood pressure, and further adjustment for blood pressure also had minimal impact on the observed associations for either glutamic acid or proline with stroke risk [10].

Future studies should aim to incorporate information from dietary databases that differentiate glutamine and glutamic acid, because circulating levels of glutamine have been observed to be associated with lower insulin resistance and triglycerides, and higher HDL cholesterol, in addition to lower blood pressure, whereas in the same study, glutamic acid was associated with cardiometabolic markers in the opposite direction [24]. Results from several small clinical studies have suggested a potential cardioprotective role of glutamine administration in patients with coronary artery disease [[25], [26], [27]], whereas some studies have reported on the role of glutamine in protecting against ischemia-reperfusion injury and apoptosis in cardiomyocytes [9], as well as promoting adipocyte differentiation and regulation of lipid metabolism [28], supporting a protective role for glutamine in multiple systems. Specifically for brain health, an experimental study in mice reported that L-glutamine administration reduced brain infarct volume and protected neuronal survival in mice after brain ischemia [29], but a study in humans has reported that higher serum glutamine may be associated with an increased risk of stroke in patients with Moyamoya disease [30]. Therefore, the potential role of glutamine in cerebrovascular function requires further investigation.

Proline, hydroxyproline, and glycine are the major amino acids in collagen, the most abundant protein in human body, which has an important role in maintaining vascular strength [31]. However, although the structural integrity of blood vessels is more intuitively important for the risk of hemorrhagic stroke, its potential relevance for ischemic stroke is less clear [32]. Other lines of evidence have suggested that glycoprolines, a family of proline-containing peptides, may exert atheroprotective or neuroprotective roles such as lowering of total cholesterol or promotion of neural plasticity [33,34]. However, human studies on circulating proline have yielded inconsistent results on their potential relevance for either stroke events or stroke recovery [[35], [36], [37], [38]], and further studies are needed.

Although no significant associations were observed for hemorrhagic stroke in this study, the number of cases was limited. Although the associations for glutamine with both stroke types appeared directionally consistent, proline appeared to be associated with ischemic stroke only, and thus the potential specificity of the associations also warrants further investigation, as they may indicate different disease mechanisms for the 2 amino acids. For example, although high blood pressure is a shared risk factor for both stroke types [39], several other risk factors, such as higher circulating LDL cholesterol [3] and obesity [4], are associated with higher risk of ischemic stroke but lower risk of hemorrhagic stroke. There has been some evidence of a positive effect of proline on bone mineral density [40] but limited evidence on other health outcomes [41], whereas higher intakes of glutamine have been associated with lower cardiovascular disease incidence and mortality among people with type 2 diabetes [42], as well as lower cardiovascular, cancer, and total mortality [43]. Furthermore, work should therefore aim to assess the potential specificity of both amino acids with a wide range of health outcomes, for example, using outcome-wide analyses [44], to assess whether the observed associations are likely to be mechanistic, or whether higher intakes of these amino acids might be a marker of a healthy lifestyle.

In terms of dietary sources, glutamine intake is correlated with plant protein and fiber, as well as whole grains, poultry, and milk, whereas proline is particularly correlated with dairy protein and dairy products, but also with poultry to a lesser degree. Therefore, the shared food sources for glutamine and proline may partly contribute to the inverse associations observed for both amino acids. The current findings are also in line with previous studies which have reported inverse associations of both plant protein and plant foods [5,6,45], as well as dietary fiber [5,46] and dairy products [5,47], with either total or ischemic stroke. Given that the inverse associations of both glutamine and proline with ischemic stroke attenuated upon adjustment for dairy protein and/or dairy foods, it is possible that these amino acids, and proline in particular, is a marker of dairy intake, and do not have an independent role in stroke risk. However, in the parallel analyses in EPIC, the association for proline remained even after adjustment for dairy foods [10]. Therefore, further investigation is needed to disentangle whether the amino acids or other components in the relevant foods may be driving the associations observed for stroke risk.

The study has several strengths, including its large sample size, the prospective design with almost 30 y of follow-up with very high response rates (≈90%), as well as repeat measures of both dietary amino acids and key covariates. In contrast to most existing studies which do not have data on glutamine and asparagine, we were able to assess the associations of all 20 standard amino acids, as well as 2 nonstandard amino acids. However, the study also had some limitations. Although we had a reasonable number of ischemic stroke cases, the number of hemorrhagic stroke cases was relatively low, especially in subset analyses, partly because not all stroke cases recorded could be classified into a subtype. All participants in this study were health professionals and were of predominantly non-Hispanic white ethnicity, and thus the generalizability of the findings may be limited. However, the results were comparable to the EPIC study, which also consisted of almost all white Europeans. Since self-reported estimates of diet were used, these are affected by measurement error, but our use of repeat measures would have reduced its impact. Although we adjusted for many key lifestyle factors, some residual confounding is expected, but should not have a major effect on results.

In conclusion, in these analyses using data from 3 large prospective cohort studies, we found that higher intakes of glutamine and proline were associated with lower risks of ischemic and total stroke, although these associations may not be mutually independent or independent of their major food sources. Further work is needed to understand the potential mechanisms linking these amino acids to stroke risk, such as outcome-wide analyses to establish the potential specificity of the associations, and intervention trials to assess their effects on specific disease risk factors.

## Author contributions

The authors’ responsibilities were as follows – TYNT, FBH: full access to all of the data in the study and responsibility for the integrity of the data and the accuracy of the data analysis; TYNT, TJK, FBH: conceived of and designed the research question; TYNT: analyzed the data and wrote the first draft of the manuscript; YL, KMR, WCW, QS, JEM, VDL, TJK, FBH: acquisition or interpretation of data; and all authors: read and approved the final manuscript.

## Data availability

Because of participant confidentiality and privacy concerns, data cannot be shared publicly, and requests to access Nurses’ Health Studies/Health Professionals Follow-up Study data must be submitted in writing. Further information including the procedures to obtain and access data from the Nurses’ Health Studies and Health Professionals’ Follow-up Study is described at https://www.nurseshealthstudy.org/researchers (contact e-mail: nhsaccess@channing.harvard.edu) and https://sites.sph.harvard.edu/hpfs/for-collaborators/.

## Funding

TYNT is supported by a UK Research and Innovation Future Leaders Fellowship (MR/X032809/1). The Nurses’ Health Studies and Health Professional Follow-Up Studies are supported by National Institutes of Health grants (UM1 CA186107, R01 CA49449, R01 HL034594, U01 HL145386, R01 HL088521, U01 CA176726, R01 CA49449, U01 CA167552, R01 HL60712, and R01 HL35464). The content is solely the responsibility of the authors and does not necessarily represent the official views of the National Institutes of Health. The funders had no role in the design of the study; in the collection, analyses, or interpretation of data; in the writing of the manuscript; or in the decision to publish the results. For the purpose of open access, the authors have applied a Creative Commons Attribution (CC BY) license to any Author Accepted Manuscript version arising.

## Conflict of interest

VDL has equity interest in L-Nutra, which develops and sells medical food for the prevention and treatment of diseases. All the other authors report no conflicts of interest.
