# Supplementary material for: Dietary Amino Acids and Risk of Stroke Subtypes: Results from 3 Large Prospective Cohort Studies
Source: J Nutr. 2025 Mar 22;155(5):1560–9. doi: 10.1016/j.tjnut.2025.03.026 (PMC12121403; doi:10.1016/j.tjnut.2025.03.026)
Supplement: multimedia component 1 [file mmc1.docx]

Table of Contents

[Supplementary figure 1: Participant flow chart of the study 3](#_Toc191917567)

[Supplementary table 1: Intakes of dietary amino acids in NHS, NHSII and HPFS at baseline. 4](#_Toc191917568)

[Supplementary table 2: Baseline characteristics of participants in the Nurses’ Health Study (NHS), Nurses’ Health Study II (NHSII) and Health Professionals Follow-Up Study (HPFS) by fifths of glutamine intake. 5](#_Toc191917569)

[Supplementary table 3: Baseline characteristics of participants in the Nurses’ Health Study (NHS), Nurses’ Health Study II (NHSII) and Health Professionals Follow-Up Study (HPFS) by fifths of proline intake. 8](#_Toc191917570)

[Supplementary table 4: Baseline characteristics of participants in the Nurses’ Health Study (NHS), Nurses’ Health Study II (NHSII) and Health Professionals Follow-Up Study (HPFS) by fifths of hydroxyproline intake. 11](#_Toc191917571)

[Supplementary figure 2: Correlation matrix between energy-adjusted grams of dietary amino acids in NHS, NHSII and HPFS, based on dietary data collected at mid-point of follow-up (1998 for NHS, 2003 for NHSII, 1998 for HPFS). 14](#_Toc191917572)

[Supplementary figure 3: Correlation matrix between energy-adjusted grams of dietary amino acids and macronutrients and major food groups in NHS, NHSII and HPFS, based on dietary data collected at mid-point of follow-up (1998 for NHS, 2003 for NHSII, 1998 for HPFS). 15](#_Toc191917573)

[Supplementary table 5: Hazard ratios (95% confidence intervals) for ischemic stroke (3058 cases) by fifths and per SD difference of energy-adjusted grams of dietary amino acids. 16](#_Toc191917574)

[Supplementary table 6: Hazard ratios (95% confidence intervals) for hemorrhagic stroke (872 cases) by fifths and per SD difference of energy-adjusted grams of dietary amino acids. 21](#_Toc191917575)

[Supplementary table 7: Hazard ratios (95% confidence intervals) for total stroke (5997 cases) by fifths and per SD difference of energy-adjusted grams of dietary amino acids. 26](#_Toc191917576)

[Supplementary table 8: Hazard ratios (95% confidence intervals) for ischemic stroke (3058 cases) by fifths and per SD difference of energy-adjusted grams of dietary amino acids, across different adjustment models. 31](#_Toc191917577)

[Supplementary table 9: Hazard ratios (95% confidence intervals) for hemorrhagic stroke (872 cases) by fifths and per SD difference of energy-adjusted grams of dietary amino acids, across different adjustment models. 35](#_Toc191917578)

[Supplementary table 10: Hazard ratios (95% confidence intervals) for total stroke (5997 cases) by fifths and per SD difference of energy-adjusted grams of dietary amino acids, across different adjustment models. 39](#_Toc191917579)

[Supplementary table 11: Hazard ratios (95% confidence intervals) for ischemic stroke (3058 cases) by fifths and per SD difference of raw grams of dietary amino acids. 43](#_Toc191917580)

[Supplementary table 12: Hazard ratios (95% confidence intervals) for hemorrhagic stroke (872 cases) by fifths and per SD difference of raw grams of dietary amino acids. 45](#_Toc191917581)

[Supplementary table 13: Hazard ratios (95% confidence intervals) for total stroke (5997 cases) by fifths and per SD difference of raw grams of dietary amino acids. 47](#_Toc191917582)

[Supplementary table 14: Hazard ratios (95% confidence intervals) for ischemic, hemorrhagic and total stroke by per SD difference of energy-adjusted grams of dietary amino acids, with a 4-year lag. 49](#_Toc191917583)

[Supplementary table 15: Hazard ratios (95% confidence intervals) for total stroke (5997 cases) by per SD difference of energy-adjusted grams of dietary amino acids significantly associated with risk, further adjusting for other dietary amino acids. 51](#_Toc191917584)

[Supplementary table 16: Hazard ratios (95% confidence intervals) for ischemic stroke (3058 cases) by per SD difference of energy-adjusted grams of dietary amino acids significantly associated with risk, further adjusting for macronutrients and major dietary sources. 53](#_Toc191917585)

[Supplementary table 17: Hazard ratios (95% confidence intervals) for total stroke (5997 cases) by per SD difference of energy-adjusted grams of dietary amino acids significantly associated with risk, further adjusting for macronutrients and major dietary sources. 55](#_Toc191917586)

[Supplementary table 18: Hazard ratios (95% confidence intervals) for total stroke by per SD difference of energy-adjusted grams of dietary amino acids significantly associated with risk, stratified by key covariates. 57](#_Toc191917587)

NHS (n=121,701)
NHSII (n=116,429)
HPFS (n=51,529)

Exclusions based on the following reasons:

- With missing age (n=172 in NHS, n=21 in NHSII, n=37 in HPFS)
- Only returned the baseline questionnaire (n=987 in NHS, n=960 in NHSII, n=1118 in HPFS)
- Baseline history of stroke (n=750 in NHS, n=385 in NHSII, n=406 in HPFS)
- Death before baseline (n=1800 in NHS, n=42 in NHSII, n=3 in HPFS)
- Baseline history of cancer (n=6018 in NHS, n=1567 in NHSII, n=1880 in HPFS)
- Baseline history of myocardial infarction, angina or coronary artery bypass grafting (n=3695 in NHS, n=787 in NHSII, n=3497 in HPFS)
- Missing dietary data or implausible energy intakes defined as <500 kcal/d or >3500 kcal/d in women or <800 kcal/d or >4200 kcal/d in men (n=34,449 in NHS, n=20,334 in NHSII, n=1320 in HPFS)

NHS (n=73,830)
NHSII (n=92,333)
HPFS (n=43,268)

Supplementary figure 1: Participant flow chart of the study

Supplementary table 1: Intakes of dietary amino acids in NHS, NHSII and HPFS at baseline.

|  | **NHS (1984, n=73,830)** | | **NHSII (1991, n=92,333)** | | **HPFS (1986, n=43,268)** | |
| --- | --- | --- | --- | --- | --- | --- |
|  | Adjusted g/day ^1^ | Unadjusted g/day ^2^ | Adjusted g/day ^1^ | Unadjusted g/day ^2^ | Adjusted g/day ^1^ | Unadjusted g/day ^2^ |
| Branched-chain amino acids |  |  |  |  |  |  |
| Isoleucine | 3.3 (0.7) | 3.5 (1.2) | 4.1 (0.8) | 4.0 (1.4) | 4.3 (0.8) | 4.2 (1.5) |
| Leucine | 5.5 (1.1) | 5.9 (1.9) | 6.7 (1.2) | 6.7 (2.2) | 7.1 (1.3) | 7.0 (2.4) |
| Valine | 3.6 (0.7) | 3.9 (1.3) | 4.5 (0.8) | 4.4 (1.5) | 4.7 (0.9) | 4.7 (1.6) |
| Other essential amino acids |  |  |  |  |  |  |
| Histidine | 2.0 (0.4) | 2.2 (0.7) | 2.5 (0.5) | 2.4 (0.8) | 2.6 (0.5) | 2.6 (0.9) |
| Lysine | 5.1 (1.1) | 5.4 (1.8) | 6.2 (1.4) | 6.1 (2.1) | 6.5 (1.4) | 6.5 (2.3) |
| Methionine | 1.7 (0.4) | 1.8 (0.6) | 2.0 (0.4) | 2.0 (0.7) | 2.2 (0.5) | 2.2 (0.8) |
| Phenylalanine | 3.0 (0.5) | 3.2 (1.0) | 3.7 (0.6) | 3.7 (1.2) | 3.9 (0.7) | 3.9 (1.3) |
| Threonine | 2.8 (0.6) | 3.0 (1.0) | 3.4 (0.7) | 3.4 (1.1) | 3.6 (0.7) | 3.6 (1.2) |
| Tryptophan | 0.8 (0.2) | 0.9 (0.3) | 1.0 (0.2) | 1.0 (0.3) | 1.1 (0.2) | 1.1 (0.4) |
| Non-essential amino acids |  |  |  |  |  |  |
| Alanine | 3.5 (0.7) | 3.7 (1.2) | 4.1 (0.9) | 4.1 (1.4) | 4.5 (0.9) | 4.5 (1.5) |
| Arginine | 4.1 (0.9) | 4.4 (1.4) | 4.7 (0.9) | 4.7 (1.6) | 5.1 (1.0) | 5.1 (1.8) |
| Asparagine | 2.6 (0.5) | 2.8 (0.9) | 2.9 (0.5) | 2.9 (1.0) | 3.2 (0.6) | 3.1 (1.0) |
| Aspartic acid | 3.4 (0.8) | 3.6 (1.2) | 3.9 (0.9) | 3.8 (1.3) | 4.2 (0.9) | 4.1 (1.4) |
| Cystine | 0.9 (0.2) | 0.9 (0.3) | 1.1 (0.2) | 1.1 (0.3) | 1.1 (0.2) | 1.1 (0.4) |
| Glutamine | 6.4 (1.0) | 6.9 (2.2) | 7.8 (1.2) | 7.7 (2.5) | 8.0 (1.3) | 7.9 (2.6) |
| Glutamic acid | 6.8 (1.6) | 7.3 (2.5) | 7.8 (1.6) | 7.7 (2.7) | 8.3 (1.7) | 8.3 (2.9) |
| Glycine | 3.1 (0.7) | 3.3 (1.1) | 3.6 (0.8) | 3.5 (1.2) | 4.0 (0.9) | 4.0 (1.4) |
| Proline | 4.2 (0.8) | 4.6 (1.5) | 5.3 (0.9) | 5.2 (1.7) | 5.4 (0.9) | 5.4 (1.8) |
| Serine | 3.1 (0.5) | 3.3 (1.1) | 3.8 (0.6) | 3.7 (1.2) | 3.9 (0.7) | 3.9 (1.3) |
| Tyrosine | 2.5 (0.5) | 2.7 (0.9) | 3.1 (0.6) | 3.1 (1.0) | 3.2 (0.6) | 3.2 (1.1) |
| Non-standard amino acids |  |  |  |  |  |  |
| Hydroxyproline | 0.3 (0.1) | 0.4 (0.2) | 0.3 (0.1) | 0.3 (0.1) | 0.4 (0.1) | 0.4 (0.2) |
| Taurine | 0.1 (0.1) | 0.1 (0.1) | 0.2 (0.1) | 0.2 (0.1) | 0.2 (0.1) | 0.2 (0.1) |

All estimates are mean (SD) of intake.
1. Adjusted for total energy using the residuals method.
2. Not adjusted for total energy.

Supplementary table 2: Baseline characteristics of participants in the Nurses’ Health Study (NHS), Nurses’ Health Study II (NHSII) and Health Professionals Follow-Up Study (HPFS) by fifths of glutamine intake.

|  | **Fifths of glutamine intake^2^** | | | | |
| --- | --- | --- | --- | --- | --- |
| **Cohort and baseline characteristics^1^** | **Q1** | **Q2** | **Q3** | **Q4** | **Q5** |
| **NHS (1984)** |  |  |  |  |  |
| Number of participants | 14,853 | 14,538 | 14,934 | 14,736 | 14,769 |
| Glutamine intake, g/day^2^ | 5.0 (0.5) | 5.9 (0.2) | 6.4 (0.1) | 6.9 (0.2) | 7.8 (0.6) |
| Age at baseline, years | 50.4 (7.2) | 50 (7.1) | 49.9 (7.1) | 50.1 (7.1) | 50.5 (7.2) |
| White ethnicity, % | 96.2 | 97.8 | 98.1 | 98.4 | 98.4 |
| Currently married, % | 80.8 | 82.8 | 82.7 | 82.7 | 82.0 |
| Body mass index, kg/m^2^ | 24.4 (4.6) | 24.8 (4.6) | 25 (4.6) | 25.1 (4.6) | 25.6 (4.9) |
| Physical activity, MET-hrs/week | 12.5 (19.5) | 13.8 (21.2) | 14.1 (22.4) | 14.2 (18.8) | 16.1 (22.9) |
| Smoking status, % |  |  |  |  |  |
| Never smoker | 39.2 | 44.1 | 45.2 | 45.4 | 46.0 |
| Past smoker | 27.7 | 29.9 | 32.0 | 33.3 | 35.7 |
| Current smoker | 33.0 | 25.9 | 22.7 | 21.1 | 18.1 |
| Alcohol consumption, g/day | 11.5 (16.6) | 7.8 (11.5) | 6.3 (9.4) | 5.2 (7.8) | 3.8 (6.1) |
| Current postmenopausal hormone use, % | 13.4 | 14.1 | 13.5 | 13.5 | 13.7 |
| Aspirin use, % | 69.7 | 71.6 | 71.9 | 72.0 | 70.2 |
| Multivitamin use, % | 33.0 | 35.1 | 36.8 | 38.2 | 41.6 |
| Baseline diabetes, % | 1.5 | 2.1 | 2.7 | 3.0 | 4.9 |
| Baseline hypertension, % | 21.2 | 20.2 | 20.6 | 20.5 | 22.2 |
| Baseline high blood cholesterol, % | 7.1 | 7.0 | 7.8 | 8.0 | 9.8 |
| Energy intake, kcal/day | 1729.3 (555.6) | 1752.2 (534.4) | 1759.3 (527.6) | 1768.7 (517.4) | 1716 (515.5) |
| Total protein, g/day^2^ | 59.7 (9.6) | 67.4 (8.9) | 71.6 (9.4) | 75.5 (10) | 83.1 (13.5) |
| Animal protein, g/day^2^ | 42.9 (10.5) | 49.1 (10.6) | 52.5 (11.4) | 55.7 (12.2) | 61.9 (16) |
| Plant protein, g/day^2^ | 16.8 (4.2) | 18.3 (3.7) | 19.1 (3.8) | 19.8 (3.8) | 21.2 (4.7) |
| Dairy protein, g/day^2^ | 10.1 (5.2) | 12.4 (5.8) | 14.1 (6.6) | 15.8 (7.4) | 18.6 (9.1) |
| Saturated fat, g/day^2^ | 22 (5.1) | 22.9 (4.5) | 22.7 (4.4) | 22.3 (4.3) | 21 (4.2) |
| Monounsaturated fat, g/day^2^ | 22.5 (4.8) | 23.4 (4.2) | 23.1 (4.1) | 22.5 (3.9) | 20.8 (4) |
| Polyunsaturated fat, g/day^2^ | 11.9 (3.8) | 12.1 (3.1) | 12 (2.9) | 11.7 (2.8) | 11.2 (2.8) |
| Total carbohydrates, g/day^2^ | 189.4 (38.8) | 183.3 (30.6) | 183 (29.1) | 183.8 (28.2) | 186.2 (28.5) |
| Total fibre, g/day^2^ | 14.5 (4.8) | 15.7 (4.4) | 16.4 (4.5) | 17 (4.6) | 18.2 (5.1) |
|  | **Fifths of glutamine intake^2^** | | | | |
|  | **Q1** | **Q2** | **Q3** | **Q4** | **Q5** |
| **NHSII (1991)** |  |  |  |  |  |
| Number of participants | 18,550 | 18,217 | 18,604 | 18,516 | 18,446 |
| Glutamine intake, g/day^2^ | 6.1 (0.6) | 7.2 (0.2) | 7.7 (0.2) | 8.4 (0.2) | 9.4 (0.7) |
| Age at baseline, years | 36.1 (4.8) | 36.2 (4.7) | 36.1 (4.7) | 36.1 (4.6) | 36.1 (4.5) |
| White ethnicity, % | 93.1 | 96.0 | 97.3 | 97.7 | 98.2 |
| Currently married, % | 87.4 | 89.6 | 90.4 | 90.9 | 89.9 |
| Body mass index, kg/m^2^ | 24.5 (5.6) | 24.6 (5.3) | 24.7 (5.3) | 24.7 (5.2) | 24.7 (5.2) |
| Physical activity, MET-hrs/week | 18.7 (26.5) | 19.9 (25.9) | 20.6 (26.2) | 21.6 (27.1) | 23.2 (29.5) |
| Smoking status, % |  |  |  |  |  |
| Never smoker | 62.6 | 64.9 | 66.2 | 66.8 | 67.2 |
| Past smoker | 19.0 | 21.5 | 22.6 | 23.6 | 24.5 |
| Current smoker | 18.4 | 13.5 | 11.1 | 9.5 | 8.2 |
| Alcohol consumption, g/day | 3.9 (8.5) | 3.5 (6.4) | 3.1 (5.4) | 2.8 (4.8) | 2.3 (4.1) |
| Current postmenopausal hormone use, % | 2.9 | 2.7 | 2.6 | 2.4 | 2.3 |
| Current oral contraceptive use, % | 14.8 | 15.2 | 14.8 | 15.5 | 16.7 |
| Aspirin use, % | 12.2 | 11.2 | 10.8 | 11.1 | 10.6 |
| Multivitamin use, % | 39.6 | 42.9 | 44.5 | 45.3 | 47.0 |
| Baseline diabetes, % | 0.7 | 0.7 | 1.0 | 1.1 | 1.4 |
| Baseline hypertension, % | 7.0 | 6.3 | 6.2 | 5.9 | 6.0 |
| Baseline high blood cholesterol, % | 15.2 | 14.0 | 14.6 | 14.5 | 14.4 |
| Energy intake, kcal/day | 1799.4 (588.5) | 1782.9 (557.5) | 1794.6 (537.5) | 1796.4 (532.2) | 1774.4 (519.8) |
| Total protein, g/day^2^ | 72.4 (11.8) | 82.5 (10.9) | 87.6 (11.6) | 92.1 (12.7) | 97.9 (15.8) |
| Animal protein, g/day^2^ | 52.6 (13.1) | 61 (13.3) | 65.2 (14.1) | 68.8 (15.3) | 72.8 (18.7) |
| Plant protein, g/day^2^ | 19.8 (4.9) | 21.6 (4.4) | 22.5 (4.5) | 23.3 (4.6) | 25.2 (5.1) |
| Dairy protein, g/day^2^ | 13.6 (6.7) | 16.9 (7.6) | 18.9 (8.3) | 20.8 (9.2) | 22.8 (10.1) |
| Saturated fat, g/day^2^ | 23.1 (5.7) | 23.4 (4.8) | 22.9 (4.5) | 22.1 (4.4) | 20.7 (4.3) |
| Monounsaturated fat, g/day^2^ | 24.9 (5.5) | 25.3 (4.8) | 24.6 (4.5) | 23.5 (4.3) | 21.5 (4.2) |
| Polyunsaturated fat, g/day^2^ | 11.2 (3.3) | 11.6 (2.8) | 11.5 (2.6) | 11.2 (2.5) | 10.8 (2.4) |
| Total carbohydrates, g/day^2^ | 234.6 (39.6) | 222 (33.1) | 220.4 (31.4) | 221.2 (30.8) | 225.1 (31.4) |
| Total fibre, g/day^2^ | 16.2 (5.7) | 17.9 (5.3) | 18.5 (5.2) | 19 (5.2) | 19.9 (5.5) |
|  |  |  |  |  |  |
|  | **Fifths of glutamine intake^2^** | | | | |
|  | **Q1** | **Q2** | **Q3** | **Q4** | **Q5** |
| **HPFS (1986)** |  |  |  |  |  |
| Number of participants | 8,639 | 8,649 | 8,650 | 8,672 | 8,658 |
| Glutamine intake, g/day^2^ | 6.3 (0.6) | 7.3 (0.2) | 7.9 (0.2) | 8.6 (0.2) | 9.8 (0.9) |
| Age at baseline, years | 53.6 (9.7) | 53.1 (9.5) | 52.9 (9.4) | 53 (9.5) | 53.2 (9.4) |
| White ethnicity, % | 92.8 | 94.6 | 95.4 | 95.9 | 96.3 |
| Currently married, % | 91.7 | 93.0 | 93.5 | 93.4 | 93.1 |
| Body mass index, kg/m^2^ | 25.5 (3.2) | 25.6 (3.4) | 25.6 (3.3) | 25.5 (3.3) | 25.3 (3.3) |
| Physical activity, MET-hrs/week | 18.9 (24.7) | 19.6 (23.7) | 20.7 (24.5) | 21.3 (24.2) | 23.3 (27.1) |
| Smoking status, % |  |  |  |  |  |
| Never smoker | 40.3 | 44.3 | 47.1 | 48.3 | 50.6 |
| Past smoker | 43.2 | 43.4 | 42.0 | 42.4 | 41.3 |
| Current smoker | 14.3 | 10.0 | 8.4 | 7.1 | 5.5 |
| Alcohol consumption, g/day | 17.9 (21.7) | 12.9 (15.7) | 10.8 (13.5) | 8.8 (11.3) | 6.5 (9) |
| Aspirin use, % | 26.5 | 27.5 | 26.4 | 26.8 | 26.4 |
| Multivitamin use, % | 38.4 | 39.2 | 41.5 | 43.2 | 46.4 |
| Baseline diabetes, % | 1.6 | 1.9 | 1.9 | 2.7 | 4.3 |
| Baseline hypertension, % | 20.9 | 20.4 | 19.5 | 19.1 | 19.8 |
| Baseline high blood cholesterol, % | 9.5 | 9.4 | 9.8 | 11.3 | 12.4 |
| Energy intake, kcal/day | 1979.8 (645.8) | 1999.1 (623.3) | 2008.8 (608.3) | 2012.4 (619.7) | 1973.3 (607.1) |
| Total protein, g/day^2^ | 78.1 (12.5) | 87.9 (11.9) | 93 (12.9) | 97.8 (13.9) | 104.2 (17.6) |
| Animal protein, g/day^2^ | 56.2 (14) | 64.5 (14) | 68.6 (15.3) | 72.1 (16.5) | 76.1 (20.6) |
| Plant protein, g/day^2^ | 21.9 (5.9) | 23.4 (5.3) | 24.5 (5.3) | 25.6 (5.4) | 28.1 (6.9) |
| Dairy protein, g/day^2^ | 11.4 (6.5) | 14.1 (7.5) | 15.7 (8.3) | 17.6 (9.2) | 19.9 (10.8) |
| Saturated fat, g/day^2^ | 25.3 (6.6) | 25.8 (5.9) | 25.2 (5.8) | 24.4 (5.8) | 22.5 (5.9) |
| Monounsaturated fat, g/day^2^ | 28.2 (6.6) | 28.7 (5.8) | 28.1 (5.6) | 27.1 (5.5) | 24.8 (5.6) |
| Polyunsaturated fat, g/day^2^ | 13.2 (4.2) | 13.5 (3.5) | 13.4 (3.3) | 13.2 (3.1) | 12.8 (3.2) |
| Total carbohydrates, g/day^2^ | 233 (50.4) | 229 (40.6) | 231 (39) | 234.5 (38.7) | 243.2 (40.4) |
| Total fibre, g/day^2^ | 18.5 (7.1) | 19.7 (6.2) | 20.8 (6.4) | 21.8 (6.4) | 23.6 (7.6) |

1. Values are means (SD) for continuous variables and percentages for categorical variables, and standardised the age distribution of the study population.
2. Adjusted for total energy using the residual method.

Supplementary table 3: Baseline characteristics of participants in the Nurses’ Health Study (NHS), Nurses’ Health Study II (NHSII) and Health Professionals Follow-Up Study (HPFS) by fifths of proline intake.

|  | **Fifths of proline intake^2^** | | | | |
| --- | --- | --- | --- | --- | --- |
| **Cohort and baseline characteristics^1^** | **Q1** | **Q2** | **Q3** | **Q4** | **Q5** |
| **NHS (1984)** |  |  |  |  |  |
| Number of participants | 14,679 | 14,700 | 14,768 | 14,880 | 14,803 |
| Proline intake, g/day^2^ | 3.3 (0.3) | 3.8 (0.1) | 4.2 (0.1) | 4.6 (0.1) | 5.3 (0.5) |
| Age at baseline, years | 50.2 (7.2) | 50 (7.1) | 49.9 (7.1) | 50 (7.1) | 50.8 (7.1) |
| White ethnicity, % | 96.4 | 97.7 | 98.1 | 98.3 | 98.4 |
| Currently married, % | 81.4 | 82.5 | 83.3 | 82.9 | 81.0 |
| Body mass index, kg/m^2^ | 24.2 (4.5) | 24.7 (4.5) | 24.9 (4.6) | 25.4 (4.8) | 25.7 (4.9) |
| Physical activity, MET-hrs/week | 12.9 (21.2) | 13.5 (19.8) | 14 (18.9) | 14.8 (23) | 15.6 (22.1) |
| Smoking status, % |  |  |  |  |  |
| Never smoker | 39.8 | 44.2 | 45.1 | 45.6 | 45.2 |
| Past smoker | 28.0 | 30.8 | 31.9 | 33.7 | 34.2 |
| Current smoker | 32.0 | 24.9 | 22.9 | 20.6 | 20.5 |
| Alcohol consumption, g/day | 11 (16.4) | 8 (11.6) | 6.5 (9.6) | 5.2 (8) | 3.9 (6.5) |
| Current postmenopausal hormone use, % | 12.8 | 13.7 | 13.7 | 13.9 | 14.1 |
| Aspirin use, % | 69.5 | 71.6 | 72.2 | 71.2 | 70.8 |
| Multivitamin use, % | 32.9 | 34.0 | 37.0 | 38.1 | 42.6 |
| Baseline diabetes, % | 1.4 | 1.9 | 2.5 | 3.4 | 4.9 |
| Baseline hypertension, % | 21.0 | 19.8 | 20.4 | 21.1 | 22.5 |
| Baseline high blood cholesterol, % | 7.6 | 7.5 | 7.7 | 8.2 | 8.8 |
| Energy intake, kcal/day | 1735.2 (561.5) | 1755.6 (533.4) | 1753.3 (527.4) | 1758.8 (520.8) | 1721.2 (508) |
| Total protein, g/day^2^ | 56.1 (7.2) | 65.7 (5.9) | 71.2 (6.4) | 76.9 (7.3) | 87.1 (11.3) |
| Animal protein, g/day^2^ | 37.6 (8.1) | 46.3 (7.4) | 51.7 (7.8) | 57.5 (8.7) | 68.6 (12.7) |
| Plant protein, g/day^2^ | 18.5 (4.7) | 19.4 (4.1) | 19.5 (4) | 19.3 (4.2) | 18.5 (4.4) |
| Dairy protein, g/day^2^ | 8.1 (3.4) | 10.7 (4) | 13 (4.5) | 15.9 (5.4) | 23.3 (8.4) |
| Saturated fat, g/day^2^ | 20.4 (4.7) | 21.9 (4.2) | 22.5 (4.2) | 22.9 (4.4) | 23 (4.9) |
| Monounsaturated fat, g/day^2^ | 21.5 (4.4) | 22.9 (4.1) | 23 (4.1) | 22.9 (4.2) | 21.9 (4.5) |
| Polyunsaturated fat, g/day^2^ | 12.2 (3.7) | 12.3 (3.1) | 12 (2.9) | 11.6 (2.8) | 10.8 (2.8) |
| Total carbohydrates, g/day^2^ | 199.5 (35.7) | 187.7 (29) | 183.7 (28) | 179.7 (28.2) | 175.3 (30) |
| Total fibre, g/day^2^ | 15.4 (5.2) | 16.3 (4.6) | 16.6 (4.6) | 16.8 (4.7) | 16.7 (5) |
|  | **Fifths of proline intake^2^** | | | | |
|  | **Q1** | **Q2** | **Q3** | **Q4** | **Q5** |
| **NHSII (1991)** |  |  |  |  |  |
| Number of participants | 18,478 | 18,530 | 18,597 | 18,318 | 18,410 |
| Proline intake, g/day^2^ | 4.1 (0.4) | 4.8 (0.1) | 5.3 (0.1) | 5.7 (0.1) | 6.5 (0.5) |
| Age at baseline, years | 36.3 (4.7) | 36.3 (4.6) | 36.1 (4.7) | 36 (4.6) | 35.8 (4.6) |
| White ethnicity, % | 93.3 | 96.2 | 97.1 | 97.6 | 98.0 |
| Currently married, % | 87.1 | 89.7 | 90.2 | 90.7 | 90.4 |
| Body mass index, kg/m^2^ | 24 (5.3) | 24.3 (5.2) | 24.7 (5.4) | 24.9 (5.3) | 25.2 (5.4) |
| Physical activity, MET-hrs/week | 19.7 (27.4) | 20.4 (27.3) | 20.5 (26.3) | 21.3 (26.7) | 22.2 (27.9) |
| Smoking status, % |  |  |  |  |  |
| Never smoker | 63.2 | 65.4 | 65.8 | 66.0 | 67.3 |
| Past smoker | 20.1 | 21.7 | 22.6 | 23.6 | 23.1 |
| Current smoker | 16.6 | 12.8 | 11.5 | 10.3 | 9.5 |
| Alcohol consumption, g/day | 4 (8.5) | 3.6 (6.5) | 3.1 (5.5) | 2.7 (4.8) | 2 (3.8) |
| Current postmenopausal hormone use, % | 2.8 | 2.6 | 2.6 | 2.2 | 2.6 |
| Current oral contraceptive use, % | 15.6 | 15.1 | 15.2 | 14.9 | 16.2 |
| Aspirin use, % | 11.8 | 11.5 | 11.2 | 10.9 | 10.6 |
| Multivitamin use, % | 40.1 | 41.9 | 43.4 | 45.3 | 48.6 |
| Baseline diabetes, % | 0.5 | 0.8 | 0.9 | 1.1 | 1.5 |
| Baseline hypertension, % | 6.4 | 6.0 | 6.2 | 6.2 | 6.6 |
| Baseline high blood cholesterol, % | 14.9 | 14.5 | 14.3 | 14.4 | 14.7 |
| Energy intake, kcal/day | 1800.7 (587.7) | 1782.8 (556.7) | 1796.1 (543.5) | 1800.8 (537.3) | 1767 (509.7) |
| Total protein, g/day^2^ | 68.3 (9.5) | 80.2 (7.6) | 87 (8.3) | 93.4 (9.3) | 103.9 (12.9) |
| Animal protein, g/day^2^ | 46.1 (11.4) | 57.2 (9.5) | 64.1 (9.8) | 70.8 (10.6) | 82.4 (14.2) |
| Plant protein, g/day^2^ | 22.2 (6.2) | 23 (4.9) | 23 (4.6) | 22.6 (4.5) | 21.5 (4.5) |
| Dairy protein, g/day^2^ | 11 (4.6) | 14.6 (5.2) | 17.4 (5.9) | 21.1 (6.9) | 28.8 (9.8) |
| Saturated fat, g/day^2^ | 21.2 (5.4) | 22.5 (4.7) | 22.7 (4.6) | 22.8 (4.6) | 22.9 (4.7) |
| Monounsaturated fat, g/day^2^ | 24.1 (5.5) | 24.9 (4.8) | 24.5 (4.7) | 23.8 (4.6) | 22.5 (4.4) |
| Polyunsaturated fat, g/day^2^ | 11.5 (3.4) | 11.7 (2.7) | 11.5 (2.6) | 11.2 (2.4) | 10.5 (2.4) |
| Total carbohydrates, g/day^2^ | 245.6 (37.3) | 227.4 (30.7) | 221.3 (30.1) | 217.4 (29.9) | 211.7 (30.1) |
| Total fibre, g/day^2^ | 17.7 (6.4) | 18.5 (5.3) | 18.7 (5.1) | 18.6 (5.2) | 18 (5.3) |
|  |  |  |  |  |  |
|  | **Fifths of proline intake^2^** | | | | |
|  | **Q1** | **Q2** | **Q3** | **Q4** | **Q5** |
| **HPFS (1986)** |  |  |  |  |  |
| Number of participants | 8,597 | 8,728 | 8,689 | 8,609 | 8,645 |
| Proline intake, g/day^2^ | 4.2 (0.4) | 4.9 (0.1) | 5.3 (0.1) | 5.8 (0.2) | 6.7 (0.6) |
| Age at baseline, years | 53.4 (9.7) | 52.9 (9.4) | 52.9 (9.5) | 53 (9.4) | 53.7 (9.5) |
| White ethnicity, % | 92.6 | 94.7 | 95.5 | 96.0 | 96.0 |
| Currently married, % | 91.7 | 93.2 | 93.6 | 93.2 | 93.1 |
| Body mass index, kg/m^2^ | 25.1 (3.2) | 25.4 (3.2) | 25.5 (3.1) | 25.6 (3.3) | 25.9 (3.6) |
| Physical activity, MET-hrs/week | 20.3 (26.2) | 20.7 (24.4) | 20.6 (24) | 21.4 (24.8) | 21.0 (25.1) |
| Smoking status, % |  |  |  |  |  |
| Never smoker | 42.1 | 45.4 | 47.1 | 47.7 | 48.3 |
| Past smoker | 43.2 | 42.8 | 42.4 | 41.9 | 42.1 |
| Current smoker | 12.5 | 9.3 | 8.4 | 8.0 | 7.1 |
| Alcohol consumption, g/day | 18.1 (21.9) | 13.2 (15.8) | 10.5 (13) | 8.5 (10.9) | 6.5 (9.3) |
| Aspirin use, % | 26.3 | 26.3 | 26.7 | 27.0 | 27.3 |
| Multivitamin use, % | 39.4 | 40.5 | 41.1 | 42.7 | 45.1 |
| Baseline diabetes, % | 1.4 | 1.3 | 1.9 | 2.8 | 5.0 |
| Baseline hypertension, % | 20.5 | 19.1 | 19.3 | 19.6 | 21.2 |
| Baseline high blood cholesterol, % | 10.3 | 10.0 | 10.6 | 10.5 | 11.1 |
| Energy intake, kcal/day | 1997.3 (646.2) | 1997.5 (615) | 1991.4 (607.1) | 1999.9 (617.6) | 1987.3 (619.5) |
| Total protein, g/day^2^ | 73.2 (9.7) | 85.2 (7.7) | 92.2 (8.6) | 99.4 (9.9) | 111 (14.8) |
| Animal protein, g/day^2^ | 49 (11.2) | 60.1 (9.8) | 67.1 (10.4) | 74.2 (11.6) | 87.1 (16.4) |
| Plant protein, g/day^2^ | 24.3 (7.1) | 25.1 (5.9) | 25.1 (5.7) | 25.1 (5.8) | 23.9 (6) |
| Dairy protein, g/day^2^ | 9.1 (4.3) | 12 (5) | 14.4 (5.9) | 17.6 (7.1) | 25.7 (11) |
| Saturated fat, g/day^2^ | 22.8 (6.1) | 24.4 (5.7) | 25 (5.8) | 25.2 (6) | 25.9 (6.6) |
| Monounsaturated fat, g/day^2^ | 26.7 (6.6) | 27.9 (5.9) | 28 (5.8) | 27.6 (5.8) | 26.7 (5.9) |
| Polyunsaturated fat, g/day^2^ | 13.3 (4.2) | 13.6 (3.5) | 13.4 (3.2) | 13.2 (3.2) | 12.5 (3.1) |
| Total carbohydrates, g/day^2^ | 248.1 (49.5) | 236.7 (39.8) | 232.9 (38.1) | 230 (38.5) | 222.9 (40.7) |
| Total fibre, g/day^2^ | 20.1 (7.8) | 20.8 (6.6) | 21 (6.3) | 21.5 (6.6) | 21 (7.4) |

1. Values are means (SD) for continuous variables and percentages for categorical variables, and standardised the age distribution of the study population.
2. Adjusted for total energy using the residual method.

Supplementary table 4: Baseline characteristics of participants in the Nurses’ Health Study (NHS), Nurses’ Health Study II (NHSII) and Health Professionals Follow-Up Study (HPFS) by fifths of hydroxyproline intake.

|  | **Fifths of hydroxyproline intake^2^** | | | | |
| --- | --- | --- | --- | --- | --- |
| **Cohort and baseline characteristics^1^** | **Q1** | **Q2** | **Q3** | **Q4** | **Q5** |
| **NHS (1984)** |  |  |  |  |  |
| Number of participants | 15,367 | 14,234 | 14,972 | 13,954 | 15,303 |
| Hydroxyproline intake, g/day^2^ | 0.2 (0) | 0.3 (0) | 0.3 (0) | 0.4 (0) | 0.5 (0.1) |
| Age at baseline, years | 51.1 (7.2) | 50.4 (7.2) | 49.8 (7.1) | 49.8 (7.1) | 49.8 (7) |
| White ethnicity, % | 97.8 | 98.0 | 98.0 | 97.8 | 97.3 |
| Currently married, % | 80.2 | 82.1 | 83.2 | 83.3 | 82.4 |
| Body mass index, kg/m^2^ | 24.1 (4.2) | 24.6 (4.4) | 25 (4.7) | 25.3 (4.8) | 25.9 (5.1) |
| Physical activity, MET-hrs/week | 15.8 (25) | 14.5 (21.1) | 14.1 (21.5) | 13.5 (18.7) | 12.9 (18.2) |
| Smoking status, % |  |  |  |  |  |
| Never smoker | 46.1 | 43.3 | 43.9 | 43.6 | 43.0 |
| Past smoker | 31.2 | 32.4 | 32.7 | 31.1 | 31.3 |
| Current smoker | 22.5 | 24.2 | 23.3 | 25.1 | 25.6 |
| Alcohol consumption, g/day | 7 (12.1) | 7.4 (11.6) | 7.3 (11.4) | 7 (11.1) | 5.8 (9.6) |
| Current postmenopausal hormone use, % | 13.5 | 13.0 | 13.2 | 14.5 | 14.1 |
| Aspirin use, % | 69.3 | 72.0 | 71.2 | 71.9 | 71.2 |
| Multivitamin use, % | 39.2 | 38.0 | 37.1 | 35.6 | 34.8 |
| Baseline diabetes, % | 1.9 | 2.3 | 2.7 | 2.9 | 4.5 |
| Baseline hypertension, % | 18.2 | 19.9 | 20.7 | 22.1 | 24.0 |
| Baseline high blood cholesterol, % | 7.5 | 7.6 | 7.8 | 8.3 | 8.6 |
| Energy intake, kcal/day | 1741 (536.3) | 1745 (535.6) | 1771.2 (530.6) | 1765 (524.6) | 1705.5 (523.7) |
| Total protein, g/day^2^ | 60.5 (10.6) | 66.6 (9.5) | 70.8 (9.6) | 75.4 (9.5) | 84 (11.8) |
| Animal protein, g/day^2^ | 39.7 (10.9) | 46.9 (9.3) | 51.8 (9.4) | 56.9 (9.3) | 66.8 (12.2) |
| Plant protein, g/day^2^ | 20.8 (5.4) | 19.7 (4.1) | 19 (3.8) | 18.5 (3.6) | 17.2 (3.6) |
| Dairy protein, g/day^2^ | 16.6 (8.7) | 15.1 (7.6) | 14.2 (7.1) | 13.3 (6.7) | 11.7 (6.3) |
| Saturated fat, g/day^2^ | 20.5 (5) | 21.4 (4.4) | 21.9 (4.3) | 22.7 (4) | 24.2 (4.2) |
| Monounsaturated fat, g/day^2^ | 20.1 (4.3) | 21.4 (3.8) | 22.2 (3.7) | 23.3 (3.6) | 25.3 (4) |
| Polyunsaturated fat, g/day^2^ | 11.8 (3.5) | 11.9 (3.2) | 11.8 (3) | 11.8 (3) | 11.6 (2.9) |
| Total carbohydrates, g/day^2^ | 208.3 (30.7) | 194.6 (26.2) | 186.3 (25.1) | 176.6 (24) | 159.7 (26) |
| Total fibre, g/day^2^ | 17.4 (5.7) | 16.7 (4.7) | 16.4 (4.6) | 16 (4.3) | 15.2 (4.4) |
|  | **Fifths of hydroxyproline intake^2^** | | | | |
|  | **Q1** | **Q2** | **Q3** | **Q4** | **Q5** |
| **NHSII (1991)** |  |  |  |  |  |
| Number of participants | 19,775 | 16,082 | 20,593 | 18,336 | 17,547 |
| Hydroxyproline intake, g/day^2^ | 0.2 (0.1) | 0.3 (0) | 0.3 (0) | 0.4 (0) | 0.5 (0.1) |
| Age at baseline, years | 35.8 (4.7) | 35.9 (4.7) | 36.1 (4.7) | 36.2 (4.7) | 36.5 (4.6) |
| White ethnicity, % | 96.6 | 97.2 | 96.9 | 96.2 | 95.2 |
| Currently married, % | 87.0 | 90.1 | 90.7 | 90.4 | 90.1 |
| Body mass index, kg/m^2^ | 23.4 (4.6) | 24.1 (5) | 24.5 (5) | 25.1 (5.5) | 26.1 (6.1) |
| Physical activity, MET-hrs/week | 24.8 (32.4) | 21.2 (26.9) | 20.2 (25.3) | 19.3 (24.7) | 18.1 (24.3) |
| Smoking status, % |  |  |  |  |  |
| Never smoker | 66.4 | 65.8 | 65.8 | 65.1 | 64.3 |
| Past smoker | 23.5 | 23.0 | 22.6 | 21.7 | 20.3 |
| Current smoker | 9.9 | 11.0 | 11.5 | 13.1 | 15.3 |
| Alcohol consumption, g/day | 3.4 (6.7) | 3.4 (6.5) | 3.1 (5.8) | 3 (5.8) | 2.6 (5.3) |
| Current postmenopausal hormone use, % | 2.2 | 2.4 | 2.5 | 2.7 | 3.0 |
| Current oral contraceptive use, % | 17.4 | 15.8 | 15.0 | 14.9 | 13.8 |
| Aspirin use, % | 11.2 | 10.8 | 11.0 | 11.1 | 11.9 |
| Multivitamin use, % | 49.2 | 45.8 | 43.8 | 41.5 | 38.3 |
| Baseline diabetes, % | 0.6 | 0.6 | 0.9 | 1.0 | 1.6 |
| Baseline hypertension, % | 4.9 | 5.3 | 6.0 | 6.6 | 8.6 |
| Baseline high blood cholesterol, % | 12.9 | 13.4 | 14.6 | 15.3 | 16.6 |
| Energy intake, kcal/day | 1796.7 (563.2) | 1819.8 (537.3) | 1797.5 (540.8) | 1791.9 (544.1) | 1741.7 (547.3) |
| Total protein, g/day^2^ | 73.3 (12.5) | 81.2 (11.1) | 86.6 (11.3) | 91.8 (11.8) | 100.5 (14.4) |
| Animal protein, g/day^2^ | 48.3 (13.9) | 58.4 (10.9) | 64.3 (11.1) | 70.2 (11.6) | 80.4 (14.7) |
| Plant protein, g/day^2^ | 25.1 (6.6) | 22.9 (4.5) | 22.3 (4.2) | 21.6 (4) | 20.2 (3.8) |
| Dairy protein, g/day^2^ | 21.8 (10.6) | 20 (9) | 18.8 (8.5) | 17.2 (7.9) | 14.8 (7.2) |
| Saturated fat, g/day^2^ | 20.6 (5.4) | 21.8 (4.6) | 22.3 (4.4) | 23.1 (4.3) | 24.7 (4.5) |
| Monounsaturated fat, g/day^2^ | 21.3 (4.9) | 23 (4.4) | 23.8 (4.3) | 24.9 (4.3) | 27.1 (4.6) |
| Polyunsaturated fat, g/day^2^ | 10.8 (3.1) | 11.1 (2.7) | 11.3 (2.6) | 11.5 (2.6) | 11.7 (2.6) |
| Total carbohydrates, g/day^2^ | 254.1 (32.8) | 235.5 (26.2) | 225.2 (24.4) | 213.4 (23.6) | 192.7 (25.6) |
| Total fibre, g/day^2^ | 20.1 (6.9) | 18.5 (5.3) | 18.3 (5) | 17.7 (4.8) | 16.7 (4.6) |
|  |  |  |  |  |  |
|  | **Fifths of hydroxyproline intake^2^** | | | | |
|  | **Q1** | **Q2** | **Q3** | **Q4** | **Q5** |
| **HPFS (1986)** |  |  |  |  |  |
| Number of participants | 8,960 | 7,833 | 8,649 | 8,972 | 8,854 |
| Hydroxyproline intake, g/day^2^ | 0.2 (0.1) | 0.3 (0) | 0.4 (0) | 0.5 (0) | 0.6 (0.1) |
| Age at baseline, years | 53.7 (9.9) | 53.1 (9.7) | 53.1 (9.4) | 53 (9.3) | 52.9 (9.2) |
| White ethnicity, % | 94.7 | 95.4 | 95.4 | 95.4 | 94.1 |
| Currently married, % | 90.8 | 92.8 | 94.0 | 93.7 | 93.4 |
| Body mass index, kg/m^2^ | 24.8 (3.1) | 25.1 (3.1) | 25.4 (3.1) | 25.8 (3.3) | 26.2 (3.6) |
| Physical activity, MET-hrs/week | 24.6 (28.7) | 22.4 (25.9) | 20.5 (24) | 19.2 (22.7) | 17.4 (22) |
| Smoking status, % |  |  |  |  |  |
| Never smoker | 50.7 | 46.8 | 45.8 | 45.0 | 42.2 |
| Past smoker | 40.0 | 42.1 | 42.7 | 42.9 | 44.7 |
| Current smoker | 6.9 | 8.5 | 9.2 | 9.9 | 10.8 |
| Alcohol consumption, g/day | 11.5 (16.9) | 12.4 (16.6) | 11.9 (15.2) | 11.5 (14.8) | 9.6 (13.1) |
| Aspirin use, % | 25.8 | 27.2 | 27.6 | 27.2 | 25.9 |
| Multivitamin use, % | 46.6 | 42.7 | 40.7 | 40.0 | 38.8 |
| Baseline diabetes, % | 1.7 | 1.8 | 2.1 | 2.7 | 4.0 |
| Baseline hypertension, % | 18.2 | 18.4 | 19.9 | 20.7 | 22.3 |
| Baseline high blood cholesterol, % | 10.6 | 10.2 | 10.6 | 10.5 | 10.4 |
| Energy intake, kcal/day | 1993.5 (627.6) | 1997.1 (619.6) | 2028.6 (623.1) | 2010.8 (619.5) | 1944.4 (612.6) |
| Total protein, g/day^2^ | 78.7 (13.2) | 86.3 (12.3) | 91.5 (12.1) | 96.6 (12.4) | 107.3 (16.1) |
| Animal protein, g/day^2^ | 50.6 (13.8) | 60.7 (11.5) | 66.7 (11.3) | 73 (11.7) | 85.7 (16.1) |
| Plant protein, g/day^2^ | 28.2 (8) | 25.7 (5.6) | 24.7 (5) | 23.6 (4.7) | 21.6 (4.7) |
| Dairy protein, g/day^2^ | 19 (10.9) | 17 (9.2) | 15.8 (8.4) | 14.5 (7.8) | 12.5 (7.1) |
| Saturated fat, g/day^2^ | 21.7 (6.7) | 23.3 (5.7) | 24.4 (5.5) | 25.7 (5.3) | 28 (5.6) |
| Monounsaturated fat, g/day^2^ | 24.1 (6.5) | 25.8 (5.5) | 27 (5.1) | 28.6 (4.8) | 31.3 (5.3) |
| Polyunsaturated fat, g/day^2^ | 13.1 (4.2) | 13.1 (3.5) | 13.2 (3.3) | 13.3 (3.1) | 13.3 (3.1) |
| Total carbohydrates, g/day^2^ | 267.9 (43.4) | 247.6 (35.2) | 235.4 (32.7) | 222.2 (30.7) | 198.9 (32.5) |
| Total fibre, g/day^2^ | 23.7 (8.7) | 21.6 (7) | 20.8 (6.1) | 19.9 (5.8) | 18.4 (5.6) |

1. Values are means (SD) for continuous variables and percentages for categorical variables, and standardised the age distribution of the study population.
2. Adjusted for total energy using the residual method.


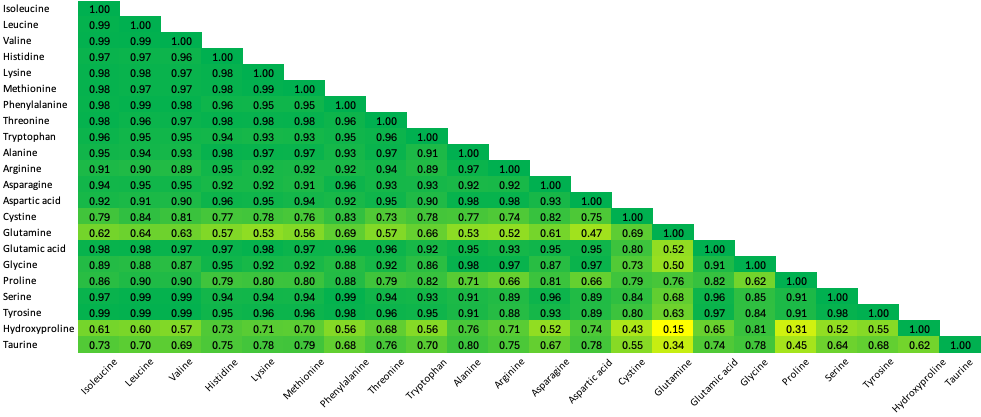


Supplementary figure 2: Correlation matrix between energy-adjusted grams of dietary amino acids in NHS, NHSII and HPFS, based on dietary data collected at mid-point of follow-up (1998 for NHS, 2003 for NHSII, 1998 for HPFS).


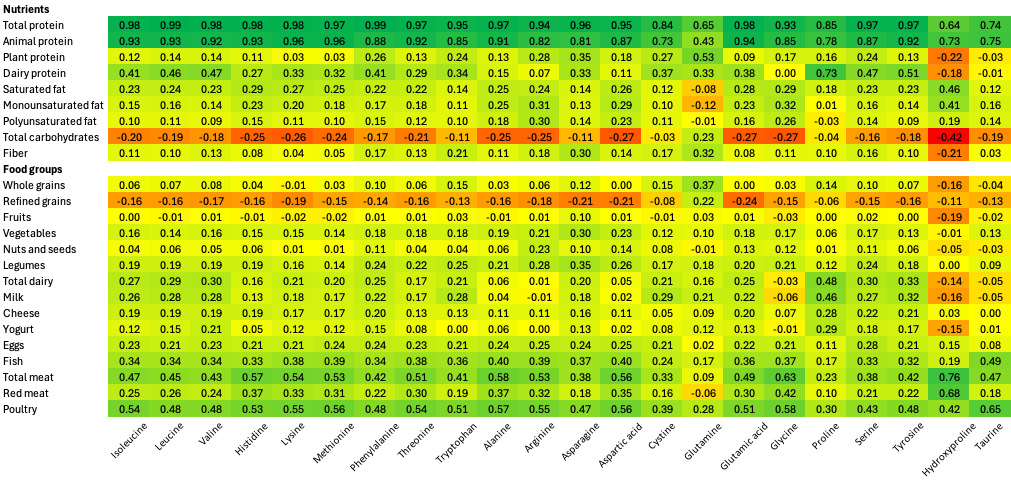


Supplementary figure 3: Correlation matrix between energy-adjusted grams of dietary amino acids and macronutrients and major food groups in NHS, NHSII and HPFS, based on dietary data collected at mid-point of follow-up (1998 for NHS, 2003 for NHSII, 1998 for HPFS).

Supplementary table 5: Hazard ratios (95% confidence intervals) for ischemic stroke (3058 cases) by fifths and per SD difference of energy-adjusted grams of dietary amino acids.

|  | **HR (95% CI), fifths of intake compared to bottom fifth^1^** | | | |  | **HR (95% CI)**  **per SD higher intake^1^** | ***p*-value^3^** |
| --- | --- | --- | --- | --- | --- | --- | --- |
| **Amino acids** | **Q2** | **Q3** | **Q4** | **Q5** | ***p*-trend^2^** |  |  |
| **Isoleucine** |  |  |  |  |  |  |  |
| NHS | 0.91 (0.79, 1.06) | 0.90 (0.77, 1.04) | 0.89 (0.76, 1.03) | 0.93 (0.80, 1.07) | 0.30 | 0.97 (0.92, 1.03) | 0.31 |
| NHSII | 1.43 (0.95, 2.16) | 1.06 (0.68, 1.65) | 0.64 (0.39, 1.05) | 1.19 (0.78, 1.82) | 0.68 | 0.93 (0.79, 1.10) | 0.41 |
| HPFS | 0.81 (0.68, 0.98) | 0.78 (0.65, 0.94) | 0.88 (0.74, 1.06) | 0.84 (0.70, 1.01) | 0.14 | 0.95 (0.88, 1.02) | 0.13 |
| ***Pooled***^4^ | **0.90 (0.81, 1.01)** | **0.86 (0.77, 0.97)** | **0.87 (0.78, 0.97)** | **0.91 (0.81, 1.01)** | **0.07** | **0.96 (0.92, 1.00)** | **0.06** |
| **Leucine** |  |  |  |  |  |  |  |
| NHS | 0.94 (0.81, 1.09) | 0.90 (0.77, 1.04) | 0.90 (0.78, 1.05) | 0.93 (0.80, 1.08) | 0.33 | 0.97 (0.91, 1.02) | 0.24 |
| NHSII | 1.01 (0.67, 1.54) | 1.12 (0.74, 1.69) | 0.58 (0.36, 0.94) | 1.00 (0.66, 1.51) | 0.47 | 0.94 (0.80, 1.11) | 0.49 |
| HPFS | 0.87 (0.73, 1.05) | 0.80 (0.66, 0.96) | 0.87 (0.72, 1.04) | 0.89 (0.74, 1.07) | 0.25 | 0.94 (0.88, 1.01) | 0.11 |
| ***Pooled***^4^ | **0.92 (0.82, 1.03)** | **0.87 (0.78, 0.98)** | **0.87 (0.78, 0.97)** | **0.92 (0.83, 1.03)** | **0.10** | **0.96 (0.92, 1.00)** | **0.04** |
| **Valine** |  |  |  |  |  |  |  |
| NHS | 0.93 (0.80, 1.08) | 0.92 (0.79, 1.07) | 0.90 (0.77, 1.04) | 0.92 (0.79, 1.07) | 0.25 | 0.96 (0.91, 1.02) | 0.19 |
| NHSII | 0.98 (0.65, 1.48) | 1.04 (0.69, 1.56) | 0.54 (0.33, 0.88) | 0.98 (0.65, 1.48) | 0.44 | 0.94 (0.80, 1.10) | 0.44 |
| HPFS | 0.90 (0.75, 1.08) | 0.79 (0.65, 0.95) | 0.87 (0.72, 1.04) | 0.86 (0.72, 1.04) | 0.11 | 0.94 (0.88, 1.01) | 0.10 |
| ***Pooled***^4^ | **0.92 (0.83, 1.03)** | **0.88 (0.79, 0.98)** | **0.86 (0.77, 0.96)** | **0.90 (0.81, 1.01)** | **0.04** | **0.95 (0.91, 1.00)** | **0.03** |
| **Histidine** |  |  |  |  |  |  |  |
| NHS | 0.89 (0.77, 1.04) | 0.83 (0.72, 0.97) | 0.91 (0.78, 1.05) | 0.95 (0.82, 1.10) | 0.59 | 0.98 (0.92, 1.03) | 0.41 |
| NHSII | 1.34 (0.88, 2.05) | 0.89 (0.56, 1.41) | 0.94 (0.60, 1.47) | 1.14 (0.74, 1.75) | 0.94 | 0.94 (0.80, 1.11) | 0.46 |
| HPFS | 0.80 (0.67, 0.97) | 0.86 (0.72, 1.03) | 0.85 (0.71, 1.03) | 0.93 (0.77, 1.11) | 0.56 | 0.96 (0.89, 1.03) | 0.26 |
| ***Pooled***^4^ | **0.88 (0.79, 0.99)** | **0.85 (0.76, 0.95)** | **0.89 (0.80, 1.00)** | **0.95 (0.85, 1.06)** | **0.43** | **0.97 (0.93, 1.01)** | **0.13** |
| **Lysine** |  |  |  |  |  |  |  |
| NHS | 0.87 (0.75, 1.01) | 0.89 (0.77, 1.04) | 0.91 (0.79, 1.06) | 0.91 (0.79, 1.06) | 0.40 | 0.98 (0.92, 1.04) | 0.43 |
| NHSII | 1.19 (0.78, 1.80) | 0.98 (0.64, 1.51) | 0.75 (0.48, 1.19) | 0.99 (0.65, 1.51) | 0.45 | 0.93 (0.79, 1.10) | 0.38 |
| HPFS | 0.87 (0.73, 1.04) | 0.80 (0.66, 0.96) | 0.88 (0.73, 1.06) | 0.90 (0.75, 1.08) | 0.35 | 0.95 (0.89, 1.02) | 0.19 |
| ***Pooled***^4^ | **0.89 (0.80, 1.00)** | **0.86 (0.77, 0.97)** | **0.89 (0.80, 1.00)** | **0.92 (0.82, 1.02)** | **0.15** | **0.96 (0.92, 1.01)** | **0.11** |
| **Methionine** |  |  |  |  |  |  |  |
| NHS | 0.86 (0.74, 0.99) | 0.88 (0.76, 1.02) | 0.87 (0.75, 1.01) | 0.93 (0.80, 1.08) | 0.43 | 0.97 (0.92, 1.03) | 0.34 |
| NHSII | 1.48 (0.98, 2.23) | 1.00 (0.64, 1.57) | 0.83 (0.52, 1.33) | 1.06 (0.69, 1.64) | 0.42 | 0.93 (0.79, 1.10) | 0.40 |
| HPFS | 0.89 (0.74, 1.07) | 0.87 (0.72, 1.04) | 0.86 (0.72, 1.04) | 0.92 (0.76, 1.10) | 0.35 | 0.95 (0.88, 1.02) | 0.13 |
| ***Pooled***^4^ | **0.90 (0.81, 1.01)** | **0.88 (0.79, 0.99)** | **0.86 (0.77, 0.97)** | **0.93 (0.84, 1.04)** | **0.16** | **0.96 (0.92, 1.00)** | **0.06** |
| **Phenylalanine** |  |  |  |  |  |  |  |
| NHS | 0.92 (0.80, 1.07) | 0.89 (0.76, 1.03) | 0.89 (0.77, 1.04) | 0.94 (0.81, 1.09) | 0.39 | 0.96 (0.91, 1.02) | 0.17 |
| NHSII | 0.84 (0.55, 1.27) | 0.87 (0.58, 1.31) | 0.59 (0.37, 0.93) | 0.87 (0.58, 1.30) | 0.30 | 0.93 (0.79, 1.09) | 0.35 |
| HPFS | 0.83 (0.69, 1.00) | 0.80 (0.67, 0.96) | 0.85 (0.71, 1.02) | 0.87 (0.72, 1.04) | 0.16 | 0.95 (0.88, 1.02) | 0.13 |
| ***Pooled***^4^ | **0.88 (0.79, 0.99)** | **0.85 (0.76, 0.95)** | **0.85 (0.76, 0.96)** | **0.91 (0.81, 1.01)** | **0.07** | **0.95 (0.91, 0.99)** | **0.03** |
| **Threonine** |  |  |  |  |  |  |  |
| NHS | 0.93 (0.80, 1.08) | 0.85 (0.73, 0.99) | 0.96 (0.82, 1.11) | 0.95 (0.82, 1.10) | 0.67 | 0.98 (0.92, 1.04) | 0.51 |
| NHSII | 1.37 (0.90, 2.09) | 1.08 (0.69, 1.67) | 0.77 (0.48, 1.24) | 1.12 (0.73, 1.72) | 0.67 | 0.93 (0.79, 1.10) | 0.38 |
| HPFS | 0.90 (0.75, 1.08) | 0.83 (0.69, 1.01) | 0.91 (0.76, 1.09) | 0.93 (0.77, 1.11) | 0.48 | 0.95 (0.88, 1.02) | 0.15 |
| ***Pooled***^4^ | **0.94 (0.84, 1.05)** | **0.86 (0.76, 0.96)** | **0.93 (0.83, 1.04)** | **0.95 (0.85, 1.06)** | **0.37** | **0.96 (0.92, 1.01)** | **0.11** |
| **Tryptophan** |  |  |  |  |  |  |  |
| NHS | 1.03 (0.89, 1.20) | 0.85 (0.73, 1.00) | 0.97 (0.83, 1.12) | 0.96 (0.82, 1.11) | 0.45 | 0.98 (0.92, 1.04) | 0.42 |
| NHSII | 1.25 (0.83, 1.88) | 0.89 (0.58, 1.39) | 0.73 (0.46, 1.16) | 1.05 (0.69, 1.59) | 0.59 | 0.94 (0.80, 1.11) | 0.46 |
| HPFS | 0.87 (0.73, 1.05) | 0.82 (0.68, 0.99) | 0.88 (0.74, 1.06) | 0.86 (0.72, 1.04) | 0.16 | 0.94 (0.88, 1.01) | 0.10 |
| ***Pooled***^4^ | **0.98 (0.88, 1.10)** | **0.84 (0.75, 0.95)** | **0.92 (0.82, 1.03)** | **0.93 (0.83, 1.04)** | **0.11** | **0.96 (0.92, 1.00)** | **0.07** |
| **Alanine** |  |  |  |  |  |  |  |
| NHS | 0.85 (0.74, 0.99) | 0.88 (0.76, 1.02) | 0.91 (0.78, 1.06) | 0.95 (0.82, 1.10) | 0.77 | 0.99 (0.93, 1.05) | 0.72 |
| NHSII | 0.88 (0.58, 1.36) | 0.84 (0.55, 1.29) | 0.86 (0.56, 1.31) | 0.86 (0.57, 1.30) | 0.44 | 0.93 (0.78, 1.10) | 0.37 |
| HPFS | 0.86 (0.71, 1.03) | 0.88 (0.73, 1.06) | 0.90 (0.75, 1.08) | 0.94 (0.78, 1.13) | 0.64 | 0.96 (0.89, 1.03) | 0.27 |
| ***Pooled***^4^ | **0.86 (0.77, 0.96)** | **0.88 (0.78, 0.98)** | **0.90 (0.81, 1.01)** | **0.94 (0.84, 1.05)** | **0.46** | **0.97 (0.93, 1.02)** | **0.24** |
| **Arginine** |  |  |  |  |  |  |  |
| NHS | 0.89 (0.77, 1.03) | 0.84 (0.72, 0.98) | 0.86 (0.74, 0.99) | 0.96 (0.83, 1.12) | 0.62 | 0.99 (0.93, 1.05) | 0.74 |
| NHSII | 0.88 (0.58, 1.33) | 0.77 (0.50, 1.18) | 0.75 (0.49, 1.14) | 0.80 (0.53, 1.21) | 0.22 | 0.89 (0.76, 1.06) | 0.19 |
| HPFS | 0.83 (0.69, 1.00) | 0.88 (0.73, 1.06) | 0.97 (0.81, 1.16) | 0.87 (0.72, 1.04) | 0.36 | 0.96 (0.90, 1.04) | 0.31 |
| ***Pooled***^4^ | **0.87 (0.78, 0.97)** | **0.85 (0.76, 0.95)** | **0.89 (0.80, 0.99)** | **0.92 (0.82, 1.02)** | **0.19** | **0.97 (0.93, 1.02)** | **0.22** |
| **Asparagine** |  |  |  |  |  |  |  |
| NHS | 0.94 (0.81, 1.09) | 0.88 (0.76, 1.03) | 0.92 (0.79, 1.07) | 0.95 (0.82, 1.11) | 0.55 | 0.98 (0.92, 1.04) | 0.44 |
| NHSII | 0.82 (0.54, 1.23) | 0.70 (0.46, 1.06) | 0.62 (0.40, 0.97) | 0.83 (0.56, 1.24) | 0.24 | 0.92 (0.79, 1.08) | 0.32 |
| HPFS | 0.83 (0.69, 1.00) | 0.85 (0.70, 1.02) | 0.89 (0.74, 1.07) | 0.87 (0.73, 1.05) | 0.26 | 0.95 (0.89, 1.02) | 0.20 |
| ***Pooled***^4^ | **0.89 (0.79, 0.99)** | **0.85 (0.76, 0.96)** | **0.89 (0.79, 0.99)** | **0.91 (0.82, 1.02)** | **0.14** | **0.96 (0.92, 1.01)** | **0.10** |
| **Aspartic acid** |  |  |  |  |  |  |  |
| NHS | 0.86 (0.74, 0.99) | 0.82 (0.71, 0.96) | 0.89 (0.77, 1.03) | 0.95 (0.82, 1.10) | 0.79 | 1.00 (0.94, 1.06) | 0.93 |
| NHSII | 1.02 (0.67, 1.56) | 0.80 (0.51, 1.26) | 0.91 (0.59, 1.40) | 0.91 (0.59, 1.38) | 0.56 | 0.92 (0.78, 1.09) | 0.33 |
| HPFS | 0.93 (0.78, 1.12) | 0.88 (0.73, 1.06) | 0.87 (0.72, 1.06) | 0.98 (0.81, 1.17) | 0.66 | 0.96 (0.90, 1.03) | 0.29 |
| ***Pooled***^4^ | **0.89 (0.80, 1.00)** | **0.84 (0.75, 0.94)** | **0.89 (0.79, 0.99)** | **0.95 (0.85, 1.07)** | **0.52** | **0.98 (0.93, 1.02)** | **0.32** |
| **Cystine** |  |  |  |  |  |  |  |
| NHS | 0.94 (0.81, 1.09) | 0.83 (0.72, 0.97) | 0.86 (0.74, 1.00) | 0.93 (0.80, 1.08) | 0.20 | 0.97 (0.92, 1.03) | 0.35 |
| NHSII | 0.95 (0.63, 1.44) | 0.80 (0.52, 1.23) | 0.66 (0.42, 1.04) | 1.06 (0.71, 1.58) | 0.92 | 0.98 (0.85, 1.14) | 0.82 |
| HPFS | 0.86 (0.72, 1.04) | 0.84 (0.70, 1.01) | 0.99 (0.83, 1.19) | 0.82 (0.68, 0.99) | 0.14 | 0.95 (0.89, 1.02) | 0.17 |
| ***Pooled***^4^ | **0.91 (0.82, 1.02)** | **0.83 (0.75, 0.93)** | **0.89 (0.80, 1.00)** | **0.90 (0.80, 1.00)** | **0.06** | **0.97 (0.93, 1.01)** | **0.11** |
| **Glutamine** |  |  |  |  |  |  |  |
| NHS | 0.81 (0.70, 0.93) | 0.83 (0.71, 0.96) | 0.81 (0.70, 0.95) | 0.82 (0.71, 0.96) | 0.01 | 0.94 (0.88, 0.99) | 0.03 |
| NHSII | 1.15 (0.77, 1.72) | 0.99 (0.65, 1.52) | 0.97 (0.63, 1.49) | 0.92 (0.59, 1.43) | 0.56 | 0.94 (0.80, 1.10) | 0.41 |
| HPFS | 0.94 (0.79, 1.12) | 0.82 (0.68, 0.99) | 0.88 (0.73, 1.06) | 0.91 (0.76, 1.10) | 0.19 | 0.94 (0.88, 1.01) | 0.11 |
| ***Pooled***^4^ | **0.88 (0.79, 0.98)** | **0.84 (0.75, 0.93)** | **0.85 (0.76, 0.95)** | **0.86 (0.77, 0.97)** | **0.007** | **0.94 (0.90, 0.98)** | **0.004** |
| **Glutamic acid** |  |  |  |  |  |  |  |
| NHS | 0.87 (0.75, 1.01) | 0.84 (0.72, 0.97) | 0.85 (0.74, 0.99) | 0.89 (0.77, 1.03) | 0.18 | 0.97 (0.92, 1.03) | 0.37 |
| NHSII | 1.18 (0.78, 1.78) | 0.94 (0.61, 1.45) | 0.73 (0.46, 1.16) | 1.01 (0.66, 1.53) | 0.51 | 0.92 (0.78, 1.08) | 0.31 |
| HPFS | 0.80 (0.67, 0.96) | 0.81 (0.67, 0.97) | 0.79 (0.66, 0.95) | 0.88 (0.73, 1.05) | 0.20 | 0.95 (0.88, 1.02) | 0.13 |
| ***Pooled***^4^ | **0.86 (0.77, 0.96)** | **0.83 (0.74, 0.93)** | **0.82 (0.73, 0.92)** | **0.90 (0.80, 1.00)** | **0.05** | **0.96 (0.92, 1.00)** | **0.06** |
| **Glycine** |  |  |  |  |  |  |  |
| NHS | 0.90 (0.78, 1.05) | 0.90 (0.77, 1.04) | 0.94 (0.81, 1.09) | 0.97 (0.84, 1.13) | 0.97 | 1.00 (0.94, 1.06) | 0.93 |
| NHSII | 0.92 (0.60, 1.42) | 0.85 (0.55, 1.31) | 0.98 (0.64, 1.48) | 0.82 (0.54, 1.26) | 0.38 | 0.92 (0.78, 1.09) | 0.34 |
| HPFS | 0.85 (0.70, 1.02) | 0.89 (0.74, 1.08) | 0.93 (0.77, 1.11) | 0.97 (0.81, 1.16) | 0.99 | 0.97 (0.90, 1.04) | 0.34 |
| ***Pooled***^4^ | **0.88 (0.79, 0.99)** | **0.89 (0.80, 1.00)** | **0.94 (0.84, 1.05)** | **0.96 (0.86, 1.07)** | **0.80** | **0.98 (0.94, 1.02)** | **0.37** |
| **Proline** |  |  |  |  |  |  |  |
| NHS | 0.92 (0.80, 1.06) | 0.79 (0.68, 0.92) | 0.94 (0.81, 1.09) | 0.83 (0.71, 0.96) | 0.03 | 0.94 (0.89, 0.99) | 0.02 |
| NHSII | 1.19 (0.79, 1.79) | 1.01 (0.66, 1.55) | 0.81 (0.51, 1.28) | 1.08 (0.71, 1.65) | 0.85 | 0.97 (0.83, 1.14) | 0.73 |
| HPFS | 0.92 (0.77, 1.10) | 0.88 (0.74, 1.06) | 0.71 (0.58, 0.86) | 0.86 (0.71, 1.03) | 0.02 | 0.94 (0.88, 1.01) | 0.09 |
| ***Pooled***^4^ | **0.94 (0.84, 1.04)** | **0.84 (0.75, 0.94)** | **0.84 (0.75, 0.95)** | **0.85 (0.76, 0.96)** | **0.002** | **0.94 (0.90, 0.98)** | **0.005** |
| **Serine** |  |  |  |  |  |  |  |
| NHS | 0.90 (0.78, 1.04) | 0.88 (0.76, 1.02) | 0.87 (0.75, 1.01) | 0.93 (0.80, 1.08) | 0.32 | 0.96 (0.90, 1.01) | 0.14 |
| NHSII | 0.93 (0.61, 1.40) | 0.85 (0.56, 1.29) | 0.66 (0.42, 1.03) | 0.91 (0.61, 1.37) | 0.41 | 0.94 (0.80, 1.11) | 0.47 |
| HPFS | 0.85 (0.71, 1.02) | 0.84 (0.70, 1.01) | 0.83 (0.69, 0.99) | 0.85 (0.70, 1.02) | 0.08 | 0.95 (0.88, 1.02) | 0.14 |
| ***Pooled***^4^ | **0.88 (0.79, 0.98)** | **0.86 (0.77, 0.96)** | **0.84 (0.75, 0.94)** | **0.90 (0.80, 1.01)** | **0.04** | **0.95 (0.91, 0.99)** | **0.03** |
| **Tyrosine** |  |  |  |  |  |  |  |
| NHS | 0.90 (0.78, 1.04) | 0.94 (0.81, 1.09) | 0.87 (0.75, 1.01) | 0.92 (0.79, 1.06) | 0.23 | 0.96 (0.90, 1.01) | 0.14 |
| NHSII | 1.02 (0.67, 1.56) | 1.05 (0.69, 1.59) | 0.68 (0.42, 1.08) | 0.99 (0.65, 1.50) | 0.57 | 0.93 (0.79, 1.10) | 0.40 |
| HPFS | 0.89 (0.75, 1.07) | 0.77 (0.63, 0.92) | 0.85 (0.70, 1.02) | 0.87 (0.72, 1.04) | 0.10 | 0.94 (0.87, 1.01) | 0.07 |
| ***Pooled***^4^ | **0.91 (0.81, 1.01)** | **0.88 (0.79, 0.98)** | **0.85 (0.76, 0.95)** | **0.90 (0.81, 1.01)** | **0.04** | **0.95 (0.91, 0.99)** | **0.015** |
| **Hydroxyproline** |  |  |  |  |  |  |  |
| NHS | 1.03 (0.88, 1.19) | 1.03 (0.89, 1.21) | 1.03 (0.89, 1.21) | 1.12 (0.97, 1.30) | 0.11 | 1.03 (0.97, 1.09) | 0.33 |
| NHSII | 0.71 (0.44, 1.15) | 1.10 (0.72, 1.68) | 0.95 (0.62, 1.47) | 0.89 (0.58, 1.37) | 0.79 | 0.99 (0.84, 1.15) | 0.87 |
| HPFS | 1.02 (0.85, 1.24) | 1.03 (0.85, 1.24) | 1.06 (0.87, 1.28) | 1.15 (0.95, 1.39) | 0.12 | 1.02 (0.95, 1.09) | 0.61 |
| ***Pooled***^4^ | **1.00 (0.90, 1.13)** | **1.04 (0.92, 1.16)** | **1.04 (0.92, 1.16)** | **1.11 (0.99, 1.25)** | **0.04** | **1.02 (0.98, 1.07)** | **0.33** |
| **Taurine** |  |  |  |  |  |  |  |
| NHS | 1.02 (0.88, 1.19) | 0.93 (0.80, 1.08) | 0.94 (0.81, 1.09) | 1.00 (0.86, 1.16) | 0.78 | 0.99 (0.94, 1.05) | 0.79 |
| NHSII | 0.75 (0.48, 1.16) | 0.94 (0.62, 1.42) | 0.82 (0.54, 1.25) | 0.84 (0.56, 1.27) | 0.47 | 0.96 (0.80, 1.15) | 0.67 |
| HPFS | 0.80 (0.66, 0.96) | 0.86 (0.71, 1.03) | 0.85 (0.71, 1.02) | 0.88 (0.73, 1.05) | 0.31 | 0.96 (0.89, 1.03) | 0.26 |
| ***Pooled***^4^ | **0.92 (0.82, 1.03)** | **0.90 (0.81, 1.01)** | **0.90 (0.80, 1.00)** | **0.94 (0.84, 1.05)** | **0.27** | **0.98 (0.94, 1.02)** | **0.32** |

1. Analyses were stratified by age (in years) and follow-up intervals, and adjusted for ethnicity (white, non-white), marital status (married, widowed, divorced/separated, unknown), smoking status (never, past, current: 1–14, 15–24, ≥25 cigarettes/day, unknown status or cigarette number), alcohol intake (never drinkers, 0.1-4.9, 5.0-9.9, 10.0-14.9, 15.0+ g/d), physical activity (<3, 3–9, 9–18, 18–27, 27–42, ≥42 metabolic equivalents/week, unknown), menopausal status and postmenopausal hormone use (premenopausal, postmenopausal: never, past, current users, NHS and NHSII only), oral contraception use (never, past, current user, NHSII only), multivitamin use (no, yes), aspirin use (no, yes), body mass index (<23, 23–24.9, 25–29.9, 30–34.9, ≥35 kg/m2, missing), baseline history of hypertension (no, yes), baseline hypercholesteremia (no, yes), baseline diabetes (no, yes), and energy intake (fifths of intake).
2. Linear trends estimated by fitting the median values in each fifth of intake as a pseudo-continuous variable in the Cox regression.
3. P-values estimated by fitting the continuous intakes of dietary amino acids in the Cox regression.
4. Results from NHS, NHSII and HPFS were pooled using a fixed-effects meta-analysis.

Supplementary table 6: Hazard ratios (95% confidence intervals) for hemorrhagic stroke (872 cases) by fifths and per SD difference of energy-adjusted grams of dietary amino acids.

|  | **HR (95% CI), fifths of intake compared to bottom fifth^1^** | | | |  | **HR (95% CI)**  **per SD higher intake^1^** | ***p*-value^3^** |
| --- | --- | --- | --- | --- | --- | --- | --- |
| **Amino acids** | **Q2** | **Q3** | **Q4** | **Q5** | ***p*-trend^2^** |  |  |
| **Isoleucine** |  |  |  |  |  |  |  |
| NHS | 1.17 (0.89, 1.52) | 0.90 (0.68, 1.20) | 0.98 (0.74, 1.30) | 0.99 (0.75, 1.32) | 0.62 | 0.95 (0.85, 1.06) | 0.33 |
| NHSII | 1.31 (0.70, 2.44) | 1.37 (0.74, 2.56) | 1.11 (0.57, 2.14) | 0.88 (0.44, 1.78) | 0.69 | 1.00 (0.78, 1.28) | 1.00 |
| HPFS | 1.14 (0.80, 1.63) | 1.00 (0.69, 1.44) | 0.90 (0.62, 1.32) | 0.96 (0.66, 1.40) | 0.47 | 0.98 (0.85, 1.12) | 0.74 |
| ***Pooled***^4^ | **1.17 (0.96, 1.44)** | **0.98 (0.79, 1.21)** | **0.97 (0.78, 1.20)** | **0.97 (0.78, 1.20)** | **0.34** | **0.96 (0.89, 1.04)** | **0.36** |
| **Leucine** |  |  |  |  |  |  |  |
| NHS | 1.19 (0.91, 1.55) | 0.92 (0.69, 1.23) | 0.99 (0.75, 1.32) | 1.00 (0.75, 1.34) | 0.67 | 0.95 (0.85, 1.06) | 0.37 |
| NHSII | 1.25 (0.66, 2.35) | 1.44 (0.78, 2.67) | 1.23 (0.64, 2.35) | 0.77 (0.37, 1.59) | 0.62 | 0.99 (0.78, 1.27) | 0.95 |
| HPFS | 1.14 (0.80, 1.63) | 0.89 (0.60, 1.30) | 1.08 (0.75, 1.55) | 0.96 (0.66, 1.40) | 0.71 | 0.98 (0.85, 1.12) | 0.78 |
| ***Pooled***^4^ | **1.18 (0.96, 1.44)** | **0.96 (0.78, 1.19)** | **1.05 (0.85, 1.29)** | **0.97 (0.78, 1.20)** | **0.49** | **0.97 (0.89, 1.05)** | **0.40** |
| **Valine** |  |  |  |  |  |  |  |
| NHS | 1.11 (0.85, 1.46) | 0.94 (0.71, 1.25) | 0.94 (0.70, 1.25) | 0.97 (0.73, 1.29) | 0.55 | 0.95 (0.85, 1.06) | 0.34 |
| NHSII | 1.02 (0.54, 1.92) | 1.18 (0.64, 2.18) | 1.16 (0.62, 2.17) | 0.75 (0.37, 1.51) | 0.66 | 1.00 (0.78, 1.27) | 0.97 |
| HPFS | 1.17 (0.82, 1.67) | 0.87 (0.60, 1.28) | 1.02 (0.70, 1.47) | 0.96 (0.66, 1.40) | 0.59 | 0.97 (0.85, 1.12) | 0.71 |
| ***Pooled***^4^ | **1.12 (0.92, 1.37)** | **0.95 (0.77, 1.17)** | **0.99 (0.80, 1.22)** | **0.95 (0.76, 1.17)** | **0.36** | **0.96 (0.89, 1.04)** | **0.34** |
| **Histidine** |  |  |  |  |  |  |  |
| NHS | 1.11 (0.85, 1.46) | 1.04 (0.79, 1.38) | 1.05 (0.79, 1.40) | 1.02 (0.77, 1.36) | 0.96 | 0.97 (0.87, 1.09) | 0.64 |
| NHSII | 0.95 (0.50, 1.80) | 1.00 (0.53, 1.88) | 1.28 (0.70, 2.34) | 0.77 (0.39, 1.53) | 0.83 | 0.98 (0.77, 1.25) | 0.87 |
| HPFS | 0.96 (0.67, 1.37) | 0.80 (0.55, 1.16) | 1.02 (0.72, 1.45) | 0.81 (0.55, 1.18) | 0.34 | 0.97 (0.84, 1.11) | 0.66 |
| ***Pooled***^4^ | **1.04 (0.85, 1.28)** | **0.95 (0.77, 1.18)** | **1.07 (0.87, 1.31)** | **0.92 (0.74, 1.14)** | **0.46** | **0.97 (0.90, 1.06)** | **0.51** |
| **Lysine** |  |  |  |  |  |  |  |
| NHS | 1.07 (0.82, 1.40) | 1.00 (0.76, 1.32) | 0.92 (0.69, 1.22) | 1.00 (0.75, 1.32) | 0.69 | 0.96 (0.86, 1.07) | 0.48 |
| NHSII | 1.06 (0.55, 2.04) | 1.35 (0.73, 2.52) | 1.31 (0.70, 2.47) | 0.85 (0.43, 1.72) | 0.95 | 0.99 (0.77, 1.27) | 0.94 |
| HPFS | 1.09 (0.76, 1.55) | 0.89 (0.61, 1.29) | 1.09 (0.76, 1.56) | 0.87 (0.59, 1.27) | 0.47 | 0.98 (0.86, 1.13) | 0.82 |
| ***Pooled***^4^ | **1.07 (0.88, 1.32)** | **1.00 (0.81, 1.23)** | **1.01 (0.82, 1.25)** | **0.94 (0.76, 1.16)** | **0.44** | **0.97 (0.90, 1.05)** | **0.49** |
| **Methionine** |  |  |  |  |  |  |  |
| NHS | 1.17 (0.89, 1.52) | 0.90 (0.67, 1.20) | 1.00 (0.75, 1.33) | 1.02 (0.77, 1.36) | 0.81 | 0.95 (0.85, 1.06) | 0.35 |
| NHSII | 1.27 (0.66, 2.43) | 1.46 (0.78, 2.75) | 1.35 (0.71, 2.58) | 0.92 (0.45, 1.87) | 0.99 | 0.99 (0.77, 1.27) | 0.93 |
| HPFS | 1.06 (0.75, 1.52) | 0.82 (0.56, 1.19) | 1.07 (0.75, 1.52) | 0.87 (0.60, 1.26) | 0.44 | 0.98 (0.85, 1.12) | 0.74 |
| ***Pooled***^4^ | **1.14 (0.93, 1.40)** | **0.92 (0.74, 1.15)** | **1.06 (0.86, 1.30)** | **0.96 (0.77, 1.19)** | **0.49** | **0.96 (0.89, 1.04)** | **0.36** |
| **Phenylalanine** |  |  |  |  |  |  |  |
| NHS | 1.03 (0.79, 1.35) | 0.95 (0.72, 1.26) | 0.92 (0.69, 1.22) | 1.01 (0.76, 1.34) | 0.83 | 0.95 (0.86, 1.06) | 0.39 |
| NHSII | 1.22 (0.66, 2.27) | 1.36 (0.74, 2.51) | 1.04 (0.54, 2.00) | 0.72 (0.35, 1.49) | 0.42 | 0.97 (0.76, 1.24) | 0.82 |
| HPFS | 0.91 (0.64, 1.30) | 0.82 (0.57, 1.18) | 0.85 (0.59, 1.23) | 0.86 (0.60, 1.24) | 0.36 | 0.97 (0.85, 1.11) | 0.67 |
| ***Pooled***^4^ | **1.01 (0.82, 1.24)** | **0.95 (0.77, 1.16)** | **0.91 (0.73, 1.12)** | **0.93 (0.75, 1.15)** | **0.30** | **0.96 (0.89, 1.04)** | **0.34** |
| **Threonine** |  |  |  |  |  |  |  |
| NHS | 1.18 (0.90, 1.54) | 1.00 (0.75, 1.33) | 1.05 (0.79, 1.39) | 0.99 (0.74, 1.32) | 0.73 | 0.96 (0.86, 1.07) | 0.43 |
| NHSII | 1.02 (0.54, 1.91) | 1.28 (0.70, 2.32) | 1.02 (0.54, 1.93) | 0.72 (0.36, 1.45) | 0.52 | 0.96 (0.75, 1.23) | 0.76 |
| HPFS | 1.05 (0.73, 1.50) | 0.86 (0.59, 1.25) | 1.06 (0.74, 1.52) | 0.84 (0.57, 1.23) | 0.38 | 0.98 (0.86, 1.13) | 0.82 |
| ***Pooled***^4^ | **1.12 (0.91, 1.37)** | **0.98 (0.80, 1.22)** | **1.05 (0.85, 1.29)** | **0.91 (0.73, 1.13)** | **0.30** | **0.97 (0.89, 1.05)** | **0.41** |
| **Tryptophan** |  |  |  |  |  |  |  |
| NHS | 1.23 (0.93, 1.61) | 1.07 (0.80, 1.42) | 0.98 (0.73, 1.31) | 1.06 (0.80, 1.41) | 0.84 | 0.96 (0.86, 1.08) | 0.49 |
| NHSII | 1.68 (0.89, 3.15) | 1.65 (0.88, 3.11) | 0.98 (0.48, 2.03) | 1.06 (0.52, 2.15) | 0.72 | 0.97 (0.76, 1.24) | 0.80 |
| HPFS | 1.12 (0.79, 1.60) | 0.91 (0.62, 1.32) | 0.91 (0.63, 1.32) | 0.92 (0.63, 1.34) | 0.40 | 0.98 (0.85, 1.12) | 0.75 |
| ***Pooled***^4^ | **1.23 (1.00, 1.51)** | **1.06 (0.86, 1.32)** | **0.95 (0.76, 1.19)** | **1.01 (0.81, 1.26)** | **0.42** | **0.97 (0.89, 1.05)** | **0.43** |
| **Alanine** |  |  |  |  |  |  |  |
| NHS | 1.01 (0.77, 1.33) | 0.99 (0.75, 1.31) | 1.12 (0.85, 1.47) | 0.91 (0.68, 1.22) | 0.72 | 0.97 (0.87, 1.09) | 0.64 |
| NHSII | 1.17 (0.63, 2.18) | 1.04 (0.55, 1.97) | 1.14 (0.61, 2.13) | 0.84 (0.42, 1.65) | 0.69 | 0.95 (0.74, 1.22) | 0.68 |
| HPFS | 0.90 (0.63, 1.29) | 0.85 (0.59, 1.23) | 1.02 (0.72, 1.46) | 0.86 (0.59, 1.24) | 0.54 | 0.99 (0.86, 1.14) | 0.89 |
| ***Pooled***^4^ | **0.99 (0.80, 1.21)** | **0.95 (0.77, 1.17)** | **1.09 (0.89, 1.33)** | **0.89 (0.71, 1.10)** | **0.43** | **0.98 (0.90, 1.06)** | **0.57** |
| **Arginine** |  |  |  |  |  |  |  |
| NHS | 0.92 (0.70, 1.22) | 1.08 (0.82, 1.42) | 1.03 (0.78, 1.36) | 0.91 (0.68, 1.21) | 0.68 | 0.97 (0.86, 1.08) | 0.56 |
| NHSII | 1.36 (0.73, 2.53) | 1.22 (0.65, 2.30) | 0.92 (0.47, 1.82) | 1.01 (0.52, 1.96) | 0.74 | 0.95 (0.74, 1.22) | 0.67 |
| HPFS | 0.97 (0.68, 1.39) | 0.99 (0.69, 1.41) | 0.83 (0.57, 1.21) | 0.92 (0.63, 1.32) | 0.44 | 1.00 (0.87, 1.15) | 0.96 |
| ***Pooled***^4^ | **0.98 (0.80, 1.21)** | **1.06 (0.87, 1.30)** | **0.95 (0.77, 1.18)** | **0.92 (0.74, 1.14)** | **0.37** | **0.98 (0.90, 1.06)** | **0.59** |
| **Asparagine** |  |  |  |  |  |  |  |
| NHS | 0.95 (0.72, 1.24) | 0.95 (0.72, 1.25) | 0.89 (0.67, 1.18) | 0.88 (0.66, 1.17) | 0.33 | 0.93 (0.83, 1.04) | 0.20 |
| NHSII | 1.51 (0.83, 2.77) | 1.21 (0.64, 2.28) | 0.99 (0.50, 1.93) | 0.78 (0.38, 1.59) | 0.31 | 0.93 (0.73, 1.18) | 0.54 |
| HPFS | 0.95 (0.66, 1.36) | 0.96 (0.67, 1.37) | 0.82 (0.57, 1.20) | 0.87 (0.60, 1.26) | 0.32 | 0.97 (0.85, 1.12) | 0.70 |
| ***Pooled***^4^ | **1.00 (0.82, 1.23)** | **0.97 (0.79, 1.20)** | **0.88 (0.71, 1.09)** | **0.87 (0.70, 1.07)** | **0.10** | **0.94 (0.87, 1.02)** | **0.17** |
| **Aspartic acid** |  |  |  |  |  |  |  |
| NHS | 1.03 (0.78, 1.35) | 0.90 (0.68, 1.20) | 1.04 (0.79, 1.37) | 1.00 (0.75, 1.33) | 0.99 | 0.96 (0.86, 1.08) | 0.53 |
| NHSII | 1.15 (0.63, 2.11) | 0.87 (0.45, 1.67) | 1.07 (0.57, 1.99) | 0.78 (0.40, 1.52) | 0.47 | 0.94 (0.73, 1.21) | 0.64 |
| HPFS | 0.93 (0.65, 1.32) | 0.93 (0.65, 1.33) | 0.81 (0.56, 1.18) | 0.90 (0.62, 1.30) | 0.42 | 1.00 (0.87, 1.15) | 0.98 |
| ***Pooled***^4^ | **1.01 (0.82, 1.23)** | **0.91 (0.74, 1.12)** | **0.96 (0.78, 1.19)** | **0.94 (0.76, 1.16)** | **0.43** | **0.97 (0.90, 1.06)** | **0.55** |
| **Cystine** |  |  |  |  |  |  |  |
| NHS | 1.05 (0.81, 1.37) | 0.88 (0.66, 1.17) | 0.97 (0.74, 1.29) | 0.89 (0.66, 1.19) | 0.34 | 0.93 (0.84, 1.03) | 0.19 |
| NHSII | 1.26 (0.67, 2.35) | 1.34 (0.72, 2.50) | 1.02 (0.52, 1.99) | 0.91 (0.46, 1.82) | 0.70 | 0.98 (0.78, 1.24) | 0.88 |
| HPFS | 0.70 (0.48, 1.01) | 0.89 (0.63, 1.26) | 0.81 (0.57, 1.17) | 0.78 (0.54, 1.12) | 0.31 | 0.96 (0.84, 1.10) | 0.54 |
| ***Pooled***^4^ | **0.94 (0.77, 1.16)** | **0.92 (0.75, 1.14)** | **0.92 (0.75, 1.13)** | **0.85 (0.69, 1.06)** | **0.15** | **0.95 (0.87, 1.02)** | **0.16** |
| **Glutamine** |  |  |  |  |  |  |  |
| NHS | 0.99 (0.76, 1.29) | 1.01 (0.77, 1.33) | 0.76 (0.56, 1.02) | 0.93 (0.70, 1.23) | 0.26 | 0.90 (0.81, 1.00) | 0.05 |
| NHSII | 1.27 (0.70, 2.32) | 0.94 (0.49, 1.82) | 1.09 (0.57, 2.07) | 0.91 (0.46, 1.79) | 0.75 | 0.94 (0.74, 1.20) | 0.63 |
| HPFS | 0.86 (0.61, 1.23) | 0.89 (0.62, 1.27) | 0.83 (0.57, 1.20) | 0.81 (0.55, 1.18) | 0.25 | 0.90 (0.78, 1.04) | 0.16 |
| ***Pooled***^4^ | **0.97 (0.80, 1.19)** | **0.96 (0.79, 1.18)** | **0.81 (0.65, 1.01)** | **0.88 (0.71, 1.10)** | **0.11** | **0.91 (0.83, 0.98)** | **0.02** |
| **Glutamic acid** |  |  |  |  |  |  |  |
| NHS | 1.05 (0.80, 1.37) | 0.92 (0.70, 1.23) | 0.94 (0.71, 1.25) | 0.97 (0.73, 1.29) | 0.68 | 0.94 (0.84, 1.06) | 0.32 |
| NHSII | 1.06 (0.56, 2.01) | 1.11 (0.59, 2.08) | 1.23 (0.66, 2.29) | 0.86 (0.44, 1.69) | 0.88 | 1.00 (0.78, 1.27) | 0.99 |
| HPFS | 1.04 (0.72, 1.49) | 1.01 (0.70, 1.46) | 1.07 (0.75, 1.54) | 0.88 (0.60, 1.30) | 0.57 | 0.99 (0.87, 1.14) | 0.91 |
| ***Pooled***^4^ | **1.04 (0.85, 1.28)** | **0.97 (0.79, 1.20)** | **1.01 (0.82, 1.25)** | **0.93 (0.75, 1.15)** | **0.48** | **0.97 (0.89, 1.05)** | **0.42** |
| **Glycine** |  |  |  |  |  |  |  |
| NHS | 0.92 (0.69, 1.21) | 1.01 (0.77, 1.33) | 1.05 (0.80, 1.38) | 0.93 (0.70, 1.23) | 0.82 | 1.00 (0.89, 1.11) | 0.95 |
| NHSII | 1.44 (0.76, 2.76) | 1.41 (0.74, 2.69) | 1.26 (0.65, 2.45) | 0.98 (0.48, 1.98) | 0.86 | 0.97 (0.76, 1.24) | 0.80 |
| HPFS | 0.69 (0.48, 1.00) | 0.70 (0.49, 1.01) | 0.90 (0.64, 1.26) | 0.76 (0.53, 1.09) | 0.32 | 0.99 (0.86, 1.14) | 0.93 |
| ***Pooled***^4^ | **0.88 (0.71, 1.08)** | **0.93 (0.75, 1.14)** | **1.01 (0.82, 1.24)** | **0.87 (0.70, 1.08)** | **0.38** | **0.99 (0.91, 1.08)** | **0.85** |
| **Proline** |  |  |  |  |  |  |  |
| NHS | 1.12 (0.86, 1.46) | 0.88 (0.66, 1.18) | 0.92 (0.69, 1.23) | 1.00 (0.75, 1.33) | 0.64 | 0.95 (0.85, 1.06) | 0.34 |
| NHSII | 1.78 (0.98, 3.25) | 0.97 (0.48, 1.95) | 1.07 (0.53, 2.15) | 1.29 (0.66, 2.54) | 0.91 | 1.03 (0.82, 1.31) | 0.78 |
| HPFS | 0.76 (0.53, 1.09) | 0.89 (0.63, 1.26) | 0.65 (0.44, 0.96) | 0.91 (0.64, 1.30) | 0.47 | 0.96 (0.84, 1.10) | 0.54 |
| ***Pooled***^4^ | **1.05 (0.85, 1.28)** | **0.89 (0.72, 1.10)** | **0.84 (0.67, 1.04)** | **0.99 (0.81, 1.23)** | **0.46** | **0.96 (0.89, 1.04)** | **0.33** |
| **Serine** |  |  |  |  |  |  |  |
| NHS | 1.12 (0.86, 1.47) | 0.93 (0.70, 1.23) | 0.90 (0.67, 1.20) | 1.00 (0.76, 1.33) | 0.60 | 0.95 (0.85, 1.06) | 0.35 |
| NHSII | 1.27 (0.69, 2.34) | 1.21 (0.64, 2.26) | 1.22 (0.65, 2.29) | 0.68 (0.32, 1.42) | 0.42 | 0.99 (0.78, 1.26) | 0.94 |
| HPFS | 1.17 (0.82, 1.66) | 0.80 (0.55, 1.18) | 0.99 (0.69, 1.44) | 0.95 (0.65, 1.38) | 0.56 | 0.97 (0.84, 1.11) | 0.65 |
| ***Pooled***^4^ | **1.15 (0.94, 1.41)** | **0.91 (0.74, 1.13)** | **0.96 (0.78, 1.19)** | **0.95 (0.77, 1.18)** | **0.31** | **0.96 (0.89, 1.04)** | **0.32** |
| **Tyrosine** |  |  |  |  |  |  |  |
| NHS | 1.05 (0.80, 1.38) | 1.06 (0.81, 1.40) | 0.91 (0.69, 1.22) | 0.94 (0.71, 1.25) | 0.49 | 0.96 (0.86, 1.06) | 0.41 |
| NHSII | 1.02 (0.53, 1.95) | 1.35 (0.74, 2.48) | 1.24 (0.66, 2.33) | 0.80 (0.39, 1.61) | 0.82 | 1.00 (0.79, 1.28) | 0.98 |
| HPFS | 1.15 (0.81, 1.64) | 0.88 (0.60, 1.28) | 1.06 (0.74, 1.53) | 0.91 (0.62, 1.33) | 0.53 | 0.97 (0.85, 1.12) | 0.70 |
| ***Pooled***^4^ | **1.08 (0.88, 1.33)** | **1.03 (0.84, 1.27)** | **1.00 (0.81, 1.23)** | **0.92 (0.74, 1.14)** | **0.34** | **0.97 (0.89, 1.05)** | **0.41** |
| **Hydroxyproline** |  |  |  |  |  |  |  |
| NHS | 1.03 (0.78, 1.37) | 1.15 (0.87, 1.52) | 1.03 (0.77, 1.38) | 1.13 (0.85, 1.50) | 0.46 | 1.04 (0.93, 1.17) | 0.47 |
| NHSII | 1.36 (0.71, 2.62) | 1.42 (0.75, 2.71) | 1.26 (0.64, 2.46) | 0.95 (0.46, 1.94) | 0.79 | 0.97 (0.77, 1.23) | 0.83 |
| HPFS | 1.00 (0.71, 1.42) | 0.87 (0.60, 1.24) | 0.78 (0.53, 1.14) | 1.00 (0.70, 1.44) | 0.71 | 0.99 (0.86, 1.13) | 0.84 |
| ***Pooled***^4^ | **1.05 (0.85, 1.29)** | **1.07 (0.87, 1.32)** | **0.96 (0.77, 1.20)** | **1.07 (0.86, 1.32)** | **0.88** | **1.01 (0.93, 1.10)** | **0.75** |
| **Taurine** |  |  |  |  |  |  |  |
| NHS | 1.06 (0.80, 1.39) | 0.99 (0.75, 1.31) | 0.90 (0.68, 1.20) | 0.99 (0.75, 1.30) | 0.61 | 0.94 (0.84, 1.06) | 0.32 |
| NHSII | 1.43 (0.75, 2.74) | 1.18 (0.59, 2.34) | 1.21 (0.62, 2.34) | 1.25 (0.65, 2.43) | 0.70 | 1.03 (0.80, 1.33) | 0.81 |
| HPFS | 1.00 (0.70, 1.45) | 0.97 (0.67, 1.41) | 0.96 (0.66, 1.39) | 1.04 (0.72, 1.49) | 0.96 | 1.01 (0.89, 1.15) | 0.83 |
| ***Pooled***^4^ | **1.07 (0.87, 1.32)** | **1.00 (0.81, 1.24)** | **0.95 (0.77, 1.18)** | **1.03 (0.83, 1.27)** | **0.87** | - 1. **(0.90, 1.06)** | **0.61** |

1. Analyses were stratified by age (in years) and follow-up intervals, and adjusted for ethnicity (white, non-white), marital status (married, widowed, divorced/separated, unknown), smoking status (never, past, current: 1–14, 15–24, ≥25 cigarettes/day, unknown status or cigarette number), alcohol intake (never drinkers, 0.1-4.9, 5.0-9.9, 10.0-14.9, 15.0+ g/d), physical activity (<3, 3–9, 9–18, 18–27, 27–42, ≥42 metabolic equivalents/week, unknown), menopausal status and postmenopausal hormone use (premenopausal, postmenopausal: never, past, current users, NHS and NHSII only), oral contraception use (never, past, current user, NHSII only), multivitamin use (no, yes), aspirin use (no, yes), body mass index (<23, 23–24.9, 25–29.9, 30–34.9, ≥35 kg/m2, missing), baseline history of hypertension (no, yes), baseline hypercholesteremia (no, yes), baseline diabetes (no, yes), and energy intake (fifths of intake).
2. Linear trends estimated by fitting the median values in each fifth of intake as a pseudo-continuous variable in the Cox regression.
3. P-values estimated by fitting the continuous intakes of dietary amino acids in the Cox regression.
4. Results from NHS, NHSII and HPFS were pooled using a fixed-effects meta-analysis.

Supplementary table 7: Hazard ratios (95% confidence intervals) for total stroke (5997 cases) by fifths and per SD difference of energy-adjusted grams of dietary amino acids.

|  | **HR (95% CI), fifths of intake compared to bottom fifth^1^** | | | |  | **HR (95% CI)**  **per SD higher intake^1^** | ***p*-value^3^** |
| --- | --- | --- | --- | --- | --- | --- | --- |
| **Amino acids** | **Q2** | **Q3** | **Q4** | **Q5** | ***p*-trend^2^** |  |  |
| **Isoleucine** |  |  |  |  |  |  |  |
| NHS | 0.99 (0.89, 1.10) | 0.91 (0.82, 1.02) | 0.94 (0.85, 1.05) | 0.97 (0.87, 1.08) | 0.44 | 0.97 (0.93, 1.02) | 0.21 |
| NHSII | 1.23 (0.95, 1.58) | 1.03 (0.79, 1.34) | 0.85 (0.64, 1.12) | 1.17 (0.91, 1.51) | 0.78 | 1.00 (0.91, 1.10) | 1.00 |
| HPFS | 0.91 (0.79, 1.04) | 0.88 (0.77, 1.01) | 0.94 (0.82, 1.07) | 0.96 (0.84, 1.10) | 0.75 | 0.99 (0.94, 1.04) | 0.74 |
| ***Pooled***^4^ | **0.98 (0.90, 1.06)** | **0.91 (0.84, 0.99)** | **0.93 (0.86, 1.01)** | **0.98 (0.91, 1.07)** | **0.53** | **0.98 (0.95, 1.01)** | **0.26** |
| **Leucine** |  |  |  |  |  |  |  |
| NHS | 0.99 (0.89, 1.10) | 0.92 (0.82, 1.02) | 0.96 (0.86, 1.07) | 0.96 (0.86, 1.07) | 0.44 | 0.97 (0.93, 1.01) | 0.16 |
| NHSII | 0.94 (0.73, 1.22) | 1.07 (0.83, 1.38) | 0.79 (0.60, 1.04) | 1.04 (0.81, 1.33) | 0.98 | 1.00 (0.91, 1.10) | 0.97 |
| HPFS | 0.92 (0.81, 1.06) | 0.87 (0.76, 1.00) | 0.96 (0.84, 1.10) | 0.99 (0.86, 1.13) | 1.00 | 0.99 (0.94, 1.04) | 0.69 |
| ***Pooled***^4^ | **0.96 (0.89, 1.04)** | **0.92 (0.85, 1.00)** | **0.94 (0.87, 1.02)** | **0.98 (0.90, 1.06)** | **0.60** | **0.98 (0.95, 1.01)** | **0.21** |
| **Valine** |  |  |  |  |  |  |  |
| NHS | 0.99 (0.89, 1.10) | 0.94 (0.84, 1.04) | 0.96 (0.86, 1.07) | 0.96 (0.86, 1.07) | 0.39 | 0.97 (0.93, 1.01) | 0.11 |
| NHSII | 0.98 (0.76, 1.27) | 1.00 (0.78, 1.29) | 0.80 (0.61, 1.05) | 1.05 (0.82, 1.35) | 0.97 | 1.00 (0.91, 1.10) | 0.98 |
| HPFS | 0.95 (0.83, 1.09) | 0.87 (0.76, 1.00) | 0.95 (0.83, 1.08) | 0.94 (0.82, 1.08) | 0.42 | 0.99 (0.94, 1.04) | 0.57 |
| ***Pooled***^4^ | **0.98 (0.90, 1.06)** | **0.92 (0.85, 1.00)** | **0.94 (0.87, 1.02)** | **0.96 (0.89, 1.04)** | **0.27** | **0.98 (0.95, 1.01)** | **0.13** |
| **Histidine** |  |  |  |  |  |  |  |
| NHS | 0.96 (0.86, 1.06) | 0.89 (0.79, 0.99) | 0.98 (0.88, 1.09) | 0.99 (0.89, 1.10) | 0.94 | 0.98 (0.94, 1.03) | 0.43 |
| NHSII | 0.99 (0.77, 1.29) | 0.89 (0.68, 1.16) | 1.03 (0.80, 1.33) | 0.99 (0.77, 1.28) | 0.96 | 0.99 (0.90, 1.09) | 0.82 |
| HPFS | 0.87 (0.76, 0.99) | 0.89 (0.77, 1.02) | 0.96 (0.83, 1.09) | 1.01 (0.88, 1.15) | 0.60 | 1.01 (0.96, 1.06) | 0.78 |
| ***Pooled***^4^ | **0.93 (0.86, 1.01)** | **0.89 (0.82, 0.96)** | **0.97 (0.90, 1.05)** | **0.99 (0.92, 1.07)** | **0.75** | **0.99 (0.96, 1.02)** | **0.63** |
| **Lysine** |  |  |  |  |  |  |  |
| NHS | 0.97 (0.87, 1.07) | 0.92 (0.83, 1.03) | 0.95 (0.85, 1.06) | 0.99 (0.89, 1.10) | 0.82 | 0.98 (0.94, 1.03) | 0.44 |
| NHSII | 1.05 (0.81, 1.36) | 1.04 (0.80, 1.34) | 0.89 (0.68, 1.17) | 1.09 (0.85, 1.40) | 0.77 | 1.00 (0.91, 1.10) | 0.99 |
| HPFS | 0.98 (0.86, 1.12) | 0.89 (0.77, 1.02) | 0.99 (0.86, 1.13) | 1.04 (0.90, 1.19) | 0.59 | 1.00 (0.95, 1.06) | 0.86 |
| ***Pooled***^4^ | **0.98 (0.90, 1.06)** | **0.92 (0.85, 1.00)** | **0.96 (0.88, 1.04)** | **1.01 (0.94, 1.10)** | **0.75** | **0.99 (0.96, 1.02)** | **0.64** |
| **Methionine** |  |  |  |  |  |  |  |
| NHS | 0.95 (0.85, 1.06) | 0.88 (0.79, 0.98) | 0.96 (0.86, 1.06) | 0.97 (0.87, 1.08) | 0.68 | 0.98 (0.94, 1.02) | 0.29 |
| NHSII | 1.11 (0.86, 1.43) | 1.06 (0.82, 1.38) | 0.87 (0.66, 1.14) | 1.06 (0.82, 1.36) | 0.83 | 1.00 (0.90, 1.10) | 0.97 |
| HPFS | 0.95 (0.83, 1.09) | 0.92 (0.80, 1.05) | 0.94 (0.82, 1.08) | 1.02 (0.89, 1.17) | 0.77 | 1.00 (0.95, 1.05) | 0.97 |
| ***Pooled***^4^ | **0.97 (0.89, 1.05)** | **0.91 (0.84, 0.99)** | **0.94 (0.87, 1.02)** | **1.00 (0.92, 1.08)** | **0.89** | **0.99 (0.96, 1.02)** | **0.42** |
| **Phenylalanine** |  |  |  |  |  |  |  |
| NHS | 0.96 (0.86, 1.07) | 0.92 (0.83, 1.03) | 0.93 (0.84, 1.04) | 0.97 (0.87, 1.07) | 0.48 | 0.96 (0.93, 1.01) | 0.09 |
| NHSII | 0.91 (0.71, 1.18) | 0.99 (0.77, 1.27) | 0.71 (0.54, 0.93) | 0.98 (0.77, 1.26) | 0.55 | 0.98 (0.89, 1.08) | 0.74 |
| HPFS | 0.87 (0.76, 1.00) | 0.87 (0.76, 0.99) | 0.89 (0.78, 1.02) | 0.94 (0.82, 1.08) | 0.45 | 0.99 (0.94, 1.04) | 0.65 |
| ***Pooled***^4^ | **0.92 (0.85, 1.00)** | **0.91 (0.84, 0.98)** | **0.90 (0.83, 0.97)** | **0.96 (0.88, 1.04)** | **0.24** | **0.97 (0.95, 1.01)** | **0.11** |
| **Threonine** |  |  |  |  |  |  |  |
| NHS | 0.98 (0.89, 1.10) | 0.89 (0.80, 1.00) | 0.99 (0.89, 1.10) | 0.99 (0.89, 1.11) | 0.98 | 0.99 (0.94, 1.03) | 0.50 |
| NHSII | 1.11 (0.86, 1.44) | 1.06 (0.82, 1.37) | 0.84 (0.64, 1.11) | 1.10 (0.85, 1.41) | 0.97 | 0.99 (0.90, 1.09) | 0.86 |
| HPFS | 0.94 (0.82, 1.08) | 0.90 (0.78, 1.04) | 0.97 (0.85, 1.11) | 1.00 (0.87, 1.14) | 0.89 | 1.00 (0.95, 1.05) | 0.96 |
| ***Pooled***^4^ | **0.98 (0.91, 1.06)** | **0.91 (0.84, 0.99)** | **0.97 (0.89, 1.05)** | **1.01 (0.93, 1.09)** | **0.90** | **0.99 (0.96, 1.02)** | **0.56** |
| **Tryptophan** |  |  |  |  |  |  |  |
| NHS | 1.06 (0.95, 1.18) | 0.93 (0.83, 1.03) | 1.01 (0.91, 1.13) | 1.00 (0.90, 1.12) | 0.85 | 0.98 (0.94, 1.02) | 0.38 |
| NHSII | 1.22 (0.95, 1.58) | 1.02 (0.79, 1.33) | 0.86 (0.65, 1.14) | 1.15 (0.89, 1.48) | 0.82 | 1.00 (0.91, 1.10) | 1.00 |
| HPFS | 0.88 (0.77, 1.01) | 0.88 (0.76, 1.01) | 0.92 (0.80, 1.05) | 0.95 (0.83, 1.09) | 0.61 | 0.99 (0.94, 1.04) | 0.72 |
| ***Pooled***^4^ | **1.01 (0.93, 1.09)** | **0.92 (0.84, 1.00)** | **0.96 (0.89, 1.05)** | **1.00 (0.92, 1.08)** | **0.70** | **0.99 (0.96, 1.02)** | **0.40** |
| **Alanine** |  |  |  |  |  |  |  |
| NHS | 0.95 (0.86, 1.06) | 0.91 (0.82, 1.01) | 0.99 (0.89, 1.11) | 0.99 (0.89, 1.11) | 0.82 | 1.00 (0.96, 1.04) | 0.91 |
| NHSII | 0.85 (0.66, 1.11) | 0.87 (0.67, 1.13) | 0.95 (0.73, 1.22) | 0.91 (0.71, 1.17) | 0.67 | 0.98 (0.89, 1.08) | 0.65 |
| HPFS | 0.87 (0.76, 1.00) | 0.89 (0.78, 1.03) | 0.97 (0.85, 1.12) | 1.01 (0.88, 1.15) | 0.52 | 1.01 (0.96, 1.06) | 0.66 |
| ***Pooled***^4^ | **0.92 (0.84, 0.99)** | **0.90 (0.83, 0.98)** | **0.98 (0.91, 1.06)** | **0.99 (0.91, 1.07)** | **0.66** | **1.00 (0.97, 1.03)** | **0.97** |
| **Arginine** |  |  |  |  |  |  |  |
| NHS | 0.94 (0.84, 1.04) | 0.95 (0.85, 1.05) | 0.94 (0.84, 1.05) | 1.00 (0.90, 1.11) | 0.93 | 1.00 (0.95, 1.04) | 0.91 |
| NHSII | 0.91 (0.70, 1.18) | 0.89 (0.68, 1.14) | 0.84 (0.65, 1.09) | 0.94 (0.73, 1.20) | 0.54 | 0.96 (0.87, 1.06) | 0.38 |
| HPFS | 0.90 (0.79, 1.03) | 0.90 (0.79, 1.04) | 1.02 (0.89, 1.17) | 0.98 (0.86, 1.13) | 0.74 | 1.02 (0.97, 1.07) | 0.53 |
| ***Pooled***^4^ | **0.92 (0.85, 1.00)** | **0.93 (0.85, 1.00)** | **0.96 (0.88, 1.04)** | **0.99 (0.91, 1.07)** | **0.93** | **1.00 (0.97, 1.03)** | **0.98** |
| **Asparagine** |  |  |  |  |  |  |  |
| NHS | 0.96 (0.86, 1.07) | 0.95 (0.86, 1.06) | 0.94 (0.85, 1.05) | 0.97 (0.87, 1.08) | 0.61 | 0.98 (0.94, 1.02) | 0.32 |
| NHSII | 1.00 (0.78, 1.28) | 0.93 (0.72, 1.20) | 0.72 (0.55, 0.95) | 0.99 (0.77, 1.26) | 0.46 | 0.98 (0.89, 1.07) | 0.61 |
| HPFS | 0.86 (0.75, 0.98) | 0.88 (0.77, 1.01) | 0.90 (0.78, 1.03) | 0.93 (0.81, 1.06) | 0.45 | 0.99 (0.94, 1.04) | 0.73 |
| ***Pooled***^4^ | **0.93 (0.85, 1.00)** | **0.93 (0.85, 1.00)** | **0.91 (0.83, 0.98)** | **0.96 (0.89, 1.04)** | **0.28** | **0.98 (0.95, 1.01)** | **0.27** |
| **Aspartic acid** |  |  |  |  |  |  |  |
| NHS | 0.94 (0.85, 1.05) | 0.88 (0.78, 0.98) | 0.96 (0.86, 1.07) | 1.00 (0.90, 1.12) | 0.67 | 1.00 (0.96, 1.05) | 0.94 |
| NHSII | 0.88 (0.68, 1.14) | 0.84 (0.64, 1.09) | 0.84 (0.65, 1.09) | 0.98 (0.77, 1.26) | 0.93 | 0.98 (0.89, 1.08) | 0.65 |
| HPFS | 0.95 (0.83, 1.09) | 0.90 (0.78, 1.03) | 0.92 (0.80, 1.06) | 1.07 (0.94, 1.22) | 0.39 | 1.02 (0.97, 1.07) | 0.50 |
| ***Pooled***^4^ | **0.94 (0.87, 1.02)** | **0.88 (0.81, 0.96)** | **0.94 (0.86, 1.01)** | **1.02 (0.95, 1.11)** | **0.41** | **1.01 (0.97, 1.04)** | **0.75** |
| **Cystine** |  |  |  |  |  |  |  |
| NHS | 0.94 (0.85, 1.04) | 0.89 (0.80, 0.99) | 0.89 (0.80, 0.99) | 0.92 (0.83, 1.03) | 0.09 | 0.96 (0.93, 1.00) | 0.07 |
| NHSII | 1.02 (0.79, 1.30) | 0.91 (0.70, 1.17) | 0.87 (0.67, 1.13) | 0.98 (0.76, 1.26) | 0.61 | 0.98 (0.89, 1.07) | 0.61 |
| HPFS | 0.82 (0.71, 0.94) | 0.87 (0.76, 1.00) | 0.96 (0.84, 1.10) | 0.89 (0.78, 1.03) | 0.47 | 0.99 (0.94, 1.04) | 0.68 |
| ***Pooled***^4^ | **0.90 (0.84, 0.98)** | **0.89 (0.82, 0.96)** | **0.91 (0.84, 0.99)** | **0.92 (0.85, 0.99)** | **0.08** | **0.97 (0.95, 1.00)** | **0.08** |
| **Glutamine** |  |  |  |  |  |  |  |
| NHS | 0.89 (0.80, 0.99) | 0.92 (0.83, 1.02) | 0.89 (0.80, 0.99) | 0.85 (0.76, 0.95) | 0.01 | 0.93 (0.90, 0.97) | 0.00 |
| NHSII | 0.92 (0.72, 1.17) | 0.89 (0.69, 1.14) | 0.93 (0.73, 1.20) | 0.78 (0.60, 1.02) | 0.11 | 0.93 (0.85, 1.02) | 0.13 |
| HPFS | 0.98 (0.86, 1.11) | 0.82 (0.71, 0.94) | 0.92 (0.80, 1.05) | 0.91 (0.80, 1.05) | 0.10 | 0.94 (0.89, 0.99) | 0.02 |
| ***Pooled***^4^ | **0.92 (0.85, 1.00)** | **0.88 (0.81, 0.95)** | **0.90 (0.83, 0.98)** | **0.87 (0.80, 0.94)** | **<0.001** | **0.94 (0.91, 0.97)** | **<0.001** |
| **Glutamic acid** |  |  |  |  |  |  |  |
| NHS | 0.94 (0.84, 1.04) | 0.89 (0.80, 0.99) | 0.92 (0.83, 1.02) | 0.97 (0.87, 1.08) | 0.60 | 0.98 (0.94, 1.02) | 0.35 |
| NHSII | 1.10 (0.86, 1.42) | 0.92 (0.70, 1.20) | 0.91 (0.70, 1.19) | 1.10 (0.85, 1.41) | 0.75 | 1.00 (0.91, 1.11) | 0.96 |
| HPFS | 0.87 (0.76, 0.99) | 0.90 (0.78, 1.03) | 0.91 (0.80, 1.05) | 0.97 (0.84, 1.10) | 0.87 | 1.00 (0.95, 1.05) | 0.90 |
| ***Pooled***^4^ | **0.93 (0.86, 1.00)** | **0.89 (0.82, 0.97)** | **0.92 (0.85, 0.99)** | **0.98 (0.91, 1.06)** | **0.71** | **0.99 (0.96, 1.02)** | **0.46** |
| **Glycine** |  |  |  |  |  |  |  |
| NHS | 0.92 (0.83, 1.03) | 0.96 (0.86, 1.07) | 0.98 (0.88, 1.09) | 1.00 (0.90, 1.12) | 0.59 | 1.01 (0.97, 1.05) | 0.72 |
| NHSII | 0.92 (0.71, 1.20) | 0.91 (0.70, 1.19) | 0.98 (0.76, 1.27) | 0.96 (0.74, 1.23) | 0.85 | 0.98 (0.89, 1.08) | 0.64 |
| HPFS | 0.83 (0.72, 0.95) | 0.87 (0.76, 1.00) | 1.01 (0.88, 1.15) | 1.00 (0.88, 1.15) | 0.30 | 1.02 (0.96, 1.07) | 0.57 |
| ***Pooled***^4^ | **0.89 (0.82, 0.96)** | **0.92 (0.85, 1.00)** | **0.99 (0.92, 1.07)** | **1.00 (0.92, 1.08)** | **0.32** | **1.01 (0.98, 1.04)** | **0.65** |
| **Proline** |  |  |  |  |  |  |  |
| NHS | 0.96 (0.87, 1.07) | 0.83 (0.74, 0.93) | 0.97 (0.88, 1.08) | 0.88 (0.79, 0.98) | 0.04 | 0.94 (0.91, 0.98) | 0.01 |
| NHSII | 1.19 (0.93, 1.52) | 0.95 (0.73, 1.23) | 0.89 (0.68, 1.16) | 1.12 (0.87, 1.45) | 0.87 | 1.02 (0.93, 1.12) | 0.69 |
| HPFS | 0.93 (0.82, 1.07) | 0.94 (0.83, 1.08) | 0.81 (0.71, 0.94) | 0.88 (0.76, 1.01) | 0.02 | 0.96 (0.91, 1.01) | 0.10 |
| ***Pooled***^4^ | **0.97 (0.90, 1.05)** | **0.88 (0.81, 0.96)** | **0.91 (0.84, 0.99)** | **0.90 (0.83, 0.98)** | **0.004** | **0.96 (0.93, 0.99)** | **0.004** |
| **Serine** |  |  |  |  |  |  |  |
| NHS | 0.94 (0.84, 1.04) | 0.92 (0.83, 1.02) | 0.93 (0.83, 1.03) | 0.94 (0.85, 1.05) | 0.32 | 0.96 (0.92, 1.00) | 0.07 |
| NHSII | 1.01 (0.79, 1.29) | 0.93 (0.72, 1.20) | 0.88 (0.68, 1.14) | 0.94 (0.73, 1.21) | 0.49 | 0.99 (0.90, 1.09) | 0.90 |
| HPFS | 0.90 (0.79, 1.03) | 0.89 (0.78, 1.02) | 0.90 (0.79, 1.04) | 0.94 (0.82, 1.07) | 0.37 | 0.99 (0.94, 1.04) | 0.57 |
| ***Pooled***^4^ | **0.93 (0.86, 1.01)** | **0.91 (0.84, 0.99)** | **0.91 (0.84, 0.99)** | **0.94 (0.87, 1.02)** | **0.13** | **0.97 (0.94, 1.00)** | **0.08** |
| **Tyrosine** |  |  |  |  |  |  |  |
| NHS | 0.95 (0.85, 1.05) | 0.95 (0.85, 1.06) | 0.95 (0.85, 1.06) | 0.93 (0.84, 1.04) | 0.26 | 0.97 (0.93, 1.01) | 0.10 |
| NHSII | 0.99 (0.76, 1.28) | 1.08 (0.84, 1.38) | 0.82 (0.63, 1.08) | 1.05 (0.82, 1.35) | 0.93 | 1.00 (0.91, 1.10) | 0.98 |
| HPFS | 0.97 (0.84, 1.10) | 0.86 (0.75, 0.99) | 0.95 (0.83, 1.09) | 0.95 (0.83, 1.09) | 0.48 | 0.98 (0.93, 1.03) | 0.52 |
| ***Pooled***^4^ | **0.96 (0.88, 1.04)** | **0.93 (0.86, 1.01)** | **0.94 (0.87, 1.02)** | **0.95 (0.88, 1.03)** | **0.24** | **0.98 (0.95, 1.01)** | **0.11** |
| **Hydroxyproline** |  |  |  |  |  |  |  |
| NHS | 0.98 (0.88, 1.09) | 1.01 (0.91, 1.13) | 1.03 (0.93, 1.15) | 1.14 (1.03, 1.27) | 0.004 | 1.06 (1.01, 1.10) | 0.02 |
| NHSII | 0.89 (0.67, 1.17) | 1.12 (0.86, 1.45) | 0.92 (0.71, 1.21) | 1.02 (0.79, 1.32) | 0.92 | 1.02 (0.93, 1.12) | 0.69 |
| HPFS | 1.04 (0.91, 1.20) | 1.10 (0.95, 1.26) | 1.03 (0.90, 1.19) | 1.20 (1.04, 1.38) | 0.01 | 1.05 (1.00, 1.11) | 0.05 |
| ***Pooled***^4^ | **0.99 (0.91, 1.07)** | **1.05 (0.97, 1.14)** | **1.02 (0.94, 1.11)** | **1.15 (1.06, 1.24)** | **<0.001** | **1.05 (1.02, 1.08)** | **0.002** |
| **Taurine** |  |  |  |  |  |  |  |
| NHS | 1.00 (0.90, 1.11) | 0.93 (0.83, 1.03) | 0.96 (0.86, 1.07) | 1.02 (0.92, 1.14) | 0.81 | 0.99 (0.95, 1.03) | 0.62 |
| NHSII | 0.97 (0.75, 1.25) | 0.80 (0.61, 1.05) | 0.88 (0.69, 1.14) | 1.01 (0.79, 1.29) | 0.99 | 1.00 (0.91, 1.12) | 0.93 |
| HPFS | 0.92 (0.80, 1.05) | 0.94 (0.82, 1.07) | 0.99 (0.86, 1.13) | 1.03 (0.90, 1.17) | 0.42 | 1.01 (0.96, 1.07) | 0.57 |
| ***Pooled***^4^ | **0.97 (0.89, 1.05)** | **0.92 (0.85, 1.00)** | **0.96 (0.88, 1.04)** | **1.02 (0.95, 1.11)** | **0.47** | **1.00 (0.97, 1.03)** | **0.98** |

1. Analyses were stratified by age (in years) and follow-up intervals, and adjusted for ethnicity (white, non-white), marital status (married, widowed, divorced/separated, unknown), smoking status (never, past, current: 1–14, 15–24, ≥25 cigarettes/day, unknown status or cigarette number), alcohol intake (never drinkers, 0.1-4.9, 5.0-9.9, 10.0-14.9, 15.0+ g/d), physical activity (<3, 3–9, 9–18, 18–27, 27–42, ≥42 metabolic equivalents/week, unknown), menopausal status and postmenopausal hormone use (premenopausal, postmenopausal: never, past, current users, NHS and NHSII only), oral contraception use (never, past, current user, NHSII only), multivitamin use (no, yes), aspirin use (no, yes), body mass index (<23, 23–24.9, 25–29.9, 30–34.9, ≥35 kg/m2, missing), baseline history of hypertension (no, yes), baseline hypercholesteremia (no, yes), baseline diabetes (no, yes), and energy intake (fifths of intake).
2. Linear trends estimated by fitting the median values in each fifth of intake as a pseudo-continuous variable in the Cox regression.
3. P-values estimated by fitting the continuous intakes of dietary amino acids in the Cox regression.
4. Results from NHS, NHSII and HPFS were pooled using a fixed-effects meta-analysis.

Supplementary table 8: Hazard ratios (95% confidence intervals) for ischemic stroke (3058 cases) by fifths and per SD difference of energy-adjusted grams of dietary amino acids, across different adjustment models.

|  | **HR (95% CI), fifths of intake compared to bottom fifth^1^** | | | |  | **HR (95% CI)**  **per SD higher intake^1^** | ***p*-value^3^** |
| --- | --- | --- | --- | --- | --- | --- | --- |
| **Amino acids** | **Q2** | **Q3** | **Q4** | **Q5** | ***p*-trend^2^** |  |  |
| **Isoleucine** |  |  |  |  |  |  |  |
| Age-adjusted | 0.90 (0.80, 1.00) | 0.86 (0.77, 0.96) | 0.87 (0.78, 0.98) | 0.96 (0.86, 1.07) | 0.38 | 0.98 (0.94, 1.03) | 0.47 |
| Multivariable 1 | 0.90 (0.81, 1.01) | 0.86 (0.77, 0.97) | 0.87 (0.78, 0.97) | 0.91 (0.81, 1.01) | 0.07 | 0.96 (0.92, 1.00) | 0.06 |
| Multivariable 2 | 0.91 (0.81, 1.01) | 0.87 (0.77, 0.97) | 0.88 (0.78, 0.98) | 0.92 (0.82, 1.03) | 0.13 | 0.96 (0.92, 1.01) | 0.10 |
| **Leucine** |  |  |  |  |  |  |  |
| Age-adjusted | 0.91 (0.81, 1.01) | 0.87 (0.78, 0.97) | 0.87 (0.78, 0.98) | 0.98 (0.88, 1.09) | 0.48 | 0.98 (0.94, 1.02) | 0.41 |
| Multivariable 1 | 0.92 (0.82, 1.03) | 0.87 (0.78, 0.98) | 0.87 (0.78, 0.97) | 0.92 (0.83, 1.03) | 0.10 | 0.96 (0.92, 1.00) | 0.04 |
| Multivariable 2 | 0.92 (0.83, 1.03) | 0.88 (0.79, 0.99) | 0.87 (0.78, 0.98) | 0.93 (0.83, 1.05) | 0.17 | 0.96 (0.92, 1.00) | 0.08 |
| **Valine** |  |  |  |  |  |  |  |
| Age-adjusted | 0.91 (0.81, 1.01) | 0.87 (0.78, 0.97) | 0.86 (0.77, 0.96) | 0.95 (0.85, 1.05) | 0.17 | 0.97 (0.93, 1.01) | 0.20 |
| Multivariable 1 | 0.92 (0.83, 1.03) | 0.88 (0.79, 0.98) | 0.86 (0.77, 0.96) | 0.90 (0.81, 1.01) | 0.04 | 0.95 (0.91, 1.00) | 0.03 |
| Multivariable 2 | 0.93 (0.83, 1.03) | 0.88 (0.79, 0.99) | 0.87 (0.77, 0.97) | 0.92 (0.82, 1.03) | 0.08 | 0.96 (0.92, 1.00) | 0.06 |
| **Histidine** |  |  |  |  |  |  |  |
| Age-adjusted | 0.88 (0.78, 0.98) | 0.85 (0.76, 0.95) | 0.90 (0.81, 1.01) | 1.03 (0.93, 1.15) | 0.52 | 1.01 (0.96, 1.05) | 0.77 |
| Multivariable 1 | 0.88 (0.79, 0.99) | 0.85 (0.76, 0.95) | 0.89 (0.80, 1.00) | 0.95 (0.85, 1.06) | 0.43 | 0.97 (0.93, 1.01) | 0.13 |
| Multivariable 2 | 0.89 (0.79, 0.99) | 0.85 (0.76, 0.95) | 0.90 (0.80, 1.00) | 0.96 (0.86, 1.08) | 0.60 | 0.97 (0.93, 1.02) | 0.20 |
| **Lysine** |  |  |  |  |  |  |  |
| Age-adjusted | 0.89 (0.80, 0.99) | 0.87 (0.78, 0.98) | 0.91 (0.81, 1.02) | 0.99 (0.89, 1.11) | 0.97 | 1.00 (0.96, 1.05) | 0.90 |
| Multivariable 1 | 0.89 (0.80, 1.00) | 0.86 (0.77, 0.97) | 0.89 (0.80, 1.00) | 0.92 (0.82, 1.02) | 0.15 | 0.96 (0.92, 1.01) | 0.11 |
| Multivariable 2 | 0.89 (0.80, 1.00) | 0.87 (0.77, 0.97) | 0.90 (0.80, 1.00) | 0.93 (0.83, 1.04) | 0.24 | 0.97 (0.93, 1.01) | 0.17 |
| **Methionine** |  |  |  |  |  |  |  |
| Age-adjusted | 0.90 (0.81, 1.01) | 0.89 (0.79, 0.99) | 0.88 (0.78, 0.98) | 1.01 (0.91, 1.12) | 0.88 | 0.99 (0.95, 1.04) | 0.77 |
| Multivariable 1 | 0.90 (0.81, 1.01) | 0.88 (0.79, 0.99) | 0.86 (0.77, 0.97) | 0.93 (0.84, 1.04) | 0.16 | 0.96 (0.92, 1.00) | 0.06 |
| Multivariable 2 | 0.91 (0.81, 1.01) | 0.89 (0.79, 0.99) | 0.87 (0.78, 0.98) | 0.95 (0.85, 1.06) | 0.27 | 0.96 (0.92, 1.01) | 0.11 |
| **Phenylalanine** |  |  |  |  |  |  |  |
| Age-adjusted | 0.87 (0.78, 0.97) | 0.84 (0.75, 0.94) | 0.85 (0.76, 0.95) | 0.95 (0.85, 1.06) | 0.25 | 0.97 (0.93, 1.01) | 0.18 |
| Multivariable 1 | 0.88 (0.79, 0.99) | 0.85 (0.76, 0.95) | 0.85 (0.76, 0.96) | 0.91 (0.81, 1.01) | 0.07 | 0.95 (0.91, 0.99) | 0.03 |
| Multivariable 2 | 0.89 (0.79, 0.99) | 0.86 (0.77, 0.96) | 0.86 (0.77, 0.97) | 0.92 (0.82, 1.03) | 0.14 | 0.96 (0.92, 1.00) | 0.06 |
| **Threonine** |  |  |  |  |  |  |  |
| Age-adjusted | 0.93 (0.84, 1.04) | 0.85 (0.76, 0.95) | 0.94 (0.84, 1.04) | 1.02 (0.92, 1.14) | 0.75 | 1.00 (0.96, 1.04) | 0.95 |
| Multivariable 1 | 0.94 (0.84, 1.05) | 0.86 (0.76, 0.96) | 0.93 (0.83, 1.04) | 0.95 (0.85, 1.06) | 0.37 | 0.96 (0.92, 1.01) | 0.11 |
| Multivariable 2 | 0.95 (0.85, 1.06) | 0.86 (0.77, 0.97) | 0.93 (0.83, 1.05) | 0.97 (0.86, 1.08) | 0.56 | 0.97 (0.93, 1.01) | 0.18 |
| **Tryptophan** |  |  |  |  |  |  |  |
| Age-adjusted | 0.96 (0.86, 1.08) | 0.83 (0.74, 0.93) | 0.91 (0.81, 1.02) | 0.98 (0.88, 1.09) | 0.42 | 0.98 (0.94, 1.03) | 0.48 |
| Multivariable 1 | 0.98 (0.88, 1.10) | 0.84 (0.75, 0.95) | 0.92 (0.82, 1.03) | 0.93 (0.83, 1.04) | 0.11 | 0.96 (0.92, 1.00) | 0.07 |
| Multivariable 2 | 0.99 (0.88, 1.10) | 0.85 (0.76, 0.95) | 0.93 (0.83, 1.04) | 0.94 (0.84, 1.06) | 0.20 | 0.97 (0.92, 1.01) | 0.13 |
| **Alanine** |  |  |  |  |  |  |  |
| Age-adjusted | 0.85 (0.76, 0.95) | 0.88 (0.78, 0.98) | 0.92 (0.82, 1.02) | 1.01 (0.91, 1.13) | 0.55 | 1.01 (0.97, 1.06) | 0.59 |
| Multivariable 1 | 0.86 (0.77, 0.96) | 0.88 (0.78, 0.98) | 0.90 (0.81, 1.01) | 0.94 (0.84, 1.05) | 0.46 | 0.97 (0.93, 1.02) | 0.24 |
| Multivariable 2 | 0.86 (0.77, 0.96) | 0.88 (0.79, 0.99) | 0.91 (0.81, 1.02) | 0.95 (0.85, 1.07) | 0.68 | 0.98 (0.94, 1.02) | 0.36 |
| **Arginine** |  |  |  |  |  |  |  |
| Age-adjusted | 0.86 (0.77, 0.96) | 0.85 (0.76, 0.95) | 0.89 (0.80, 1.00) | 0.98 (0.88, 1.09) | 0.82 | 1.00 (0.96, 1.05) | 0.84 |
| Multivariable 1 | 0.87 (0.78, 0.97) | 0.85 (0.76, 0.95) | 0.89 (0.80, 0.99) | 0.92 (0.82, 1.02) | 0.19 | 0.97 (0.93, 1.02) | 0.22 |
| Multivariable 2 | 0.87 (0.78, 0.98) | 0.86 (0.77, 0.96) | 0.90 (0.80, 1.01) | 0.93 (0.83, 1.05) | 0.37 | 0.98 (0.94, 1.03) | 0.39 |
| **Asparagine** |  |  |  |  |  |  |  |
| Age-adjusted | 0.87 (0.77, 0.97) | 0.83 (0.75, 0.93) | 0.86 (0.77, 0.96) | 0.93 (0.84, 1.04) | 0.20 | 0.97 (0.93, 1.02) | 0.23 |
| Multivariable 1 | 0.89 (0.79, 0.99) | 0.85 (0.76, 0.96) | 0.89 (0.79, 0.99) | 0.91 (0.82, 1.02) | 0.14 | 0.96 (0.92, 1.01) | 0.10 |
| Multivariable 2 | 0.89 (0.80, 1.00) | 0.86 (0.77, 0.97) | 0.90 (0.80, 1.01) | 0.93 (0.83, 1.05) | 0.31 | 0.97 (0.93, 1.02) | 0.23 |
| **Aspartic acid** |  |  |  |  |  |  |  |
| Age-adjusted | 0.90 (0.80, 1.00) | 0.85 (0.76, 0.96) | 0.91 (0.82, 1.02) | 1.06 (0.95, 1.18) | 0.25 | 1.03 (0.98, 1.07) | 0.23 |
| Multivariable 1 | 0.89 (0.80, 1.00) | 0.84 (0.75, 0.94) | 0.89 (0.79, 0.99) | 0.95 (0.85, 1.07) | 0.52 | 0.98 (0.93, 1.02) | 0.32 |
| Multivariable 2 | 0.90 (0.80, 1.01) | 0.85 (0.76, 0.95) | 0.90 (0.80, 1.01) | 0.97 (0.87, 1.09) | 0.80 | 0.98 (0.94, 1.03) | 0.51 |
| **Cystine** |  |  |  |  |  |  |  |
| Age-adjusted | 0.89 (0.80, 0.99) | 0.81 (0.72, 0.90) | 0.87 (0.78, 0.97) | 0.90 (0.81, 1.01) | 0.05 | 0.97 (0.93, 1.01) | 0.12 |
| Multivariable 1 | 0.91 (0.82, 1.02) | 0.83 (0.75, 0.93) | 0.89 (0.80, 1.00) | 0.90 (0.80, 1.00) | 0.06 | 0.97 (0.93, 1.01) | 0.11 |
| Multivariable 2 | 0.92 (0.82, 1.02) | 0.84 (0.75, 0.94) | 0.90 (0.81, 1.01) | 0.91 (0.81, 1.02) | 0.13 | 0.97 (0.93, 1.02) | 0.22 |
| **Glutamine** |  |  |  |  |  |  |  |
| Age-adjusted | 0.84 (0.76, 0.94) | 0.79 (0.71, 0.88) | 0.79 (0.71, 0.89) | 0.82 (0.73, 0.91) | <0.001 | 0.92 (0.88, 0.96) | <0.001 |
| Multivariable 1 | 0.88 (0.79, 0.98) | 0.84 (0.75, 0.93) | 0.85 (0.76, 0.95) | 0.86 (0.77, 0.97) | 0.007 | 0.94 (0.90, 0.98) | 0.004 |
| Multivariable 2 | 0.88 (0.79, 0.98) | 0.84 (0.75, 0.94) | 0.85 (0.76, 0.96) | 0.87 (0.78, 0.98) | 0.02 | 0.94 (0.90, 0.99) | 0.01 |
| **Glutamic acid** |  |  |  |  |  |  |  |
| Age-adjusted | 0.86 (0.77, 0.96) | 0.83 (0.74, 0.93) | 0.83 (0.75, 0.93) | 0.96 (0.87, 1.07) | 0.48 | 0.99 (0.95, 1.04) | 0.78 |
| Multivariable 1 | 0.86 (0.77, 0.96) | 0.83 (0.74, 0.93) | 0.82 (0.73, 0.92) | 0.90 (0.80, 1.00) | 0.05 | 0.96 (0.92, 1.00) | 0.06 |
| Multivariable 2 | 0.86 (0.77, 0.97) | 0.84 (0.75, 0.94) | 0.83 (0.74, 0.93) | 0.91 (0.81, 1.02) | 0.10 | 0.96 (0.92, 1.01) | 0.11 |
| **Glycine** |  |  |  |  |  |  |  |
| Age-adjusted | 0.88 (0.79, 0.99) | 0.89 (0.80, 1.00) | 0.96 (0.86, 1.07) | 1.05 (0.94, 1.17) | 0.22 | 1.02 (0.98, 1.07) | 0.33 |
| Multivariable 1 | 0.88 (0.79, 0.99) | 0.89 (0.80, 1.00) | 0.94 (0.84, 1.05) | 0.96 (0.86, 1.07) | 0.80 | 0.98 (0.94, 1.02) | 0.37 |
| Multivariable 2 | 0.89 (0.79, 0.99) | 0.90 (0.80, 1.01) | 0.95 (0.85, 1.06) | 0.98 (0.87, 1.09) | 0.95 | 0.99 (0.94, 1.03) | 0.52 |
| **Proline** |  |  |  |  |  |  |  |
| Age-adjusted | 0.92 (0.82, 1.02) | 0.83 (0.74, 0.93) | 0.84 (0.75, 0.94) | 0.88 (0.79, 0.98) | 0.005 | 0.95 (0.91, 0.99) | 0.015 |
| Multivariable 1 | 0.94 (0.84, 1.04) | 0.84 (0.75, 0.94) | 0.84 (0.75, 0.95) | 0.85 (0.76, 0.96) | 0.002 | 0.94 (0.90, 0.98) | 0.005 |
| Multivariable 2 | 0.94 (0.84, 1.05) | 0.84 (0.75, 0.94) | 0.85 (0.76, 0.95) | 0.86 (0.77, 0.97) | 0.003 | 0.94 (0.91, 0.99) | 0.009 |
| **Serine** |  |  |  |  |  |  |  |
| Age-adjusted | 0.86 (0.77, 0.96) | 0.84 (0.75, 0.94) | 0.83 (0.74, 0.93) | 0.93 (0.84, 1.04) | 0.13 | 0.97 (0.93, 1.01) | 0.14 |
| Multivariable 1 | 0.88 (0.79, 0.98) | 0.86 (0.77, 0.96) | 0.84 (0.75, 0.94) | 0.90 (0.80, 1.01) | 0.04 | 0.95 (0.91, 0.99) | 0.03 |
| Multivariable 2 | 0.88 (0.79, 0.99) | 0.87 (0.78, 0.97) | 0.84 (0.75, 0.95) | 0.91 (0.81, 1.02) | 0.08 | 0.96 (0.92, 1.00) | 0.06 |
| **Tyrosine** |  |  |  |  |  |  |  |
| Age-adjusted | 0.90 (0.81, 1.00) | 0.88 (0.78, 0.98) | 0.86 (0.77, 0.96) | 0.96 (0.86, 1.07) | 0.28 | 0.97 (0.93, 1.02) | 0.22 |
| Multivariable 1 | 0.91 (0.81, 1.01) | 0.88 (0.79, 0.98) | 0.85 (0.76, 0.95) | 0.90 (0.81, 1.01) | 0.04 | 0.95 (0.91, 0.99) | 0.01 |
| Multivariable 2 | 0.91 (0.81, 1.01) | 0.88 (0.79, 0.99) | 0.85 (0.76, 0.96) | 0.91 (0.82, 1.02) | 0.07 | 0.95 (0.91, 0.99) | 0.03 |
| **Hydroxyproline** |  |  |  |  |  |  |  |
| Age-adjusted | 1.05 (0.94, 1.18) | 1.12 (1.00, 1.26) | 1.16 (1.04, 1.30) | 1.38 (1.23, 1.54) | <0.001 | 1.13 (1.08, 1.17) | <0.001 |
| Multivariable 1 | 1.00 (0.90, 1.13) | 1.04 (0.92, 1.16) | 1.04 (0.92, 1.16) | 1.11 (0.99, 1.25) | 0.04 | 1.02 (0.98, 1.07) | 0.33 |
| Multivariable 2 | 1.00 (0.89, 1.12) | 1.03 (0.92, 1.16) | 1.03 (0.91, 1.15) | 1.10 (0.98, 1.23) | 0.07 | 1.02 (0.97, 1.06) | 0.47 |
| **Taurine** |  |  |  |  |  |  |  |
| Age-adjusted | 0.91 (0.82, 1.02) | 0.90 (0.80, 1.01) | 0.90 (0.80, 1.01) | 0.97 (0.87, 1.08) | 0.52 | 1.00 (0.95, 1.04) | 0.82 |
| Multivariable 1 | 0.92 (0.82, 1.03) | 0.90 (0.81, 1.01) | 0.90 (0.80, 1.00) | 0.94 (0.84, 1.05) | 0.27 | 0.98 (0.94, 1.02) | 0.32 |
| Multivariable 2 | 0.92 (0.82, 1.03) | 0.91 (0.81, 1.02) | 0.91 (0.81, 1.02) | 0.96 (0.86, 1.08) | 0.50 | - 1. (0.94, 1.03) | 0.52 |

1. The age-adjusted model was stratified by age (in years) and follow-up intervals. The multivariable model 1 was additionally adjusted for ethnicity (white, non-white), marital status (married, widowed, divorced/separated, unknown), smoking status (never, past, current: 1–14, 15–24, ≥25 cigarettes/day, unknown status or cigarette number), alcohol intake (never drinkers, 0.1-4.9, 5.0-9.9, 10.0-14.9, 15.0+ g/d), physical activity (<3, 3–9, 9–18, 18–27, 27–42, ≥42 metabolic equivalents/week, unknown), menopausal status and postmenopausal hormone use (premenopausal, postmenopausal: never, past, current users, NHS and NHSII only), oral contraception use (never, past, current user, NHSII only), multivitamin use (no, yes), aspirin use (no, yes), body mass index (<23, 23–24.9, 25–29.9, 30–34.9, ≥35 kg/m2, missing), baseline history of hypertension (no, yes), baseline hypercholesteremia (no, yes), baseline diabetes (no, yes), and energy intake (fifths of intake), and was considered the main model in these analyses. Multivariable model 2 additionally adjusted for the Alternative Healthy Eating Index (fifths of score). Results from NHS, NHSII and HPFS were pooled using a fixed-effects meta-analysis.
2. Linear trends estimated by fitting the median values in each fifth of intake as a pseudo-continuous variable in the Cox regression.
3. P-values estimated by fitting the continuous intakes of dietary amino acids in the Cox regression.

Supplementary table 9: Hazard ratios (95% confidence intervals) for hemorrhagic stroke (872 cases) by fifths and per SD difference of energy-adjusted grams of dietary amino acids, across different adjustment models.

|  | **HR (95% CI), fifths of intake compared to bottom fifth^1^** | | | |  | **HR (95% CI)**  **per SD higher intake^1^** | ***p*-value^3^** |
| --- | --- | --- | --- | --- | --- | --- | --- |
| **Amino acids** | **Q2** | **Q3** | **Q4** | **Q5** | ***p*-trend^2^** |  |  |
| **Isoleucine** |  |  |  |  |  |  |  |
| Age-adjusted | 1.10 (0.90, 1.35) | 0.92 (0.74, 1.13) | 0.90 (0.73, 1.11) | 0.92 (0.75, 1.14) | 0.15 | 0.94 (0.87, 1.02) | 0.16 |
| Multivariable 1 | 1.17 (0.96, 1.44) | 0.98 (0.79, 1.21) | 0.97 (0.78, 1.20) | 0.97 (0.78, 1.20) | 0.34 | 0.96 (0.89, 1.04) | 0.36 |
| Multivariable 2 | 1.19 (0.97, 1.46) | 1.00 (0.81, 1.24) | 0.99 (0.80, 1.23) | 1.01 (0.81, 1.26) | 0.53 | 0.98 (0.90, 1.06) | 0.58 |
| **Leucine** |  |  |  |  |  |  |  |
| Age-adjusted | 1.11 (0.91, 1.36) | 0.90 (0.73, 1.11) | 0.97 (0.79, 1.20) | 0.92 (0.74, 1.14) | 0.24 | 0.95 (0.87, 1.02) | 0.18 |
| Multivariable 1 | 1.18 (0.96, 1.44) | 0.96 (0.78, 1.19) | 1.05 (0.85, 1.29) | 0.97 (0.78, 1.20) | 0.49 | 0.97 (0.89, 1.05) | 0.40 |
| Multivariable 2 | 1.19 (0.97, 1.46) | 0.98 (0.79, 1.22) | 1.07 (0.87, 1.33) | 1.00 (0.80, 1.25) | 0.69 | 0.98 (0.90, 1.06) | 0.61 |
| **Valine** |  |  |  |  |  |  |  |
| Age-adjusted | 1.05 (0.86, 1.29) | 0.88 (0.72, 1.09) | 0.91 (0.74, 1.12) | 0.89 (0.73, 1.10) | 0.15 | 0.94 (0.87, 1.02) | 0.13 |
| Multivariable 1 | 1.12 (0.92, 1.37) | 0.95 (0.77, 1.17) | 0.99 (0.80, 1.22) | 0.95 (0.76, 1.17) | 0.36 | 0.96 (0.89, 1.04) | 0.34 |
| Multivariable 2 | 1.14 (0.93, 1.39) | 0.97 (0.78, 1.20) | 1.01 (0.82, 1.26) | 0.98 (0.79, 1.22) | 0.57 | 0.98 (0.90, 1.06) | 0.57 |
| **Histidine** |  |  |  |  |  |  |  |
| Age-adjusted | 0.99 (0.81, 1.22) | 0.90 (0.73, 1.11) | 1.00 (0.82, 1.23) | 0.89 (0.72, 1.09) | 0.27 | 0.96 (0.88, 1.04) | 0.31 |
| Multivariable 1 | 1.04 (0.85, 1.28) | 0.95 (0.77, 1.18) | 1.07 (0.87, 1.31) | 0.92 (0.74, 1.14) | 0.46 | 0.97 (0.90, 1.06) | 0.51 |
| Multivariable 2 | 1.05 (0.86, 1.30) | 0.97 (0.78, 1.20) | 1.09 (0.88, 1.34) | 0.95 (0.76, 1.18) | 0.62 | 0.98 (0.91, 1.07) | 0.71 |
| **Lysine** |  |  |  |  |  |  |  |
| Age-adjusted | 1.02 (0.83, 1.25) | 0.94 (0.77, 1.16) | 0.96 (0.78, 1.18) | 0.91 (0.73, 1.12) | 0.27 | 0.96 (0.89, 1.04) | 0.32 |
| Multivariable 1 | 1.07 (0.88, 1.32) | 1.00 (0.81, 1.23) | 1.01 (0.82, 1.25) | 0.94 (0.76, 1.16) | 0.44 | 0.97 (0.90, 1.05) | 0.49 |
| Multivariable 2 | 1.09 (0.89, 1.33) | 1.01 (0.82, 1.25) | 1.04 (0.84, 1.28) | 0.97 (0.78, 1.21) | 0.63 | 0.98 (0.91, 1.07) | 0.71 |
| **Methionine** |  |  |  |  |  |  |  |
| Age-adjusted | 1.08 (0.89, 1.33) | 0.87 (0.70, 1.07) | 0.99 (0.81, 1.22) | 0.92 (0.75, 1.14) | 0.29 | 0.95 (0.87, 1.03) | 0.20 |
| Multivariable 1 | 1.14 (0.93, 1.40) | 0.92 (0.74, 1.15) | 1.06 (0.86, 1.30) | 0.96 (0.77, 1.19) | 0.49 | 0.96 (0.89, 1.04) | 0.36 |
| Multivariable 2 | 1.15 (0.94, 1.41) | 0.94 (0.76, 1.17) | 1.08 (0.88, 1.34) | 0.99 (0.80, 1.24) | 0.70 | 0.98 (0.90, 1.06) | 0.56 |
| **Phenylalanine** |  |  |  |  |  |  |  |
| Age-adjusted | 0.95 (0.78, 1.16) | 0.88 (0.72, 1.08) | 0.84 (0.68, 1.04) | 0.88 (0.71, 1.08) | 0.11 | 0.94 (0.87, 1.01) | 0.11 |
| Multivariable 1 | 1.01 (0.82, 1.24) | 0.95 (0.77, 1.16) | 0.91 (0.73, 1.12) | 0.93 (0.75, 1.15) | 0.30 | 0.96 (0.89, 1.04) | 0.34 |
| Multivariable 2 | 1.02 (0.83, 1.26) | 0.97 (0.78, 1.19) | 0.94 (0.75, 1.16) | 0.97 (0.78, 1.21) | 0.50 | 0.98 (0.90, 1.06) | 0.58 |
| **Threonine** |  |  |  |  |  |  |  |
| Age-adjusted | 1.06 (0.87, 1.30) | 0.92 (0.75, 1.14) | 0.99 (0.80, 1.21) | 0.88 (0.71, 1.08) | 0.16 | 0.95 (0.88, 1.03) | 0.23 |
| Multivariable 1 | 1.12 (0.91, 1.37) | 0.98 (0.80, 1.22) | 1.05 (0.85, 1.29) | 0.91 (0.73, 1.13) | 0.30 | 0.97 (0.89, 1.05) | 0.41 |
| Multivariable 2 | 1.13 (0.92, 1.39) | 1.00 (0.81, 1.24) | 1.07 (0.87, 1.33) | 0.94 (0.75, 1.18) | 0.46 | 0.98 (0.90, 1.06) | 0.63 |
| **Tryptophan** |  |  |  |  |  |  |  |
| Age-adjusted | 1.15 (0.94, 1.41) | 0.99 (0.80, 1.22) | 0.88 (0.71, 1.09) | 0.96 (0.78, 1.18) | 0.19 | 0.95 (0.87, 1.03) | 0.19 |
| Multivariable 1 | 1.23 (1.00, 1.51) | 1.06 (0.86, 1.32) | 0.95 (0.76, 1.19) | 1.01 (0.81, 1.26) | 0.42 | 0.97 (0.89, 1.05) | 0.43 |
| Multivariable 2 | 1.25 (1.02, 1.53) | 1.09 (0.88, 1.35) | 0.98 (0.79, 1.22) | 1.05 (0.84, 1.31) | 0.64 | 0.98 (0.90, 1.07) | 0.68 |
| **Alanine** |  |  |  |  |  |  |  |
| Age-adjusted | 0.94 (0.77, 1.16) | 0.89 (0.72, 1.10) | 1.03 (0.84, 1.26) | 0.86 (0.70, 1.06) | 0.28 | 0.96 (0.89, 1.05) | 0.38 |
| Multivariable 1 | 0.99 (0.80, 1.21) | 0.95 (0.77, 1.17) | 1.09 (0.89, 1.33) | 0.89 (0.71, 1.10) | 0.43 | 0.98 (0.90, 1.06) | 0.57 |
| Multivariable 2 | 1.00 (0.81, 1.23) | 0.96 (0.78, 1.19) | 1.11 (0.91, 1.37) | 0.92 (0.73, 1.14) | 0.63 | 0.99 (0.91, 1.08) | 0.82 |
| **Arginine** |  |  |  |  |  |  |  |
| Age-adjusted | 0.93 (0.76, 1.15) | 1.00 (0.82, 1.23) | 0.89 (0.72, 1.10) | 0.89 (0.72, 1.09) | 0.21 | 0.96 (0.89, 1.05) | 0.39 |
| Multivariable 1 | 0.98 (0.80, 1.21) | 1.06 (0.87, 1.30) | 0.95 (0.77, 1.18) | 0.92 (0.74, 1.14) | 0.37 | 0.98 (0.90, 1.06) | 0.59 |
| Multivariable 2 | 1.00 (0.81, 1.23) | 1.09 (0.89, 1.34) | 0.98 (0.79, 1.22) | 0.96 (0.77, 1.20) | 0.61 | 1.00 (0.91, 1.09) | 0.93 |
| **Asparagine** |  |  |  |  |  |  |  |
| Age-adjusted | 0.95 (0.77, 1.16) | 0.91 (0.74, 1.12) | 0.81 (0.66, 1.01) | 0.82 (0.67, 1.01) | 0.03 | 0.92 (0.85, 1.00) | 0.05 |
| Multivariable 1 | 1.00 (0.82, 1.23) | 0.97 (0.79, 1.20) | 0.88 (0.71, 1.09) | 0.87 (0.70, 1.07) | 0.10 | 0.94 (0.87, 1.02) | 0.17 |
| Multivariable 2 | 1.02 (0.83, 1.25) | 1.00 (0.81, 1.23) | 0.91 (0.73, 1.13) | 0.90 (0.72, 1.13) | 0.22 | 0.96 (0.88, 1.05) | 0.38 |
| **Aspartic acid** |  |  |  |  |  |  |  |
| Age-adjusted | 0.97 (0.79, 1.18) | 0.87 (0.70, 1.07) | 0.91 (0.74, 1.12) | 0.92 (0.75, 1.13) | 0.33 | 0.97 (0.89, 1.05) | 0.47 |
| Multivariable 1 | 1.01 (0.82, 1.23) | 0.91 (0.74, 1.12) | 0.96 (0.78, 1.19) | 0.94 (0.76, 1.16) | 0.43 | 0.97 (0.90, 1.06) | 0.55 |
| Multivariable 2 | 1.02 (0.83, 1.26) | 0.93 (0.75, 1.15) | 1.00 (0.80, 1.23) | 0.98 (0.79, 1.22) | 0.68 | 0.99 (0.91, 1.08) | 0.85 |
| **Cystine** |  |  |  |  |  |  |  |
| Age-adjusted | 0.89 (0.73, 1.09) | 0.86 (0.70, 1.05) | 0.84 (0.69, 1.04) | 0.79 (0.64, 0.98) | 0.03 | 0.91 (0.85, 0.99) | 0.02 |
| Multivariable 1 | 0.94 (0.77, 1.16) | 0.92 (0.75, 1.14) | 0.92 (0.75, 1.13) | 0.85 (0.69, 1.06) | 0.15 | 0.95 (0.87, 1.02) | 0.16 |
| Multivariable 2 | 0.96 (0.78, 1.18) | 0.94 (0.76, 1.16) | 0.95 (0.76, 1.17) | 0.88 (0.71, 1.10) | 0.28 | 0.96 (0.88, 1.04) | 0.32 |
| **Glutamine** |  |  |  |  |  |  |  |
| Age-adjusted | 0.91 (0.75, 1.11) | 0.88 (0.72, 1.08) | 0.74 (0.60, 0.92) | 0.81 (0.66, 0.99) | 0.01 | 0.87 (0.80, 0.94) | 0.001 |
| Multivariable 1 | 0.97 (0.80, 1.19) | 0.96 (0.79, 1.18) | 0.81 (0.65, 1.01) | 0.88 (0.71, 1.10) | 0.11 | 0.91 (0.83, 0.98) | 0.02 |
| Multivariable 2 | 0.99 (0.80, 1.21) | 0.98 (0.80, 1.21) | 0.83 (0.67, 1.04) | 0.91 (0.73, 1.14) | 0.19 | 0.91 (0.84, 1.00) | 0.04 |
| **Glutamic acid** |  |  |  |  |  |  |  |
| Age-adjusted | 0.99 (0.81, 1.22) | 0.92 (0.74, 1.13) | 0.95 (0.78, 1.17) | 0.90 (0.73, 1.11) | 0.30 | 0.96 (0.88, 1.04) | 0.28 |
| Multivariable 1 | 1.04 (0.85, 1.28) | 0.97 (0.79, 1.20) | 1.01 (0.82, 1.25) | 0.93 (0.75, 1.15) | 0.48 | 0.97 (0.89, 1.05) | 0.42 |
| Multivariable 2 | 1.06 (0.86, 1.30) | 0.99 (0.80, 1.23) | 1.05 (0.85, 1.30) | 0.97 (0.78, 1.21) | 0.74 | 0.98 (0.90, 1.07) | 0.68 |
| **Glycine** |  |  |  |  |  |  |  |
| Age-adjusted | 0.84 (0.68, 1.04) | 0.88 (0.72, 1.08) | 0.96 (0.79, 1.18) | 0.85 (0.69, 1.05) | 0.28 | 0.98 (0.91, 1.07) | 0.69 |
| Multivariable 1 | 0.88 (0.71, 1.08) | 0.93 (0.75, 1.14) | 1.01 (0.82, 1.24) | 0.87 (0.70, 1.08) | 0.38 | 0.99 (0.91, 1.08) | 0.85 |
| Multivariable 2 | 0.89 (0.72, 1.09) | 0.95 (0.77, 1.17) | 1.03 (0.84, 1.27) | 0.90 (0.72, 1.12) | 0.55 | 1.01 (0.93, 1.09) | 0.88 |
| **Proline** |  |  |  |  |  |  |  |
| Age-adjusted | 0.98 (0.80, 1.20) | 0.83 (0.67, 1.02) | 0.77 (0.62, 0.95) | 0.93 (0.76, 1.14) | 0.16 | 0.94 (0.87, 1.01) | 0.09 |
| Multivariable 1 | 1.05 (0.85, 1.28) | 0.89 (0.72, 1.10) | 0.84 (0.67, 1.04) | 0.99 (0.81, 1.23) | 0.46 | 0.96 (0.89, 1.04) | 0.33 |
| Multivariable 2 | 1.06 (0.86, 1.30) | 0.91 (0.73, 1.12) | 0.85 (0.68, 1.06) | 1.02 (0.83, 1.26) | 0.62 | 0.97 (0.90, 1.05) | 0.48 |
| **Serine** |  |  |  |  |  |  |  |
| Age-adjusted | 1.08 (0.89, 1.32) | 0.85 (0.69, 1.05) | 0.88 (0.72, 1.09) | 0.90 (0.73, 1.11) | 0.10 | 0.94 (0.86, 1.01) | 0.10 |
| Multivariable 1 | 1.15 (0.94, 1.41) | 0.91 (0.74, 1.13) | 0.96 (0.78, 1.19) | 0.95 (0.77, 1.18) | 0.31 | 0.96 (0.89, 1.04) | 0.32 |
| Multivariable 2 | 1.17 (0.96, 1.43) | 0.93 (0.75, 1.16) | 0.99 (0.79, 1.23) | 0.99 (0.79, 1.24) | 0.49 | 0.97 (0.90, 1.06) | 0.55 |
| **Tyrosine** |  |  |  |  |  |  |  |
| Age-adjusted | 1.02 (0.83, 1.25) | 0.97 (0.79, 1.19) | 0.93 (0.75, 1.14) | 0.87 (0.71, 1.08) | 0.15 | 0.95 (0.88, 1.03) | 0.19 |
| Multivariable 1 | 1.08 (0.88, 1.33) | 1.03 (0.84, 1.27) | 1.00 (0.81, 1.23) | 0.92 (0.74, 1.14) | 0.34 | 0.97 (0.89, 1.05) | 0.41 |
| Multivariable 2 | 1.09 (0.89, 1.34) | 1.05 (0.85, 1.30) | 1.02 (0.82, 1.27) | 0.95 (0.76, 1.18) | 0.51 | 0.98 (0.90, 1.06) | 0.63 |
| **Hydroxyproline** |  |  |  |  |  |  |  |
| Age-adjusted | 1.04 (0.85, 1.28) | 1.07 (0.87, 1.32) | 0.98 (0.79, 1.21) | 1.14 (0.93, 1.40) | 0.42 | 1.05 (0.96, 1.13) | 0.28 |
| Multivariable 1 | 1.05 (0.85, 1.29) | 1.07 (0.87, 1.32) | 0.96 (0.77, 1.20) | 1.07 (0.86, 1.32) | 0.88 | 1.01 (0.93, 1.10) | 0.75 |
| Multivariable 2 | 1.04 (0.85, 1.29) | 1.06 (0.86, 1.31) | 0.95 (0.76, 1.18) | 1.05 (0.84, 1.30) | 0.97 | 1.00 (0.92, 1.09) | 0.94 |
| **Taurine** |  |  |  |  |  |  |  |
| Age-adjusted | 1.04 (0.85, 1.28) | 0.95 (0.77, 1.18) | 0.90 (0.73, 1.12) | 0.99 (0.81, 1.22) | 0.61 | 0.97 (0.89, 1.05) | 0.43 |
| Multivariable 1 | 1.07 (0.87, 1.32) | 1.00 (0.81, 1.24) | 0.95 (0.77, 1.18) | 1.03 (0.83, 1.27) | 0.87 | 0.98 (0.90, 1.06) | 0.61 |
| Multivariable 2 | 1.09 (0.89, 1.34) | 1.03 (0.83, 1.28) | 0.99 (0.79, 1.23) | 1.08 (0.87, 1.35) | 0.78 | 1.00 (0.92, 1.09) | 0.96 |

1. The age-adjusted model was stratified by age (in years) and follow-up intervals. The multivariable model 1 was additionally adjusted for ethnicity (white, non-white), marital status (married, widowed, divorced/separated, unknown), smoking status (never, past, current: 1–14, 15–24, ≥25 cigarettes/day, unknown status or cigarette number), alcohol intake (never drinkers, 0.1-4.9, 5.0-9.9, 10.0-14.9, 15.0+ g/d), physical activity (<3, 3–9, 9–18, 18–27, 27–42, ≥42 metabolic equivalents/week, unknown), menopausal status and postmenopausal hormone use (premenopausal, postmenopausal: never, past, current users, NHS and NHSII only), oral contraception use (never, past, current user, NHSII only), multivitamin use (no, yes), aspirin use (no, yes), body mass index (<23, 23–24.9, 25–29.9, 30–34.9, ≥35 kg/m2, missing), baseline history of hypertension (no, yes), baseline hypercholesteremia (no, yes), baseline diabetes (no, yes), and energy intake (fifths of intake), and was considered the main model in these analyses. Multivariable model 2 additionally adjusted for the Alternative Healthy Eating Index (fifths of score). Results from NHS, NHSII and HPFS were pooled using a fixed-effects meta-analysis.
2. Linear trends estimated by fitting the median values in each fifth of intake as a pseudo-continuous variable in the Cox regression.
3. P-values estimated by fitting the continuous intakes of dietary amino acids in the Cox regression.

Supplementary table 10: Hazard ratios (95% confidence intervals) for total stroke (5997 cases) by fifths and per SD difference of energy-adjusted grams of dietary amino acids, across different adjustment models.

|  | **HR (95% CI), fifths of intake compared to bottom fifth^1^** | | | |  | **HR (95% CI)**  **per SD higher intake^1^** | ***p*-value^3^** |
| --- | --- | --- | --- | --- | --- | --- | --- |
| **Amino acids** | **Q2** | **Q3** | **Q4** | **Q5** | ***p*-trend^2^** |  |  |
| **Isoleucine** |  |  |  |  |  |  |  |
| Age-adjusted | 0.95 (0.88, 1.03) | 0.88 (0.82, 0.96) | 0.91 (0.84, 0.99) | 1.02 (0.94, 1.10) | 0.82 | 1.00 (0.97, 1.03) | 0.96 |
| Multivariable 1 | 0.98 (0.90, 1.06) | 0.91 (0.84, 0.99) | 0.93 (0.86, 1.01) | 0.98 (0.91, 1.07) | 0.53 | 0.98 (0.95, 1.01) | 0.26 |
| Multivariable 2 | 0.99 (0.91, 1.07) | 0.92 (0.85, 1.00) | 0.95 (0.88, 1.03) | 1.01 (0.93, 1.10) | 0.94 | 0.99 (0.96, 1.03) | 0.68 |
| **Leucine** |  |  |  |  |  |  |  |
| Age-adjusted | 0.93 (0.86, 1.01) | 0.89 (0.82, 0.96) | 0.92 (0.85, 1.00) | 1.01 (0.94, 1.09) | 0.75 | 1.00 (0.97, 1.03) | 0.89 |
| Multivariable 1 | 0.96 (0.89, 1.04) | 0.92 (0.85, 1.00) | 0.94 (0.87, 1.02) | 0.98 (0.90, 1.06) | 0.60 | 0.98 (0.95, 1.01) | 0.21 |
| Multivariable 2 | 0.97 (0.89, 1.05) | 0.93 (0.86, 1.01) | 0.96 (0.89, 1.04) | 1.00 (0.93, 1.09) | 0.90 | 0.99 (0.96, 1.02) | 0.54 |
| **Valine** |  |  |  |  |  |  |  |
| Age-adjusted | 0.94 (0.87, 1.02) | 0.89 (0.82, 0.96) | 0.91 (0.84, 0.99) | 0.99 (0.91, 1.07) | 0.56 | 0.99 (0.96, 1.02) | 0.46 |
| Multivariable 1 | 0.98 (0.90, 1.06) | 0.92 (0.85, 1.00) | 0.94 (0.87, 1.02) | 0.96 (0.89, 1.04) | 0.27 | 0.98 (0.95, 1.01) | 0.13 |
| Multivariable 2 | 0.99 (0.91, 1.07) | 0.93 (0.86, 1.01) | 0.96 (0.88, 1.04) | 0.99 (0.91, 1.08) | 0.71 | 0.99 (0.96, 1.02) | 0.44 |
| **Histidine** |  |  |  |  |  |  |  |
| Age-adjusted | 0.90 (0.83, 0.98) | 0.86 (0.80, 0.94) | 0.96 (0.89, 1.04) | 1.05 (0.97, 1.14) | 0.07 | 1.02 (0.99, 1.05) | 0.15 |
| Multivariable 1 | 0.93 (0.86, 1.01) | 0.89 (0.82, 0.96) | 0.97 (0.90, 1.05) | 0.99 (0.92, 1.07) | 0.75 | 0.99 (0.96, 1.02) | 0.63 |
| Multivariable 2 | 0.93 (0.86, 1.01) | 0.90 (0.83, 0.97) | 0.99 (0.91, 1.07) | 1.02 (0.94, 1.10) | 0.38 | 1.00 (0.97, 1.03) | 0.95 |
| **Lysine** |  |  |  |  |  |  |  |
| Age-adjusted | 0.96 (0.88, 1.03) | 0.91 (0.84, 0.98) | 0.95 (0.88, 1.03) | 1.07 (0.99, 1.16) | 0.08 | 1.02 (0.99, 1.05) | 0.16 |
| Multivariable 1 | 0.98 (0.90, 1.06) | 0.92 (0.85, 1.00) | 0.96 (0.88, 1.04) | 1.01 (0.94, 1.10) | 0.75 | 0.99 (0.96, 1.02) | 0.64 |
| Multivariable 2 | 0.98 (0.91, 1.07) | 0.93 (0.86, 1.01) | 0.97 (0.90, 1.06) | 1.04 (0.96, 1.13) | 0.36 | 1.00 (0.97, 1.03) | 0.90 |
| **Methionine** |  |  |  |  |  |  |  |
| Age-adjusted | 0.94 (0.87, 1.02) | 0.89 (0.82, 0.96) | 0.93 (0.86, 1.01) | 1.05 (0.97, 1.13) | 0.25 | 1.01 (0.98, 1.05) | 0.39 |
| Multivariable 1 | 0.97 (0.89, 1.05) | 0.91 (0.84, 0.99) | 0.94 (0.87, 1.02) | 1.00 (0.92, 1.08) | 0.89 | 0.99 (0.96, 1.02) | 0.42 |
| Multivariable 2 | 0.97 (0.90, 1.05) | 0.92 (0.85, 1.00) | 0.96 (0.89, 1.04) | 1.02 (0.94, 1.11) | 0.61 | 1.00 (0.97, 1.03) | 0.86 |
| **Phenylalanine** |  |  |  |  |  |  |  |
| Age-adjusted | 0.89 (0.82, 0.96) | 0.87 (0.81, 0.95) | 0.87 (0.80, 0.94) | 0.98 (0.91, 1.06) | 0.50 | 0.99 (0.96, 1.02) | 0.35 |
| Multivariable 1 | 0.92 (0.85, 1.00) | 0.91 (0.84, 0.98) | 0.90 (0.83, 0.97) | 0.96 (0.88, 1.04) | 0.24 | 0.97 (0.95, 1.01) | 0.11 |
| Multivariable 2 | 0.93 (0.86, 1.01) | 0.92 (0.85, 1.00) | 0.92 (0.85, 1.00) | 0.99 (0.91, 1.07) | 0.71 | 0.99 (0.96, 1.02) | 0.41 |
| **Threonine** |  |  |  |  |  |  |  |
| Age-adjusted | 0.96 (0.88, 1.03) | 0.89 (0.82, 0.96) | 0.95 (0.88, 1.03) | 1.06 (0.98, 1.14) | 0.16 | 1.02 (0.99, 1.05) | 0.27 |
| Multivariable 1 | 0.98 (0.91, 1.06) | 0.91 (0.84, 0.99) | 0.97 (0.89, 1.05) | 1.01 (0.93, 1.09) | 0.90 | 0.99 (0.96, 1.02) | 0.56 |
| Multivariable 2 | 0.99 (0.91, 1.07) | 0.92 (0.85, 1.00) | 0.99 (0.91, 1.07) | 1.03 (0.95, 1.12) | 0.43 | 1.00 (0.97, 1.03) | 0.94 |
| **Tryptophan** |  |  |  |  |  |  |  |
| Age-adjusted | 0.97 (0.89, 1.05) | 0.88 (0.82, 0.96) | 0.93 (0.86, 1.01) | 1.02 (0.95, 1.11) | 0.73 | 1.00 (0.97, 1.04) | 0.78 |
| Multivariable 1 | 1.01 (0.93, 1.09) | 0.92 (0.84, 1.00) | 0.96 (0.89, 1.05) | 1.00 (0.92, 1.08) | 0.70 | 0.99 (0.96, 1.02) | 0.40 |
| Multivariable 2 | 1.02 (0.94, 1.10) | 0.93 (0.86, 1.01) | 0.99 (0.91, 1.07) | 1.03 (0.94, 1.11) | 0.73 | 1.00 (0.97, 1.03) | 0.90 |
| **Alanine** |  |  |  |  |  |  |  |
| Age-adjusted | 0.89 (0.82, 0.97) | 0.88 (0.81, 0.95) | 0.97 (0.90, 1.05) | 1.05 (0.97, 1.13) | 0.06 | 1.03 (1.00, 1.06) | 0.05 |
| Multivariable 1 | 0.92 (0.84, 0.99) | 0.90 (0.83, 0.98) | 0.98 (0.91, 1.06) | 0.99 (0.91, 1.07) | 0.66 | 1.00 (0.97, 1.03) | 0.97 |
| Multivariable 2 | 0.92 (0.85, 1.00) | 0.91 (0.84, 0.99) | 1.00 (0.92, 1.08) | 1.02 (0.94, 1.10) | 0.26 | 1.01 (0.98, 1.04) | 0.49 |
| **Arginine** |  |  |  |  |  |  |  |
| Age-adjusted | 0.90 (0.83, 0.97) | 0.90 (0.83, 0.98) | 0.94 (0.87, 1.02) | 1.03 (0.96, 1.12) | 0.21 | 1.03 (1.00, 1.06) | 0.10 |
| Multivariable 1 | 0.92 (0.85, 1.00) | 0.93 (0.85, 1.00) | 0.96 (0.88, 1.04) | 0.99 (0.91, 1.07) | 0.93 | 1.00 (0.97, 1.03) | 0.98 |
| Multivariable 2 | 0.93 (0.86, 1.01) | 0.94 (0.87, 1.02) | 0.98 (0.91, 1.07) | 1.02 (0.94, 1.11) | 0.33 | 1.02 (0.98, 1.05) | 0.37 |
| **Asparagine** |  |  |  |  |  |  |  |
| Age-adjusted | 0.89 (0.82, 0.96) | 0.88 (0.82, 0.96) | 0.86 (0.80, 0.93) | 0.96 (0.89, 1.04) | 0.25 | 0.99 (0.96, 1.02) | 0.40 |
| Multivariable 1 | 0.93 (0.85, 1.00) | 0.93 (0.85, 1.00) | 0.91 (0.83, 0.98) | 0.96 (0.89, 1.04) | 0.28 | 0.98 (0.95, 1.01) | 0.27 |
| Multivariable 2 | 0.94 (0.87, 1.02) | 0.95 (0.87, 1.03) | 0.93 (0.86, 1.01) | 1.00 (0.92, 1.09) | 1.00 | 1.00 (0.97, 1.03) | 0.97 |
| **Aspartic acid** |  |  |  |  |  |  |  |
| Age-adjusted | 0.92 (0.85, 1.00) | 0.87 (0.80, 0.94) | 0.94 (0.87, 1.01) | 1.11 (1.03, 1.20) | 0.004 | 1.05 (1.02, 1.08) | 0.003 |
| Multivariable 1 | 0.94 (0.87, 1.02) | 0.88 (0.81, 0.96) | 0.94 (0.86, 1.01) | 1.02 (0.95, 1.11) | 0.41 | 1.01 (0.97, 1.04) | 0.75 |
| Multivariable 2 | 0.95 (0.87, 1.03) | 0.90 (0.83, 0.97) | 0.96 (0.88, 1.04) | 1.06 (0.98, 1.15) | 0.09 | 1.02 (0.99, 1.05) | 0.25 |
| **Cystine** |  |  |  |  |  |  |  |
| Age-adjusted | 0.87 (0.80, 0.94) | 0.84 (0.78, 0.91) | 0.87 (0.80, 0.94) | 0.90 (0.84, 0.98) | 0.02 | 0.97 (0.94, 1.00) | 0.03 |
| Multivariable 1 | 0.90 (0.84, 0.98) | 0.89 (0.82, 0.96) | 0.91 (0.84, 0.99) | 0.92 (0.85, 0.99) | 0.08 | 0.97 (0.95, 1.00) | 0.08 |
| Multivariable 2 | 0.91 (0.84, 0.99) | 0.90 (0.83, 0.98) | 0.93 (0.86, 1.01) | 0.95 (0.87, 1.03) | 0.34 | 0.99 (0.96, 1.02) | 0.36 |
| **Glutamine** |  |  |  |  |  |  |  |
| Age-adjusted | 0.87 (0.81, 0.94) | 0.82 (0.75, 0.88) | 0.83 (0.77, 0.90) | 0.81 (0.75, 0.88) | <0.001 | 0.91 (0.88, 0.94) | <0.001 |
| Multivariable 1 | 0.92 (0.85, 1.00) | 0.88 (0.81, 0.95) | 0.90 (0.83, 0.98) | 0.87 (0.80, 0.94) | <0.001 | 0.94 (0.91, 0.97) | <0.001 |
| Multivariable 2 | 0.93 (0.86, 1.01) | 0.89 (0.82, 0.97) | 0.92 (0.85, 1.00) | 0.89 (0.81, 0.96) | 0.006 | 0.94 (0.91, 0.98) | <0.001 |
| **Glutamic acid** |  |  |  |  |  |  |  |
| Age-adjusted | 0.90 (0.84, 0.98) | 0.87 (0.80, 0.94) | 0.90 (0.83, 0.98) | 1.03 (0.95, 1.11) | 0.38 | 1.02 (0.98, 1.05) | 0.32 |
| Multivariable 1 | 0.93 (0.86, 1.00) | 0.89 (0.82, 0.97) | 0.92 (0.85, 0.99) | 0.98 (0.91, 1.06) | 0.71 | 0.99 (0.96, 1.02) | 0.46 |
| Multivariable 2 | 0.94 (0.86, 1.01) | 0.91 (0.84, 0.99) | 0.94 (0.86, 1.02) | 1.01 (0.93, 1.10) | 0.68 | 1.00 (0.97, 1.03) | 0.98 |
| **Glycine** |  |  |  |  |  |  |  |
| Age-adjusted | 0.87 (0.81, 0.95) | 0.90 (0.83, 0.98) | 1.00 (0.92, 1.08) | 1.07 (0.99, 1.16) | 0.01 | 1.04 (1.01, 1.08) | 0.01 |
| Multivariable 1 | 0.89 (0.82, 0.96) | 0.92 (0.85, 1.00) | 0.99 (0.92, 1.07) | 1.00 (0.92, 1.08) | 0.32 | 1.01 (0.98, 1.04) | 0.65 |
| Multivariable 2 | 0.90 (0.83, 0.97) | 0.93 (0.86, 1.01) | 1.01 (0.93, 1.10) | 1.03 (0.95, 1.11) | 0.10 | 1.02 (0.99, 1.05) | 0.28 |
| **Proline** |  |  |  |  |  |  |  |
| Age-adjusted | 0.93 (0.86, 1.01) | 0.85 (0.78, 0.92) | 0.88 (0.81, 0.95) | 0.90 (0.83, 0.97) | 0.003 | 0.96 (0.93, 0.98) | 0.003 |
| Multivariable 1 | 0.97 (0.90, 1.05) | 0.88 (0.81, 0.96) | 0.91 (0.84, 0.99) | 0.90 (0.83, 0.98) | 0.004 | 0.96 (0.93, 0.99) | 0.004 |
| Multivariable 2 | 0.98 (0.91, 1.06) | 0.89 (0.82, 0.97) | 0.92 (0.85, 1.00) | 0.92 (0.84, 1.00) | 0.02 | 0.96 (0.93, 0.99) | 0.02 |
| **Serine** |  |  |  |  |  |  |  |
| Age-adjusted | 0.90 (0.83, 0.97) | 0.87 (0.80, 0.94) | 0.88 (0.82, 0.96) | 0.95 (0.88, 1.03) | 0.23 | 0.98 (0.95, 1.01) | 0.21 |
| Multivariable 1 | 0.93 (0.86, 1.01) | 0.91 (0.84, 0.99) | 0.91 (0.84, 0.99) | 0.94 (0.87, 1.02) | 0.13 | 0.97 (0.94, 1.00) | 0.08 |
| Multivariable 2 | 0.94 (0.87, 1.02) | 0.93 (0.85, 1.00) | 0.93 (0.86, 1.01) | 0.97 (0.89, 1.05) | 0.45 | 0.98 (0.95, 1.02) | 0.33 |
| **Tyrosine** |  |  |  |  |  |  |  |
| Age-adjusted | 0.93 (0.86, 1.01) | 0.91 (0.84, 0.98) | 0.92 (0.85, 1.00) | 0.99 (0.91, 1.07) | 0.75 | 0.99 (0.96, 1.02) | 0.62 |
| Multivariable 1 | 0.96 (0.88, 1.04) | 0.93 (0.86, 1.01) | 0.94 (0.87, 1.02) | 0.95 (0.88, 1.03) | 0.24 | 0.98 (0.95, 1.01) | 0.11 |
| Multivariable 2 | 0.96 (0.89, 1.04) | 0.95 (0.87, 1.02) | 0.96 (0.88, 1.04) | 0.98 (0.90, 1.06) | 0.59 | 0.98 (0.95, 1.02) | 0.34 |
| **Hydroxyproline** |  |  |  |  |  |  |  |
| Age-adjusted | 1.02 (0.94, 1.11) | 1.11 (1.02, 1.20) | 1.12 (1.03, 1.21) | 1.39 (1.29, 1.50) | <0.001 | 1.15 (1.11, 1.18) | <0.001 |
| Multivariable 1 | 0.99 (0.91, 1.07) | 1.05 (0.97, 1.14) | 1.02 (0.94, 1.11) | 1.15 (1.06, 1.24) | <0.001 | 1.05 (1.02, 1.08) | 0.002 |
| Multivariable 2 | 0.98 (0.91, 1.07) | 1.04 (0.96, 1.13) | 1.01 (0.93, 1.10) | 1.13 (1.04, 1.23) | 0.001 | 1.04 (1.01, 1.08) | 0.01 |
| **Taurine** |  |  |  |  |  |  |  |
| Age-adjusted | 0.95 (0.87, 1.03) | 0.90 (0.83, 0.97) | 0.94 (0.87, 1.02) | 1.04 (0.96, 1.12) | 0.28 | 1.01 (0.98, 1.05) | 0.38 |
| Multivariable 1 | 0.97 (0.89, 1.05) | 0.92 (0.85, 1.00) | 0.96 (0.88, 1.04) | 1.02 (0.95, 1.11) | 0.47 | 1.00 (0.97, 1.03) | 0.98 |
| Multivariable 2 | 0.98 (0.90, 1.06) | 0.94 (0.86, 1.02) | 0.99 (0.91, 1.07) | 1.07 (0.98, 1.16) | 0.09 | - 1. (0.98, 1.05) | 0.37 |

1. The age-adjusted model was stratified by age (in years) and follow-up intervals. The multivariable model 1 was additionally adjusted for ethnicity (white, non-white), marital status (married, widowed, divorced/separated, unknown), smoking status (never, past, current: 1–14, 15–24, ≥25 cigarettes/day, unknown status or cigarette number), alcohol intake (never drinkers, 0.1-4.9, 5.0-9.9, 10.0-14.9, 15.0+ g/d), physical activity (<3, 3–9, 9–18, 18–27, 27–42, ≥42 metabolic equivalents/week, unknown), menopausal status and postmenopausal hormone use (premenopausal, postmenopausal: never, past, current users, NHS and NHSII only), oral contraception use (never, past, current user, NHSII only), multivitamin use (no, yes), aspirin use (no, yes), body mass index (<23, 23–24.9, 25–29.9, 30–34.9, ≥35 kg/m2, missing), baseline history of hypertension (no, yes), baseline hypercholesteremia (no, yes), baseline diabetes (no, yes), and energy intake (fifths of intake), and was considered the main model in these analyses. Multivariable model 2 additionally adjusted for the Alternative Healthy Eating Index (fifths of score). Results from NHS, NHSII and HPFS were pooled using a fixed-effects meta-analysis.
2. Linear trends estimated by fitting the median values in each fifth of intake as a pseudo-continuous variable in the Cox regression.
3. P-values estimated by fitting the continuous intakes of dietary amino acids in the Cox regression.

Supplementary table 11: Hazard ratios (95% confidence intervals) for ischemic stroke (3058 cases) by fifths and per SD difference of raw grams of dietary amino acids.

|  | **HR (95% CI), fifths of intake compared to bottom fifth^1^** | | | |  | **HR (95% CI)**  **per SD higher intake^1^** | ***p*-value^3^** |
| --- | --- | --- | --- | --- | --- | --- | --- |
| **Amino acids** | **Q2** | **Q3** | **Q4** | **Q5** | ***p*-trend^2^** |  |  |
| Isoleucine | 1.00 (0.89, 1.13) | 1.03 (0.90, 1.18) | 0.94 (0.81, 1.09) | 0.87 (0.73, 1.03) | 0.05 | 0.94 (0.88, 1.01) | 0.07 |
| Leucine | 1.00 (0.88, 1.13) | 1.02 (0.89, 1.17) | 0.92 (0.79, 1.07) | 0.91 (0.76, 1.08) | 0.16 | 0.94 (0.88, 1.00) | 0.06 |
| Valine | 1.02 (0.90, 1.15) | 1.01 (0.88, 1.16) | 0.91 (0.78, 1.06) | 0.87 (0.73, 1.04) | 0.05 | 0.93 (0.87, 1.00) | 0.04 |
| Histidine | 0.95 (0.84, 1.07) | 1.00 (0.88, 1.15) | 0.93 (0.80, 1.08) | 0.90 (0.76, 1.07) | 0.19 | 0.95 (0.89, 1.02) | 0.14 |
| Lysine | 0.95 (0.84, 1.07) | 1.00 (0.88, 1.14) | 0.89 (0.77, 1.03) | 0.92 (0.78, 1.08) | 0.24 | 0.95 (0.90, 1.01) | 0.12 |
| Methionine | 0.96 (0.85, 1.09) | 1.04 (0.91, 1.19) | 0.94 (0.81, 1.09) | 0.91 (0.77, 1.07) | 0.22 | 0.95 (0.89, 1.01) | 0.08 |
| Phenylalanine | 1.00 (0.89, 1.14) | 1.00 (0.87, 1.15) | 0.89 (0.76, 1.05) | 0.87 (0.72, 1.04) | 0.07 | 0.93 (0.87, 1.00) | 0.04 |
| Threonine | 0.95 (0.84, 1.08) | 1.01 (0.88, 1.16) | 0.92 (0.79, 1.07) | 0.90 (0.76, 1.07) | 0.17 | 0.95 (0.89, 1.01) | 0.13 |
| Tryptophan | 0.99 (0.87, 1.12) | 1.05 (0.91, 1.20) | 0.94 (0.80, 1.09) | 0.94 (0.78, 1.12) | 0.29 | 0.94 (0.88, 1.01) | 0.09 |
| Alanine | 0.98 (0.87, 1.11) | 1.02 (0.89, 1.17) | 0.95 (0.82, 1.10) | 0.97 (0.82, 1.14) | 0.57 | 0.96 (0.90, 1.02) | 0.21 |
| Arginine | 1.03 (0.91, 1.16) | 1.09 (0.95, 1.25) | 0.98 (0.84, 1.14) | 1.00 (0.84, 1.18) | 0.69 | 0.96 (0.89, 1.02) | 0.17 |
| Asparagine | 0.98 (0.86, 1.11) | 1.03 (0.90, 1.19) | 0.95 (0.82, 1.11) | 0.91 (0.76, 1.08) | 0.20 | 0.95 (0.89, 1.01) | 0.11 |
| Aspartic acid | 0.93 (0.82, 1.05) | 1.03 (0.91, 1.18) | 0.93 (0.81, 1.08) | 0.96 (0.82, 1.13) | 0.65 | 0.97 (0.91, 1.03) | 0.31 |
| Cystine | 0.96 (0.85, 1.09) | 1.01 (0.88, 1.17) | 0.95 (0.81, 1.11) | 0.85 (0.71, 1.02) | 0.07 | 0.95 (0.89, 1.01) | 0.12 |
| Glutamine | 1.12 (0.98, 1.27) | 1.00 (0.86, 1.16) | 0.95 (0.80, 1.12) | 0.82 (0.68, 1.00) | 0.02 | 0.91 (0.84, 0.98) | 0.014 |
| Glutamic acid | 0.97 (0.86, 1.09) | 0.98 (0.86, 1.12) | 0.95 (0.82, 1.09) | 0.90 (0.77, 1.06) | 0.17 | 0.94 (0.89, 1.00) | 0.07 |
| Glycine | 0.98 (0.87, 1.11) | 0.97 (0.85, 1.11) | 1.00 (0.86, 1.15) | 0.97 (0.82, 1.15) | 0.72 | 0.97 (0.91, 1.03) | 0.31 |
| Proline | 1.00 (0.88, 1.13) | 0.95 (0.83, 1.10) | 0.95 (0.81, 1.11) | 0.79 (0.66, 0.95) | 0.009 | 0.92 (0.85, 0.98) | 0.02 |
| Serine | 1.03 (0.90, 1.16) | 1.00 (0.86, 1.15) | 0.96 (0.82, 1.13) | 0.87 (0.73, 1.05) | 0.09 | 0.93 (0.87, 1.00) | 0.04 |
| Tyrosine | 1.00 (0.89, 1.13) | 1.00 (0.87, 1.15) | 0.92 (0.78, 1.07) | 0.85 (0.71, 1.01) | 0.03 | 0.92 (0.86, 0.99) | 0.02 |
| Hydroxyproline | 0.90 (0.80, 1.01) | 0.94 (0.84, 1.06) | 1.03 (0.91, 1.17) | 1.07 (0.94, 1.22) | 0.08 | 1.02 (0.97, 1.08) | 0.44 |
| Taurine | 0.96 (0.86, 1.08) | 0.88 (0.78, 1.00) | 0.99 (0.88, 1.11) | 0.90 (0.79, 1.02) | 0.13 | 0.97 (0.93, 1.02) | 0.25 |

1. Analyses were based on non-energy adjusted grams of intake of dietary amino acids. The model was stratified by age (in years) and follow-up intervals, and adjusted for ethnicity (white, non-white), marital status (married, widowed, divorced/separated, unknown), smoking status (never, past, current: 1–14, 15–24, ≥25 cigarettes/day, unknown status or cigarette number), alcohol intake (never drinkers, 0.1-4.9, 5.0-9.9, 10.0-14.9, 15.0+ g/d), physical activity (<3, 3–9, 9–18, 18–27, 27–42, ≥42 metabolic equivalents/week, unknown), menopausal status and postmenopausal hormone use (premenopausal, postmenopausal: never, past, current users, NHS and NHSII only), oral contraception use (never, past, current user, NHSII only), multivitamin use (no, yes), aspirin use (no, yes), body mass index (<23, 23–24.9, 25–29.9, 30–34.9, ≥35 kg/m2, missing), baseline history of hypertension (no, yes), baseline hypercholesteremia (no, yes), baseline diabetes (no, yes), and energy intake (fifths of intake). Results from NHS, NHSII and HPFS were pooled using a fixed-effects meta-analysis.
2. Linear trends estimated by fitting the median values in each fifth of intake as a pseudo-continuous variable in the Cox regression.
3. P-values estimated by fitting the continuous intakes of dietary amino acids in the Cox regression.

Supplementary table 12: Hazard ratios (95% confidence intervals) for hemorrhagic stroke (872 cases) by fifths and per SD difference of raw grams of dietary amino acids.

|  | **HR (95% CI), fifths of intake compared to bottom fifth^1^** | | | |  | **HR (95% CI)**  **per SD higher intake^1^** | ***p*-value^3^** |
| --- | --- | --- | --- | --- | --- | --- | --- |
| **Amino acids** | **Q2** | **Q3** | **Q4** | **Q5** | ***p*-trend^2^** |  |  |
| Isoleucine | 0.92 (0.74, 1.15) | 0.82 (0.64, 1.05) | 0.80 (0.61, 1.06) | 0.77 (0.56, 1.06) | 0.15 | 0.95 (0.84, 1.07) | 0.37 |
| Leucine | 0.85 (0.68, 1.06) | 0.80 (0.63, 1.03) | 0.74 (0.56, 0.98) | 0.74 (0.53, 1.02) | 0.10 | 0.95 (0.83, 1.08) | 0.40 |
| Valine | 0.87 (0.69, 1.08) | 0.80 (0.62, 1.03) | 0.77 (0.58, 1.02) | 0.75 (0.54, 1.04) | 0.14 | 0.94 (0.83, 1.07) | 0.36 |
| Histidine | 0.91 (0.73, 1.13) | 0.77 (0.60, 1.00) | 0.82 (0.62, 1.08) | 0.75 (0.54, 1.03) | 0.11 | 0.96 (0.84, 1.08) | 0.48 |
| Lysine | 0.93 (0.75, 1.15) | 0.79 (0.62, 1.00) | 0.89 (0.69, 1.15) | 0.81 (0.60, 1.10) | 0.28 | 0.97 (0.86, 1.08) | 0.55 |
| Methionine | 0.93 (0.75, 1.15) | 0.76 (0.60, 0.98) | 0.84 (0.64, 1.09) | 0.76 (0.56, 1.03) | 0.12 | 0.95 (0.84, 1.07) | 0.42 |
| Phenylalanine | 0.84 (0.67, 1.05) | 0.76 (0.59, 0.99) | 0.72 (0.54, 0.97) | 0.70 (0.50, 0.98) | 0.07 | 0.94 (0.82, 1.07) | 0.36 |
| Threonine | 0.87 (0.70, 1.08) | 0.79 (0.61, 1.01) | 0.83 (0.63, 1.09) | 0.80 (0.58, 1.09) | 0.28 | 0.95 (0.84, 1.07) | 0.41 |
| Tryptophan | 0.90 (0.72, 1.13) | 0.88 (0.68, 1.14) | 0.72 (0.54, 0.96) | 0.81 (0.59, 1.13) | 0.23 | 0.95 (0.84, 1.08) | 0.45 |
| Alanine | 0.90 (0.73, 1.12) | 0.76 (0.59, 0.98) | 0.85 (0.65, 1.11) | 0.80 (0.59, 1.09) | 0.26 | 0.97 (0.86, 1.09) | 0.58 |
| Arginine | 0.91 (0.73, 1.14) | 0.87 (0.68, 1.11) | 0.85 (0.65, 1.13) | 0.78 (0.57, 1.08) | 0.19 | 0.97 (0.86, 1.10) | 0.62 |
| Asparagine | 0.95 (0.76, 1.19) | 0.78 (0.61, 1.01) | 0.79 (0.59, 1.04) | 0.73 (0.53, 1.01) | 0.07 | 0.92 (0.81, 1.04) | 0.19 |
| Aspartic acid | 0.98 (0.79, 1.21) | 0.80 (0.63, 1.02) | 0.90 (0.70, 1.17) | 0.87 (0.65, 1.17) | 0.46 | 0.97 (0.87, 1.09) | 0.62 |
| Cystine | 0.85 (0.68, 1.07) | 0.75 (0.58, 0.98) | 0.71 (0.53, 0.95) | 0.62 (0.44, 0.87) | 0.01 | 0.91 (0.80, 1.04) | 0.18 |
| Glutamine | 0.92 (0.73, 1.16) | 0.87 (0.66, 1.14) | 0.68 (0.50, 0.93) | 0.68 (0.47, 0.98) | 0.02 | 0.84 (0.73, 0.97) | 0.02 |
| Glutamic acid | 0.97 (0.78, 1.21) | 0.81 (0.64, 1.04) | 0.89 (0.68, 1.16) | 0.89 (0.66, 1.21) | 0.56 | 0.96 (0.85, 1.07) | 0.45 |
| Glycine | 0.89 (0.71, 1.10) | 0.82 (0.64, 1.05) | 0.90 (0.69, 1.17) | 0.81 (0.60, 1.11) | 0.32 | 0.99 (0.88, 1.11) | 0.83 |
| Proline | 0.89 (0.71, 1.12) | 0.85 (0.65, 1.11) | 0.80 (0.59, 1.08) | 0.79 (0.56, 1.11) | 0.34 | 0.93 (0.81, 1.06) | 0.25 |
| Serine | 0.92 (0.73, 1.15) | 0.79 (0.61, 1.03) | 0.75 (0.56, 1.02) | 0.73 (0.52, 1.04) | 0.10 | 0.93 (0.82, 1.07) | 0.32 |
| Tyrosine | 0.88 (0.71, 1.11) | 0.92 (0.72, 1.19) | 0.80 (0.60, 1.07) | 0.83 (0.60, 1.15) | 0.36 | 0.95 (0.83, 1.08) | 0.41 |
| Hydroxyproline | 1.10 (0.89, 1.35) | 1.08 (0.87, 1.35) | 1.06 (0.84, 1.34) | 1.06 (0.82, 1.37) | 0.68 | 1.00 (0.91, 1.11) | 0.93 |
| Taurine | 0.98 (0.80, 1.21) | 0.85 (0.68, 1.06) | 0.90 (0.72, 1.12) | 0.95 (0.76, 1.20) | 0.68 | 0.98 (0.90, 1.08) | 0.71 |

1. Analyses were based on non-energy adjusted grams of intake of dietary amino acids. The model was stratified by age (in years) and follow-up intervals, and adjusted for ethnicity (white, non-white), marital status (married, widowed, divorced/separated, unknown), smoking status (never, past, current: 1–14, 15–24, ≥25 cigarettes/day, unknown status or cigarette number), alcohol intake (never drinkers, 0.1-4.9, 5.0-9.9, 10.0-14.9, 15.0+ g/d), physical activity (<3, 3–9, 9–18, 18–27, 27–42, ≥42 metabolic equivalents/week, unknown), menopausal status and postmenopausal hormone use (premenopausal, postmenopausal: never, past, current users, NHS and NHSII only), oral contraception use (never, past, current user, NHSII only), multivitamin use (no, yes), aspirin use (no, yes), body mass index (<23, 23–24.9, 25–29.9, 30–34.9, ≥35 kg/m2, missing), baseline history of hypertension (no, yes), baseline hypercholesteremia (no, yes), baseline diabetes (no, yes), and energy intake (fifths of intake). Results from NHS, NHSII and HPFS were pooled using a fixed-effects meta-analysis.
2. Linear trends estimated by fitting the median values in each fifth of intake as a pseudo-continuous variable in the Cox regression.
3. P-values estimated by fitting the continuous intakes of dietary amino acids in the Cox regression.

Supplementary table 13: Hazard ratios (95% confidence intervals) for total stroke (5997 cases) by fifths and per SD difference of raw grams of dietary amino acids.

|  | **HR (95% CI), fifths of intake compared to bottom fifth^1^** | | | |  | **HR (95% CI)**  **per SD higher intake^1^** | ***p*-value^3^** |
| --- | --- | --- | --- | --- | --- | --- | --- |
| **Amino acids** | **Q2** | **Q3** | **Q4** | **Q5** | ***p*-trend^2^** |  |  |
| Isoleucine | 1.02 (0.94, 1.11) | 1.03 (0.94, 1.14) | 0.99 (0.89, 1.11) | 0.93 (0.82, 1.05) | 0.15 | 0.97 (0.93, 1.02) | 0.22 |
| Leucine | 0.99 (0.91, 1.08) | 1.03 (0.93, 1.14) | 0.96 (0.86, 1.07) | 0.93 (0.82, 1.06) | 0.21 | 0.97 (0.92, 1.02) | 0.18 |
| Valine | 1.02 (0.94, 1.11) | 1.03 (0.93, 1.13) | 0.97 (0.87, 1.08) | 0.93 (0.82, 1.05) | 0.15 | 0.96 (0.92, 1.01) | 0.12 |
| Histidine | 1.02 (0.93, 1.11) | 1.02 (0.92, 1.12) | 1.02 (0.91, 1.13) | 0.96 (0.85, 1.08) | 0.44 | 0.98 (0.94, 1.03) | 0.50 |
| Lysine | 1.02 (0.94, 1.11) | 1.02 (0.93, 1.12) | 0.99 (0.90, 1.10) | 0.99 (0.88, 1.11) | 0.72 | 0.99 (0.94, 1.03) | 0.55 |
| Methionine | 1.01 (0.93, 1.10) | 1.03 (0.94, 1.13) | 1.01 (0.91, 1.12) | 0.96 (0.85, 1.08) | 0.43 | 0.98 (0.94, 1.03) | 0.39 |
| Phenylalanine | 1.00 (0.91, 1.09) | 1.03 (0.93, 1.14) | 0.96 (0.85, 1.07) | 0.92 (0.81, 1.05) | 0.14 | 0.96 (0.91, 1.01) | 0.10 |
| Threonine | 1.00 (0.92, 1.09) | 1.03 (0.94, 1.14) | 1.01 (0.91, 1.12) | 0.97 (0.86, 1.09) | 0.54 | 0.98 (0.94, 1.03) | 0.45 |
| Tryptophan | 1.00 (0.92, 1.09) | 1.05 (0.95, 1.16) | 0.97 (0.87, 1.08) | 0.98 (0.86, 1.11) | 0.50 | 0.97 (0.93, 1.02) | 0.30 |
| Alanine | 1.02 (0.94, 1.11) | 1.04 (0.95, 1.15) | 1.02 (0.92, 1.13) | 1.01 (0.90, 1.13) | 0.98 | 1.00 (0.95, 1.04) | 0.83 |
| Arginine | 1.04 (0.95, 1.13) | 1.11 (1.01, 1.22) | 1.05 (0.94, 1.17) | 1.02 (0.90, 1.15) | 0.89 | 0.99 (0.95, 1.04) | 0.74 |
| Asparagine | 1.02 (0.93, 1.11) | 1.03 (0.94, 1.14) | 1.01 (0.90, 1.12) | 0.94 (0.83, 1.07) | 0.25 | 0.97 (0.93, 1.02) | 0.23 |
| Aspartic acid | 0.99 (0.91, 1.08) | 1.05 (0.96, 1.15) | 1.04 (0.94, 1.15) | 1.02 (0.91, 1.14) | 0.64 | 1.00 (0.96, 1.05) | 0.91 |
| Cystine | 0.96 (0.88, 1.05) | 1.00 (0.90, 1.10) | 0.97 (0.86, 1.08) | 0.85 (0.75, 0.97) | 0.02 | 0.96 (0.91, 1.01) | 0.09 |
| Glutamine | 1.00 (0.92, 1.10) | 0.95 (0.85, 1.06) | 0.88 (0.78, 0.99) | 0.77 (0.67, 0.88) | <0.001 | 0.90 (0.85, 0.95) | <0.001 |
| Glutamic acid | 1.00 (0.92, 1.09) | 1.01 (0.92, 1.11) | 1.02 (0.92, 1.13) | 0.97 (0.87, 1.09) | 0.59 | 0.98 (0.94, 1.02) | 0.38 |
| Glycine | 1.02 (0.93, 1.11) | 1.02 (0.93, 1.12) | 1.06 (0.96, 1.18) | 1.01 (0.90, 1.14) | 0.87 | 1.00 (0.96, 1.05) | 0.91 |
| Proline | 0.99 (0.91, 1.09) | 1.00 (0.90, 1.11) | 0.93 (0.83, 1.04) | 0.86 (0.75, 0.98) | 0.02 | 0.93 (0.89, 0.98) | 0.007 |
| Serine | 1.02 (0.94, 1.12) | 1.00 (0.90, 1.11) | 0.98 (0.88, 1.10) | 0.91 (0.79, 1.03) | 0.09 | 0.96 (0.91, 1.01) | 0.08 |
| Tyrosine | 1.00 (0.92, 1.09) | 1.04 (0.95, 1.15) | 0.96 (0.86, 1.07) | 0.91 (0.81, 1.04) | 0.10 | 0.96 (0.92, 1.01) | 0.10 |
| Hydroxyproline | 1.01 (0.93, 1.09) | 1.00 (0.92, 1.09) | 1.10 (1.01, 1.20) | 1.13 (1.03, 1.24) | 0.002 | 1.05 (1.01, 1.09) | 0.01 |
| Taurine | 0.98 (0.90, 1.06) | 0.96 (0.88, 1.05) | 1.03 (0.95, 1.12) | 0.97 (0.89, 1.06) | 0.77 | 1.00 (0.96, 1.03) | 0.89 |

1. Analyses were based on non-energy adjusted grams of intake of dietary amino acids. The model was stratified by age (in years) and follow-up intervals, and adjusted for ethnicity (white, non-white), marital status (married, widowed, divorced/separated, unknown), smoking status (never, past, current: 1–14, 15–24, ≥25 cigarettes/day, unknown status or cigarette number), alcohol intake (never drinkers, 0.1-4.9, 5.0-9.9, 10.0-14.9, 15.0+ g/d), physical activity (<3, 3–9, 9–18, 18–27, 27–42, ≥42 metabolic equivalents/week, unknown), menopausal status and postmenopausal hormone use (premenopausal, postmenopausal: never, past, current users, NHS and NHSII only), oral contraception use (never, past, current user, NHSII only), multivitamin use (no, yes), aspirin use (no, yes), body mass index (<23, 23–24.9, 25–29.9, 30–34.9, ≥35 kg/m2, missing), baseline history of hypertension (no, yes), baseline hypercholesteremia (no, yes), baseline diabetes (no, yes), and energy intake (fifths of intake). Results from NHS, NHSII and HPFS were pooled using a fixed-effects meta-analysis.
2. Linear trends estimated by fitting the median values in each fifth of intake as a pseudo-continuous variable in the Cox regression.
3. P-values estimated by fitting the continuous intakes of dietary amino acids in the Cox regression.

Supplementary table 14: Hazard ratios (95% confidence intervals) for ischemic, hemorrhagic and total stroke by per SD difference of energy-adjusted grams of dietary amino acids, with a 4-year lag.

|  | **Ischemic stroke (2832 cases)** | | **Hemorrhagic stroke (775 cases)** | | **Total stroke (5610 cases)** | |
| --- | --- | --- | --- | --- | --- | --- |
| **Amino acids** | **HR (95% CI)^1^** | ***p*-value^2^** | **HR (95% CI)^1^** | ***p*-value^2^** | **HR (95% CI)^1^** | ***p*-value^2^** |
| Isoleucine | 0.97 (0.93, 1.01) | 0.16 | 0.98 (0.90, 1.07) | 0.64 | 0.98 (0.95, 1.02) | 0.33 |
| Leucine | 0.97 (0.92, 1.01) | 0.13 | 0.99 (0.91, 1.07) | 0.76 | 0.98 (0.95, 1.01) | 0.29 |
| Valine | 0.96 (0.92, 1.01) | 0.09 | 0.98 (0.90, 1.07) | 0.66 | 0.98 (0.95, 1.01) | 0.20 |
| Histidine | 0.98 (0.94, 1.02) | 0.32 | 0.99 (0.91, 1.08) | 0.90 | 0.99 (0.96, 1.03) | 0.74 |
| Lysine | 0.97 (0.93, 1.02) | 0.21 | 0.99 (0.91, 1.08) | 0.86 | 0.99 (0.96, 1.02) | 0.67 |
| Methionine | 0.97 (0.93, 1.01) | 0.17 | 0.99 (0.91, 1.08) | 0.78 | 0.99 (0.96, 1.02) | 0.54 |
| Phenylalanine | 0.96 (0.92, 1.01) | 0.11 | 0.98 (0.90, 1.07) | 0.70 | 0.98 (0.95, 1.01) | 0.21 |
| Threonine | 0.97 (0.93, 1.02) | 0.24 | 0.99 (0.91, 1.07) | 0.77 | 0.99 (0.96, 1.02) | 0.62 |
| Tryptophan | 0.97 (0.93, 1.02) | 0.19 | 0.98 (0.90, 1.07) | 0.72 | 0.99 (0.96, 1.02) | 0.47 |
| Alanine | 0.98 (0.94, 1.03) | 0.44 | 1.01 (0.92, 1.09) | 0.90 | 1.00 (0.97, 1.04) | 0.83 |
| Arginine | 0.98 (0.94, 1.03) | 0.50 | 1.00 (0.92, 1.09) | 0.92 | 1.00 (0.97, 1.04) | 0.78 |
| Asparagine | 0.97 (0.93, 1.02) | 0.21 | 0.96 (0.88, 1.05) | 0.40 | 0.99 (0.95, 1.02) | 0.36 |
| Aspartic acid | 0.99 (0.94, 1.03) | 0.58 | 1.00 (0.92, 1.09) | 0.98 | 1.01 (0.97, 1.04) | 0.68 |
| Cystine | 0.98 (0.94, 1.02) | 0.34 | 0.99 (0.91, 1.07) | 0.74 | 0.99 (0.96, 1.02) | 0.43 |
| Glutamine | 0.96 (0.92, 1.00) | 0.07 | 0.93 (0.86, 1.02) | 0.12 | 0.95 (0.92, 0.98) | 0.004 |
| Glutamic acid | 0.97 (0.93, 1.01) | 0.15 | 0.98 (0.90, 1.07) | 0.68 | 0.99 (0.96, 1.02) | 0.49 |
| Glycine | 0.99 (0.95, 1.03) | 0.64 | 1.02 (0.94, 1.11) | 0.66 | 1.01 (0.98, 1.04) | 0.54 |
| Proline | 0.95 (0.91, 1.00) | 0.03 | 0.97 (0.89, 1.05) | 0.47 | 0.96 (0.93, 0.99) | 0.01 |
| Serine | 0.96 (0.92, 1.01) | 0.09 | 0.98 (0.90, 1.07) | 0.66 | 0.98 (0.95, 1.01) | 0.15 |
| Tyrosine | 0.96 (0.92, 1.00) | 0.07 | 0.98 (0.91, 1.07) | 0.71 | 0.98 (0.95, 1.01) | 0.16 |
| Hydroxyproline | 1.02 (0.98, 1.07) | 0.30 | 1.02 (0.94, 1.12) | 0.59 | 1.04 (1.01, 1.08) | 0.012 |
| Taurine | 0.99 (0.95, 1.04) | 0.71 | 1.01 (0.93, 1.10) | 0.86 | 1.01 (0.98, 1.04) | 0.56 |

1. Analyses were stratified by age (in years) and follow-up intervals, and adjusted for ethnicity (white, non-white), marital status (married, widowed, divorced/separated, unknown), smoking status (never, past, current: 1–14, 15–24, ≥25 cigarettes/day, unknown status or cigarette number), alcohol intake (never drinkers, 0.1-4.9, 5.0-9.9, 10.0-14.9, 15.0+ g/d), physical activity (<3, 3–9, 9–18, 18–27, 27–42, ≥42 metabolic equivalents/week, unknown), menopausal status and postmenopausal hormone use (premenopausal, postmenopausal: never, past, current users, NHS and NHSII only), oral contraception use (never, past, current user, NHSII only), multivitamin use (no, yes), aspirin use (no, yes), body mass index (<23, 23–24.9, 25–29.9, 30–34.9, ≥35 kg/m2, missing), baseline history of hypertension (no, yes), baseline hypercholesteremia (no, yes), baseline diabetes (no, yes), and energy intake (fifths of intake). Results from NHS, NHSII and HPFS were pooled using a fixed-effects meta-analysis.
2. P-values estimated by fitting the continuous intakes of dietary amino acids in the Cox regression.

Supplementary table 15: Hazard ratios (95% confidence intervals) for total stroke (5997 cases) by per SD difference of energy-adjusted grams of dietary amino acids significantly associated with risk, further adjusting for other dietary amino acids.

|  |  | **Glutamine** | | |  |  | **Proline** | | |  |  | **Hydroxyproline** | | |  |
| --- | --- | --- | --- | --- | --- | --- | --- | --- | --- | --- | --- | --- | --- | --- | --- |
|  | ***ρ*^1^** | | **HR (95% CI)^2^** | ***p*-value^3^** | | ***ρ*^1^** | | **HR (95% CI)^2^** | ***p*-value^3^** | | ***ρ*^1^** | | **HR (95% CI)^2^** | ***p*-value^3^** | |
| Multivariable |  | | 0.94 (0.91, 0.97) | <0.001 | |  | | 0.96 (0.93, 0.99) | 0.004 | |  | | 1.05 (1.02, 1.06) | 0.002 | |
| *Further adjusted for* | | |  |  | |  | |  |  | |  | |  |  | |
| Isoleucine | 0.62 | | 0.92 (0.88, 0.95) | <0.001 | | 0.86 | | 0.89 (0.84, 0.95) | <0.001 | | 0.61 | | 1.09 (1.05, 1.14) | <0.001 | |
| Leucine | 0.64 | | 0.92 (0.88, 0.96) | <0.001 | | 0.90 | | 0.89 (0.84, 0.95) | <0.001 | | 0.60 | | 1.10 (1.06, 1.14) | <0.001 | |
| Valine | 0.63 | | 0.92 (0.89, 0.96) | <0.001 | | 0.90 | | 0.89 (0.84, 0.95) | 0.001 | | 0.57 | | 1.09 (1.05, 1.13) | <0.001 | |
| Histidine | 0.57 | | 0.92 (0.88, 0.95) | <0.001 | | 0.79 | | 0.91 (0.87, 0.95) | <0.001 | | 0.73 | | 1.12 (1.07, 1.18) | <0.001 | |
| Lysine | 0.53 | | 0.92 (0.89, 0.95) | <0.001 | | 0.80 | | 0.91 (0.86, 0.95) | <0.001 | | 0.71 | | 1.11 (1.06, 1.16) | <0.001 | |
| Methionine | 0.56 | | 0.92 (0.88, 0.95) | <0.001 | | 0.80 | | 0.91 (0.87, 0.96) | <0.001 | | 0.70 | | 1.11 (1.06, 1.15) | <0.001 | |
| Phenylalanine | 0.69 | | 0.92 (0.88, 0.96) | <0.001 | | 0.88 | | 0.91 (0.86, 0.97) | 0.003 | | 0.56 | | 1.09 (1.05, 1.14) | <0.001 | |
| Threonine | 0.57 | | 0.92 (0.88, 0.95) | <0.001 | | 0.79 | | 0.90 (0.86, 0.95) | <0.001 | | 0.68 | | 1.10 (1.06, 1.15) | <0.001 | |
| Tryptophan | 0.66 | | 0.91 (0.88, 0.95) | <0.001 | | 0.82 | | 0.89 (0.85, 0.94) | <0.001 | | 0.56 | | 1.09 (1.05, 1.13) | <0.001 | |
| Alanine | 0.53 | | 0.92 (0.89, 0.95) | <0.001 | | 0.71 | | 0.92 (0.89, 0.96) | <0.001 | | 0.76 | | 1.12 (1.07, 1.18) | <0.001 | |
| Arginine | 0.52 | | 0.92 (0.89, 0.95) | <0.001 | | 0.66 | | 0.93 (0.90, 0.97) | <0.001 | | 0.71 | | 1.11 (1.06, 1.16) | <0.001 | |
| Asparagine | 0.61 | | 0.92 (0.88, 0.96) | <0.001 | | 0.81 | | 0.92 (0.88, 0.97) | 0.001 | | 0.52 | | 1.08 (1.05, 1.13) | <0.001 | |
| Aspartic acid | 0.47 | | 0.92 (0.89, 0.95) | <0.001 | | 0.66 | | 0.93 (0.89, 0.96) | <0.001 | | 0.74 | | 1.10 (1.05, 1.16) | <0.001 | |
| Cystine | 0.69 | | 0.92 (0.88, 0.96) | <0.001 | | 0.79 | | 0.95 (0.91, 0.99) | 0.019 | | 0.43 | | 1.10 (1.06, 1.14) | <0.001 | |
| Glutamine | - | | - | - | | 0.76 | | 1.01 (0.96, 1.05) | 0.78 | | 0.15 | | 1.06 (1.02, 1.09) | <0.001 | |
| Glutamic acid | 0.52 | | 0.92 (0.89, 0.96) | <0.001 | | 0.82 | | 0.91 (0.87, 0.96) | <0.001 | | 0.65 | | 1.10 (1.06, 1.15) | <0.001 | |
| Glycine | 0.50 | | 0.92 (0.89, 0.95) | <0.001 | | 0.62 | | 0.93 (0.90, 0.97) | <0.001 | | 0.81 | | 1.14 (1.08, 1.20) | <0.001 | |
| Proline | 0.76 | | 0.93 (0.89, 0.98) | 0.003 | | - | | - | - | | 0.31 | | 1.07 (1.03, 1.10) | <0.001 | |
| Serine | 0.68 | | 0.92 (0.88, 0.96) | <0.001 | | 0.91 | | 0.89 (0.83, 0.96) | 0.002 | | 0.52 | | 1.09 (1.05, 1.13) | <0.001 | |
| Tyrosine | 0.63 | | 0.92 (0.89, 0.96) | <0.001 | | 0.91 | | 0.88 (0.82, 0.95) | 0.001 | | 0.55 | | 1.09 (1.05, 1.13) | <0.001 | |
| Hydroxyproline | 0.15 | | 0.93 (0.90, 0.96) | <0.001 | | 0.31 | | 0.94 (0.91, 0.97) | <0.001 | | - | | - | - | |
| Taurine | 0.34 | | 0.93 (0.90, 0.96) | <0.001 | | 0.45 | | 0.95 (0.92, 0.98) | 0.001 | | 0.62 | | 1.07 (1.03, 1.11) | <0.001 | |

1. Spearman’s rho for correlation between glutamine, proline or hydroxyproline and each of the other amino acids, based on dietary data collected at mid-point of follow-up (1998 for NHS, 2003 for NHSII, 1998 for HPFS).
2. Analyses were stratified by age (in years) and follow-up intervals, and adjusted for ethnicity (white, non-white), marital status (married, widowed, divorced/separated, unknown), smoking status (never, past, current: 1–14, 15–24, ≥25 cigarettes/day, unknown status or cigarette number), alcohol intake (never drinkers, 0.1-4.9, 5.0-9.9, 10.0-14.9, 15.0+ g/d), physical activity (<3, 3–9, 9–18, 18–27, 27–42, ≥42 metabolic equivalents/week, unknown), menopausal status and postmenopausal hormone use (premenopausal, postmenopausal: never, past, current users, NHS and NHSII only), oral contraception use (never, past, current user, NHSII only), multivitamin use (no, yes), aspirin use (no, yes), body mass index (<23, 23–24.9, 25–29.9, 30–34.9, ≥35 kg/m2, missing), baseline history of hypertension (no, yes), baseline hypercholesteremia (no, yes), baseline diabetes (no, yes), and energy intake (fifths of intake). Results from NHS, NHSII and HPFS were pooled using a fixed-effects meta-analysis.
3. P-values estimated by fitting the continuous intakes of dietary amino acids in the Cox regression.

Supplementary table 16: Hazard ratios (95% confidence intervals) for ischemic stroke (3058 cases) by per SD difference of energy-adjusted grams of dietary amino acids significantly associated with risk, further adjusting for macronutrients and major dietary sources.

|  |  | **Glutamine** | | |  |  | **Proline** | | |  |
| --- | --- | --- | --- | --- | --- | --- | --- | --- | --- | --- |
|  | ***ρ*^1^** | | **HR (95% CI)^2^** | ***p*-value^3^** | | ***ρ*^1^** | | **HR (95% CI)^2^** | ***p*-value^3^** | |
| Multivariable |  | | 0.94 (0.90, 0.98) | 0.004 | |  | | 0.94 (0.90, 0.98) | 0.005 | |
| *Further adjusted for nutrients* | | | |  | |  | |  |  | |
| Total protein | 0.65 | | 0.94 (0.89, 0.99) | 0.03 | | 0.85 | | 0.92 (0.85, 0.99) | 0.03 | |
| Animal protein | 0.43 | | 0.94 (0.90, 0.99) | 0.01 | | 0.78 | | 0.91 (0.85, 0.98) | 0.008 | |
| Plant protein | 0.53 | | 0.94 (0.90, 0.98) | 0.007 | | 0.16 | | 0.94 (0.90, 0.98) | 0.005 | |
| Dairy protein | 0.33 | | 0.95 (0.91, 1.00) | 0.04 | | 0.73 | | 0.96 (0.90, 1.02) | 0.17 | |
| Saturated fat | -0.08 | | 0.94 (0.90, 0.98) | 0.004 | | 0.18 | | 0.94 (0.90, 0.98) | 0.005 | |
| Monounsaturated fat | -0.12 | | 0.94 (0.90, 0.98) | 0.005 | | 0.006 | | 0.94 (0.90, 0.98) | 0.005 | |
| Polyunsaturated fat | -0.005 | | 0.94 (0.90, 0.98) | 0.003 | | -0.03 | | 0.94 (0.90, 0.98) | 0.002 | |
| Total carbohydrates | 0.23 | | 0.94 (0.90, 0.98) | 0.004 | | -0.04 | | 0.93 (0.89, 0.98) | 0.003 | |
| Total fibre | 0.32 | | 0.94 (0.90, 0.98) | 0.005 | | 0.10 | | 0.94 (0.90, 0.98) | 0.005 | |
| *Further adjusted for food groups* | | | |  | |  | |  |  | |
| Whole grains | 0.37 | | 0.92 (0.88, 0.97) | 0.001 | | 0.14 | | 0.94 (0.90, 0.98) | 0.003 | |
| Refined grains | 0.22 | | 0.94 (0.90, 0.98) | 0.004 | | -0.06 | | 0.94 (0.90, 0.98) | 0.004 | |
| Fruits | 0.03 | | 0.94 (0.90, 0.98) | 0.005 | | -0.002 | | 0.94 (0.90, 0.98) | 0.005 | |
| Vegetables | 0.10 | | 0.93 (0.89, 0.97) | 0.002 | | 0.06 | | 0.94 (0.90, 0.98) | 0.003 | |
| Nuts and seeds | -0.01 | | 0.94 (0.90, 0.98) | 0.003 | | 0.01 | | 0.94 (0.90, 0.98) | 0.003 | |
| Legumes | 0.18 | | 0.94 (0.90, 0.98) | 0.006 | | 0.12 | | 0.94 (0.90, 0.98) | 0.006 | |
| Total dairy | 0.16 | | 0.95 (0.90, 0.99) | 0.01 | | 0.48 | | 0.95 (0.90, 1.00) | 0.04 | |
| Milk | 0.21 | | 0.94 (0.90, 0.98) | 0.009 | | 0.46 | | 0.94 (0.89, 0.98) | 0.01 | |
| Cheese | 0.09 | | 0.94 (0.90, 0.98) | 0.005 | | 0.28 | | 0.94 (0.90, 0.98) | 0.004 | |
| Yogurt | 0.12 | | 0.94 (0.90, 0.99) | 0.01 | | 0.29 | | 0.95 (0.91, 0.99) | 0.02 | |
| Eggs | 0.02 | | 0.94 (0.90, 0.98) | 0.005 | | 0.11 | | 0.94 (0.90, 0.98) | 0.004 | |
| Fish | 0.17 | | 0.95 (0.91, 0.99) | 0.02 | | 0.17 | | 0.95 (0.91, 0.99) | 0.02 | |
| Total meat | 0.09 | | 0.94 (0.90, 0.98) | 0.004 | | 0.23 | | 0.93 (0.89, 0.97) | 0.002 | |
| Red meat | -0.06 | | 0.95 (0.90, 0.99) | 0.01 | | 0.10 | | 0.94 (0.90, 0.98) | 0.005 | |
| Poultry | 0.28 | | 0.94 (0.90, 0.98) | 0.005 | | 0.30 | | 0.94 (0.90, 0.98) | 0.006 | |

1. Spearman’s rho for correlation between glutamine or proline and each of the other nutrients and food groups, based on dietary data collected at mid-point of follow-up (1998 for NHS, 2003 for NHSII, 1998 for HPFS).
2. Analyses were stratified by age (in years) and follow-up intervals, and adjusted for ethnicity (white, non-white), marital status (married, widowed, divorced/separated, unknown), smoking status (never, past, current: 1–14, 15–24, ≥25 cigarettes/day, unknown status or cigarette number), alcohol intake (never drinkers, 0.1-4.9, 5.0-9.9, 10.0-14.9, 15.0+ g/d), physical activity (<3, 3–9, 9–18, 18–27, 27–42, ≥42 metabolic equivalents/week, unknown), menopausal status and postmenopausal hormone use (premenopausal, postmenopausal: never, past, current users, NHS and NHSII only), oral contraception use (never, past, current user, NHSII only), multivitamin use (no, yes), aspirin use (no, yes), body mass index (<23, 23–24.9, 25–29.9, 30–34.9, ≥35 kg/m2, missing), baseline history of hypertension (no, yes), baseline hypercholesteremia (no, yes), baseline diabetes (no, yes), and energy intake (fifths of intake). Results from NHS, NHSII and HPFS were pooled using a fixed-effects meta-analysis.
3. P-values estimated by fitting the continuous intakes of dietary amino acids in the Cox regression.

Supplementary table 17: Hazard ratios (95% confidence intervals) for total stroke (5997 cases) by per SD difference of energy-adjusted grams of dietary amino acids significantly associated with risk, further adjusting for macronutrients and major dietary sources.

|  |  | **Glutamine** | | |  |  | **Proline** | | |  |  | **Hydroxyproline** | | |  |
| --- | --- | --- | --- | --- | --- | --- | --- | --- | --- | --- | --- | --- | --- | --- | --- |
|  | ***ρ*^1^** | | **HR (95% CI)^2^** | ***p*-value^3^** | | ***ρ*^1^** | | **HR (95% CI)^2^** | ***p*-value^3^** | | ***ρ*^1^** | | **HR (95% CI)^2^** | ***p*-value^3^** | |
| Multivariable |  | | 0.94 (0.91, 0.97) | <0.001 | |  | | 0.96 (0.93, 0.99) | 0.004 | |  | | 1.05 (1.02, 1.06) | 0.002 | |
| *Further adjusted for nutrients* | | | |  | |  | |  |  | |  | |  |  | |
| Total protein | 0.65 | | 0.92 (0.88, 0.95) | <0.001 | | 0.85 | | 0.91 (0.86, 0.96) | 0.001 | | 0.64 | | 1.11 (1.06, 1.15) | <0.001 | |
| Animal protein | 0.43 | | 0.92 (0.89, 0.95) | <0.001 | | 0.78 | | 0.89 (0.85, 0.94) | <0.001 | | 0.73 | | 1.10 (1.06, 1.16) | <0.001 | |
| Plant protein | 0.53 | | 0.95 (0.92, 0.98) | 0.002 | | 0.16 | | 0.96 (0.93, 0.99) | 0.003 | | -0.22 | | 1.04 (1.00, 1.07) | 0.04 | |
| Dairy protein | 0.33 | | 0.95 (0.91, 0.98) | 0.001 | | 0.73 | | 0.98 (0.94, 1.02) | 0.34 | | -0.18 | | 1.04 (1.01, 1.07) | 0.02 | |
| Saturated fat | -0.08 | | 0.94 (0.91, 0.97) | <0.001 | | 0.18 | | 0.95 (0.93, 0.98) | 0.003 | | 0.46 | | 1.04 (1.01, 1.08) | 0.01 | |
| Monounsaturated fat | -0.12 | | 0.94 (0.91, 0.97) | <0.001 | | 0.006 | | 0.96 (0.93, 0.99) | 0.005 | | 0.41 | | 1.05 (1.01, 1.08) | 0.006 | |
| Polyunsaturated fat | -0.005 | | 0.93 (0.90, 0.96) | <0.001 | | -0.03 | | 0.95 (0.92, 0.98) | <0.001 | | 0.19 | | 1.05 (1.02, 1.08) | 0.002 | |
| Total carbohydrates | 0.23 | | 0.94 (0.91, 0.97) | <0.001 | | -0.04 | | 0.95 (0.92, 0.98) | <0.001 | | -0.42 | | 1.05 (1.01, 1.10) | 0.01 | |
| Total fibre | 0.32 | | 0.94 (0.91, 0.97) | <0.001 | | 0.10 | | 0.96 (0.93, 0.99) | 0.005 | | -0.21 | | 1.04 (1.01, 1.08) | 0.01 | |
| *Further adjusted for food groups* | | | |  | |  | |  |  | |  | |  |  | |
| Whole grains | 0.37 | | 0.93 (0.90, 0.96) | <0.001 | | 0.14 | | 0.96 (0.93, 0.99) | 0.005 | | -0.16 | | 1.05 (1.02, 1.08) | 0.004 | |
| Refined grains | 0.22 | | 0.94 (0.91, 0.97) | <0.001 | | -0.06 | | 0.95 (0.93, 0.98) | 0.003 | | -0.11 | | 1.05 (1.02, 1.08) | 0.004 | |
| Fruits | 0.03 | | 0.94 (0.91, 0.97) | <0.001 | | -0.002 | | 0.96 (0.93, 0.99) | 0.004 | | -0.19 | | 1.05 (1.02, 1.08) | 0.003 | |
| Vegetables | 0.10 | | 0.93 (0.90, 0.96) | <0.001 | | 0.06 | | 0.95 (0.93, 0.98) | 0.003 | | -0.01 | | 1.05 (1.02, 1.08) | 0.003 | |
| Nuts and seeds | -0.01 | | 0.93 (0.91, 0.96) | <0.001 | | 0.01 | | 0.95 (0.93, 0.98) | 0.002 | | -0.05 | | 1.05 (1.01, 1.08) | 0.006 | |
| Legumes | 0.18 | | 0.94 (0.91, 0.97) | <0.001 | | 0.12 | | 0.96 (0.93, 0.99) | 0.004 | | 0.005 | | 1.05 (1.02, 1.08) | 0.002 | |
| Total dairy | 0.16 | | 0.94 (0.91, 0.97) | <0.001 | | 0.48 | | 0.97 (0.93, 1.00) | 0.059 | | -0.14 | | 1.04 (1.01, 1.08) | 0.009 | |
| Milk | 0.21 | | 0.94 (0.91, 0.97) | <0.001 | | 0.46 | | 0.95 (0.92, 0.99) | 0.013 | | -0.16 | | 1.05 (1.01, 1.08) | 0.005 | |
| Cheese | 0.09 | | 0.94 (0.91, 0.97) | <0.001 | | 0.28 | | 0.96 (0.93, 0.99) | 0.006 | | 0.03 | | 1.05 (1.02, 1.08) | 0.003 | |
| Yogurt | 0.12 | | 0.94 (0.91, 0.97) | <0.001 | | 0.29 | | 0.97 (0.94, 1.00) | 0.03 | | -0.15 | | 1.04 (1.01, 1.08) | 0.010 | |
| Eggs | 0.02 | | 0.94 (0.91, 0.97) | <0.001 | | 0.11 | | 0.96 (0.93, 0.98) | 0.003 | | 0.15 | | 1.05 (1.01, 1.08) | 0.004 | |
| Fish | 0.17 | | 0.94 (0.91, 0.97) | <0.001 | | 0.17 | | 0.96 (0.93, 0.99) | 0.007 | | 0.19 | | 1.05 (1.02, 1.09) | 0.001 | |
| Total meat | 0.09 | | 0.94 (0.91, 0.96) | <0.001 | | 0.23 | | 0.95 (0.92, 0.98) | <0.001 | | 0.76 | | 1.02 (0.96, 1.08) | 0.56 | |
| Red meat | -0.06 | | 0.94 (0.92, 0.98) | <0.001 | | 0.10 | | 0.96 (0.93, 0.99) | 0.004 | | 0.68 | | 1.01 (0.97, 1.05) | 0.70 | |
| Poultry | 0.28 | | 0.93 (0.90, 0.97) | <0.001 | | 0.30 | | 0.96 (0.93, 0.99) | 0.006 | | 0.42 | | 1.07 (1.03, 1.10) | <0.001 | |

1. Spearman’s rho for correlation between glutamine, proline or hydroxyproline and each of the other nutrients and food groups, based on dietary data collected at mid-point of follow-up (1998 for NHS, 2003 for NHSII, 1998 for HPFS).
2. Analyses were stratified by age (in years) and follow-up intervals, and adjusted for ethnicity (white, non-white), marital status (married, widowed, divorced/separated, unknown), smoking status (never, past, current: 1–14, 15–24, ≥25 cigarettes/day, unknown status or cigarette number), alcohol intake (never drinkers, 0.1-4.9, 5.0-9.9, 10.0-14.9, 15.0+ g/d), physical activity (<3, 3–9, 9–18, 18–27, 27–42, ≥42 metabolic equivalents/week, unknown), menopausal status and postmenopausal hormone use (premenopausal, postmenopausal: never, past, current users, NHS and NHSII only), oral contraception use (never, past, current user, NHSII only), multivitamin use (no, yes), aspirin use (no, yes), body mass index (<23, 23–24.9, 25–29.9, 30–34.9, ≥35 kg/m2, missing), baseline history of hypertension (no, yes), baseline hypercholesteremia (no, yes), baseline diabetes (no, yes), and energy intake (fifths of intake). Results from NHS, NHSII and HPFS were pooled using a fixed-effects meta-analysis.
3. P-values estimated by fitting the continuous intakes of dietary amino acids in the Cox regression.

Supplementary table 18: Hazard ratios (95% confidence intervals) for total stroke by per SD difference of energy-adjusted grams of dietary amino acids significantly associated with risk, stratified by key covariates.

|  | **HR (95% CI)^1^** | ***p*-value^2^** | **HR (95% CI)^1^** | ***p*-value^2^** | ***p*-interaction^3^** |
| --- | --- | --- | --- | --- | --- |
| **Age** | Age below 55 years | | Age 55 years or older | |  |
| Cases/person-years | 683/2,394,569 |  | 5314/2,569,715 |  |  |
| Glutamine | 0.92 (0.84, 1.00) | 0.05 | 0.94 (0.91, 0.97) | <0.001 | 0.64 |
| Proline | 0.98 (0.90, 1.07) | 0.70 | 0.95 (0.92, 0.98) | 0.003 | 0.79 |
| Hydroxyproline | 1.00 (0.92, 1.09) | 0.93 | 1.06 (1.02, 1.09) | 0.001 | 0.48 |
|  |  |  |  |  |  |
| **BMI** | Normal weight (<25kg/m^2^) | | Overweight (≥25 kg/m^2^) | |  |
| Cases/person-years | 2573/2,358,393 |  | 3417/2,581,743 |  |  |
| Glutamine | 0.93 (0.89, 0.97) | 0.002 | 0.94 (0.91, 0.98) | 0.006 | 0.42 |
| Proline | 0.94 (0.90, 0.99) | 0.02 | 0.97 (0.93, 1.01) | 0.10 | 0.33 |
| Hydroxyproline | 1.02 (0.97, 1.07) | 0.39 | 1.07 (1.02, 1.11) | 0.002 | 0.15 |
|  |  |  |  |  |  |
| **Smoking status** | Never smokers |  | Ever smokers |  |  |
| Cases/person-years | 2519/2,650,509 |  | 3369/2,253,886 |  |  |
| Glutamine | 0.94 (0.89, 0.98) | 0.009 | 0.93 (0.90, 0.97) | 0.001 | 0.56 |
| Proline | 0.95 (0.91, 1.00) | 0.03 | 0.96 (0.92, 1.00) | 0.042 | 0.80 |
| Hydroxyproline | 1.00 (0.96, 1.05) | 0.89 | 1.08 (1.04, 1.13) | <0.001 | 0.07 |
|  |  |  |  |  |  |
| **Alcohol consumption** | Never/occasional drinkers (<5g/day) | | Regular drinkers (≥5g/day) | |  |
| Cases/person-years | 3566/3,283,161 |  | 2431/1,681,124 |  |  |
| Glutamine | 0.94 (0.90, 0.98) | 0.002 | 0.93 (0.89, 0.98) | 0.01 | 0.57 |
| Proline | 0.96 (0.93, 1.00) | 0.03 | 0.95 (0.91, 1.00) | 0.07 | 0.54 |
| Hydroxyproline | 1.03 (1.00, 1.08) | 0.09 | 1.08 (1.02, 1.14) | 0.005 | 0.41 |
|  |  |  |  |  |  |
| **Physical activity** | Below median | | Above median | |  |
| Cases/person-years | 1526/2,275,870 |  | 1526/2,275,870 |  |  |
| Glutamine | 0.95 (0.91, 0.99) | 0.01 | 0.92 (0.88, 0.97) | <0.001 | 0.79 |
| Proline | 0.96 (0.92, 1.00) | 0.04 | 0.96 (0.92, 1.00) | 0.059 | 0.51 |
| Hydroxyproline | 1.05 (1.01, 1.10) | 0.02 | 1.05 (1.00, 1.10) | 0.054 | 0.47 |
|  |  |  |  |  |  |
| **Multivitamin use** | No | | Yes | |  |
| Cases/person-years | 1277/2,280,340 |  | 1781/2,683,941 |  |  |
| Glutamine | 0.93 (0.89, 0.98) | 0.002 | 0.94 (0.90, 0.98) | 0.005 | 0.98 |
| Proline | 0.95 (0.91, 0.99) | 0.03 | 0.96 (0.93, 1.00) | 0.07 | 0.93 |
| Hydroxyproline | 1.04 (0.99, 1.09) | 0.11 | 1.06 (1.01, 1.10) | 0.01 | 0.28 |
|  |  |  |  |  |  |
| **History of hypertension, high cholesterol or diabetes** | No baseline disease history | | Baseline disease history | |  |
| Cases/person-years | 3504/3,824,596 |  | 2493/1,139,688 |  |  |
| Glutamine | 0.96 (0.92, 1.01) | 0.08 | 0.91 (0.87, 0.95) | <0.001 | 0.90 |
| Proline | 0.97 (0.93, 1.01) | 0.12 | 0.95 (0.91, 0.99) | 0.02 | 0.88 |
| Hydroxyproline | 1.04 (0.99, 1.08) | 0.12 | 1.07 (1.03, 1.12) | 0.002 | 0.08 |

1. Analyses were conducted in the particular subset only, with the model stratified by age (in years) and follow-up intervals, and adjusted for ethnicity (white, non-white), marital status (married, widowed, divorced/separated, unknown), smoking status (never, past, current: 1–14, 15–24, ≥25 cigarettes/day, unknown status or cigarette number), alcohol intake (never drinkers, 0.1-4.9, 5.0-9.9, 10.0-14.9, 15.0+ g/d), physical activity (<3, 3–9, 9–18, 18–27, 27–42, ≥42 metabolic equivalents/week, unknown), menopausal status and postmenopausal hormone use (premenopausal, postmenopausal: never, past, current users, NHS and NHSII only), oral contraception use (never, past, current user, NHSII only), multivitamin use (no, yes), aspirin use (no, yes), body mass index (<23, 23–24.9, 25–29.9, 30–34.9, ≥35 kg/m2, missing), baseline history of hypertension (no, yes), baseline hypercholesteremia (no, yes), baseline diabetes (no, yes), and energy intake (fifths of intake). Results from NHS, NHSII and HPFS were pooled using a fixed-effects meta-analysis.
2. P-values estimated by fitting the continuous intakes of dietary amino acids in the Cox regression.
3. P-interaction estimated by fitting an interaction term of the stratifying variable with the exposure of interest in the Cox regression.
